# Supplementary material for: Retrospective, multicohort analysis of the Clinical Practice Research Datalink (CPRD) to determine differences in the cost of medication wastage, dispensing fees and prescriber time of issuing either short (<60 days) or long (≥60 days) prescription lengths in primary care for common, chronic conditions in the UK
Source: BMJ Open. 2017 Dec 4;7(12):e019382. doi: 10.1136/bmjopen-2017-019382 (PMC5719293; doi:10.1136/bmjopen-2017-019382)
Supplement: Supplementary file 1 [file bmjopen-2017-019382supp001.pdf]

Appendix I – CPRD product code lists of the potentially prescribed medications for each of the five case study conditions

Appendix I is available from the authors upon request as an Excel file containing five worksheets (one for each of the five case study conditions). Within each worksheet the lists of product codes obtained from the CPRD Research Applications Code Browser Version 3.0.0.0 that represent the possible medications that patients may be prescribed for the treatment of one of the five case study conditions is presented. The table below provides an example of the codes listed available in the associated Excel file. Please note that the lists in the table below are not comprehensive and readers should request the associated Excel file for the complete product code lists.

CPRD product code list

Study condition	Product code
Glucose control with oral drug therapy in type II diabetes mellitus	2219
	7912
	16602
	26218
	41558
Treatment of hypertension in type II diabetes mellitus	2
	58
	1209
	1211
	1213
Lipid management in type II diabetes mellitus	65193
	63140
	55034
	51200
	7374
Secondary prevention of myocardial infraction	59699
	56850
	34544
	3310
	26995
Treatment of depression	2525
	48065
	4690
	8831

Appendix II - Data processing of the five cohorts

Condition	Full sample (Patients)	Full sample (Rx)	ndd=0 or qty=0 (Patients)	ndd=0 or qty=0[a] (Rx)	Limited sample[b] (Patients)	Limited sample[b] (Rx)	Random sample (Patients)	Random sample (Rx)	Dropped prescription error[c] (Rx)	Final sample (Rx)	Same day switches not wastage[d] (Rx)
Glucose control with oral drug therapy in T2DM	310,391	21,091,529	170,967	4,518,765	139,424	7,135,397	50,000	2,577,282	6,483	2,570,799	548,850
Hypertension in T2DM	230,760	23,886,597	63,802	1,446,199	166,958	16,041,452	50,000	4,803,444	3,983	4,799,461	1,588,921
Lipid management in T2DM	242,741	13,388,759	36,577	776,718	206,164	11,216,086	50,000	2,718,216	913	2,717,303	NA
Secondary prevention of myocardial infraction	208,682	44,151,527	87,281	3,270,504	121,401	24,479,014	50,000	10,131,377	767	10,130,610	5,856,361
Depression	1,207,523	32,744,994	424,446	4,438,319	783,077	15,712,941	50,000	1,010,463	3,234	1,007,229	12,401

ndd – numeric daily dose; NA – not applicable; qty – quantity; Rx – prescriptions; T2DM – type II diabetes mellitus

[a]The product codes that most frequently had qty or ndd values equal to zero were: metformin 500 mg tablets (38%) and gliclazide 80 mg tablets (26%) for the T2DM cohort; ramipril 10 mg capsules (9%), bendroflumethiazide 2.5 mg tablets, Ramipril 5 mg capsules, Ramipril 2.5 mg capsules and amlodipine 5 mg tablets for the hypertension in T2DM cohort; simvastatin 40 mg tablets (38%), simvastatin 20 mg tablets (16%), atorvastatin 40 mg tablets (10%) and atorvastatin 20 mg tablets (10%) for the lipid management in T2DM cohort; aspirin 75 mg dispersible tablets (21%) and simvastatin 40 mg tablets (12%) for the secondary prevention of myocardial infarction cohort; and citalopram 20 mg tablets (13%) and fluoxetine 20 mg capsules (11%) for the depression cohort.

[b]The limited sample consists of all patients that have received at least one of the relevant prescriptions for a respective case study condition and do not have any missing or observations equal to zero for both the ndd and qty variables.

[c]Prescriptions issued on the same day for medications in the same class and with different product codes (e.g., two different selective serotonin reuptake inhibitors or two different statins) were considered prescriber error and dropped from the analysis.

[d]Prescriptions for medications with similar clinical indications from different classes issued on the same day were assumed not to incur wastage due to a treatment switch, but rather were assumed to be an add-on to therapy or concomitant therapy.

Appendix III – Description of methods used to estimate medication wastage

Wastage was defined as either a repeat prescription or new prescription based on the three types of substitutions/switches (substitutions between different dosages or formulations of the same drug substance; substitutions between drugs that are in the same class; and substitutions between drugs that have similar clinical indications from different classes) respectively, being issued prior to the expiry of the previously prescribed quantity. The volume of medication wastage from early repeat prescriptions and treatment switches was estimated for prescriptions within the 11-year study period (i.e., any prescription not falling in this time period were excluded from the analyses). This was done by dividing the total quantity entered by the GP for the prescribed product (qty) by the numeric daily dose prescribed for the event (ndd) and comparing this to the difference in the two dates associated with the event and the next prescription in the sequence. Prescriptions issued on the same day for drugs in the same class with different product codes (e.g., two different statins) were considered prescriber error and dropped from the analysis. The one exception to this was for antiplatelet drugs in the secondary prevention of myocardial infarction cohort, as it was assumed that two different antiplatelet drugs could be prescribed at the same time. In addition, prescriptions for medications with similar clinical indications from different classes issued on the same day were not counted as a switch, but rather as an add-on to existing therapy or concomitant therapy.

As treatment patterns were assessed in sequence there was the potential to overstate the amount of wastage that occurred. For example, counting every overlap of prescription days associated with repeat prescriptions of the same product as wastage (i.e., even prescriptions issued one day before the expiry of the previous prescription would be counted as one day’s worth of medication wastage or two prescriptions issued on the same day would count one prescription as entirely wasted) may overstate actual wastage. A threshold of one

year after the initial prescription in a particular series was, therefore, used to estimate wastage for early repeat prescriptions, in that any product prescribed over and above the expected quantity to be consumed within the one-year time period was considered waste. This is to account for the fact that patients may fill their prescriptions before their existing supply is exhausted, but still consume all of the previous prescription before using the new supply.

In addition, while excessive switching of drugs could appear as wastage, consistent patterns could suggest a valid, prescribed treatment regimen. To avoid the overestimation of wastage, additional effort was made to differentiate between add-ons/concomitant therapy and switches for medications with similar clinical indications from different classes (i.e., product, drug substance and drug class codes are different for two prescriptions in sequence). Generally, if the difference between the number of changes between medications with similar clinical indications from different classes and the number of unique drug classes within an annual period was ≥1, then any overlap in prescription dates was not considered wastage due to a switch, but rather as an add-on or concomitant therapy. For example, within an annual period a patient may have six changes between clinically related drugs from different classes, but these changes are only between two different drugs from different classes (i.e., two unique drug classes). Since six minus two is ≥1 these changes were not considered switches, but rather add-on/concomitant prescriptions. The rationale being that if the number of changes was large, but the number of unique drugs involved in the changes was low an add-on or concomitant therapy was being prescribed, whereas if the number of changes was large and the number of unique drugs involved in the changes was also large, switches in therapies were occurring and therefore there was the potential for wastage to occur. A similar approach was applied by Taitel et al.[1] and is the only previous study that has attempted to differentiate between add-on/concomitant prescriptions and actual switches in therapy. Additional constraints in counting overlaps in dates for two prescriptions in sequence for medications

with similar clinical indications from different classes were also applied in three of the case study conditions due to the potential for a number of the included therapies to be given concomitantly. For the glucose control in T2DM cohort overlaps in prescription dates involving metformin with drug from other classes were not counted as switches (and therefore wastage) as metformin is usually administered in combination with other classes of oral anti-diabetics.[2] For the treatment of hypertension in T2DM cohort, overlaps in prescription dates involving angiotensin-converting enzyme inhibitors and angiotensin-II receptor antagonists with either calcium-channel blockers or thiazide-like diuretics were not counted as switches as these therapies are commonly administered together as second-line therapy.[3] Finally, for the secondary prevention of myocardial infraction cohort, only overlapping prescriptions dates involving beta-blockers and angiotensin-II receptor antagonists were counted as switches as patients are likely to receive the other classes of drugs included in the analysis (angiotensin-converting enzyme inhibitors, antiplatelet drugs and statins) continuously over the course of treatment.[4]

Appendix IV – Unit prescription drug cost calculations

Drug substance	DDD (mg)	NIC from PCA (pence)	Quantity from PCA	Strength from PCA (mg)	NIC/ Quantity (£)	Cost per day[a] (£)
**Initial glucose control in type II diabetes**						
glibenclamide	7	1,072,539	279,251	5	0.04	0.05
gliclazide	60	3,322,771	195,041	60	0.17	0.17
glimepiride	2	2,650,357	685,422	2	0.04	0.04
glipizide	10	8,679,376	762,384	5	0.11	0.23
tolbutamide	1500	17,494,096	266,373	500	0.66	1.97
metformin	2000	531,088,447	111,025,385	500	0.05	0.19
acarbose	300	108,607	1,234	100	0.88	2.64
alogliptin	25	6,918,604	10,147	25	6.82	6.82
canagliflozin	200	58,466,442	447,445	100	1.31	2.61
dapagliflozin	10	191,937,124	1,468,764	10	1.31	1.31
empagliflozin	17.5	16,291,281	124,666	10	1.31	2.29
exenatide	1	10,031,280	1,470	60	68.24	1.14
linagliptin	5	271,703,001	2,287,246	5	1.19	1.19
liraglutide	1.2	337,718,696	86,033	6	39.25	7.85
lixisenatide	0.02	45,851,757	15,830	0.28	28.97	2.07
nateglinide	360	631,585	17,827	180	0.35	0.71
pioglitazone	30	144,198,158	1,125,016	30	1.28	1.28
repaglinide	4	1,620,508	246,350	2	0.07	0.13
saxagliptin	5	80,113,342	709,874	5	1.13	1.13
sitagliptin	100	670,533,026	5,644,766	100	1.19	1.19
vildagliptin	100	18,582,165	312,012	50	0.60	1.19
rosiglitazone[b]	6	-	-	-	-	2.34
guar gum[b,d]	68.5	-	-	-	-	2.34
dulaglutide	0.16	1,717,714	938	0.75	18.31	3.91
**Hypertension in type II diabetes**						
bendroflumethiazide	2.5	115,395,532	38,916,033	2.5	0.03	0.03
chlortalidone	25	410	70	50	0.06	0.03
cyclopenthiazide[b]	0.5	-	-	-	-	1.07
indapamide	2.5	40,603,410	7,849,048	2.5	0.05	0.05
xipamide	20	274,581	19,761	20	0.14	0.14
chlorothiazide	500	92,160	200	500	4.61	4.61
hydrochlorothiazide	25	743,206	4,701	25	1.58	1.58
Hydroflumethiazide[b]	25	-	-	-	-	1.07
candesartan cilexetil	8	27,228,854	6,187,293	8	0.04	0.04
eprosartan	600	7,848,330	156,629	600	0.50	0.50
irbesartan	150	15,321,402	2,527,161	150	0.06	0.06
losartan potassium	50	43,142,921	10,854,773	50	0.04	0.04
olmesartan medoxomil	20	23,454,180	507,120	20	0.46	0.46
telmisartan	40	2,105,968	429,294	40	0.05	0.05
valsartan	80	7,680,126	790,946	80	0.10	0.10

Drug substance	DDD (mg)	NIC from PCA (pence)	Quantity from PCA	Strength from PCA (mg)	NIC/ Quantity (£)	Cost per day[a] (£)
captopril	50	1,209,326	274,077	50	0.04	0.04
enalapril maleate	10	8,273,970	2,164,274	10	0.04	0.04
fosinopril sodium	15	1,717,978	32,658	10	0.53	0.79
imidapril hydrochloride	10	523,685	20,308	10	0.26	0.26
lisinopril	10	30,787,577	8,978,102	10	0.03	0.03
moexipril hydrochloride	15	22,272	896	15	0.25	0.25
perindopril erbumine	4	25,785,508	604,0253	4	0.04	0.04
perindopril arginine	4	1,125,376	53,760	5	0.21	0.17
quinapril	15	2,130,784	69,778	10	0.31	0.46
ramipril	2.5	75,859,752	18,621,897	2.5	0.04	0.04
ramipril with felodipine	2.5	456,727	5,209	2.5	0.88	0.88
trandolapril	2	4,668,257	191,955	2	0.24	0.24
cilazapril	2.5	1,662,225	8,985	5	1.85	0.93
perindopril tosilate	4	11,821	594	5	0.20	0.16
amlodipine	5	153,321,726	47,137,956	5	0.03	0.03
diltiazem hydrochloride	240	3,855,905	93,720	240	0.41	0.41
felodipine	5	63,115,662	4,197,918	5	0.15	0.15
isradipine	5	4,429,879	13,442	2.5	3.30	6.59
lacidipine	4	14,646,423	910,750	4	0.16	0.16
lercanidipine hydrochloride	10	77,973,540	3,825,663	10	0.20	0.20
nicardipine hydrochloride	90	397,902	35,119	30	0.11	0.34
nifedipine	30	3,656,530	149,471	30	0.24	0.24
nisoldipine[b]	20	-	-	-	-	0.93
verapamil hydrochloride	240	7,104,750	358,394	240	0.20	0.20
doxazosin	4	52,769,814	14,187,844	4	0.04	0.04
indoramin[d]	4.7	5,674,414	34,4947	20	0.16	0.04
prazosin	5	74,527	205	5	3.64	3.64
terazosin	5	1,372,116	136,216	5	0.10	0.10
amiloride hydrochloride	10	18,835,995	565,354	5	0.33	0.67
amiloride hydrochloride with thiazide	10	868,138	75,026	5	0.12	0.23
triamterene	100	211,025	1,511	50	1.40	2.79
triamterene with thiazide	100	160,534	4,540	50	0.35	0.71
spironolactone	75	24,174,720	4,339,112	25	0.06	0.17
atenolol	75	25,567,900	8,522,336	25	0.03	0.09
**Hyperlipidaemia in type II diabetes**						
atorvastatin	20	158,989,904	31,324,266	20	0.05	0.05
fluvastatin	60	1,475,946	163,264	20	0.09	0.27

Drug substance	DDD (mg)	NIC from PCA (pence)	Quantity from PCA	Strength from PCA (mg)	NIC/ Quantity (£)	Cost per day[a] (£)
pravastatin	30	6,780,883	1,648,896	10	0.04	0.12
rosuvastatin	10	134,267,696	2,085,108	10	0.64	0.64
simvastatin	30	15,635,680	5,206,048	10	0.03	0.09
**Secondary prevention of myocardial infraction**						
captopril	50	1,209,326	274,077	50	0.04	0.04
enalapril maleate	10	8,273,970	2,164,274	10	0.04	0.04
fosinopril sodium	15	1,717,978	32,658	10	0.53	0.79
lisinopril	10	30,787,577	8,978,102	10	0.03	0.03
perindopril erbumine	4	25,785,508	6,040,253	4	0.04	0.04
perindopril arginine	4	1,125,376	53,760	5	0.21	0.17
quinapril	15	728,515	23,878	5	0.31	0.92
ramipril	2.5	75,859,752	18,621,897	2.5	0.04	0.04
ramipril with felodipine	2.5	456,727	5,209	2.5	0.88	0.88
trandolapril	2	4,668,257	191,955	2	0.24	0.24
cilazapril	2.5	1,662,225	8,985	5	1.85	0.93
perindopril tosilate	4	11,821	594	5	0.20	0.16
acebutolol	400	1,202,284	18,079	400	0.67	0.67
atenolol	75	25,567,900	8,522,336	25	0.03	0.09
bisoprolol	10	20,420,456	5,828,018	10	0.04	0.04
carvedilol	37.5	2,684,299	577,569	6.25	0.05	0.28
metoprolol tartrate	150	15,398,869	2,318,257	50	0.07	0.20
aspirin[d]	127.5	154,533,218	52,719,924	75	0.03	0.05
clopidogrel	75	129,484,315	19,793,087	75	0.07	0.07
ticagrelor	180	190,143,810	1,950,196	90	0.97	1.95
atorvastatin	20	158,989,904	31,324,266	20	0.05	0.05
fluvastatin	60	1,475,946	163,264	20	0.09	0.27
pravastatin	30	6,780,883	1,648,896	10	0.04	0.12
rosuvastatin	10	134,267,696	2,085,108	10	0.64	0.64
simvastatin	30	15,635,680	5,206,048	10	0.03	0.09
candesartan cilexetil	8	27,228,854	6,187,293	8	0.04	0.04
eprosartan	600	7,848,330	156,629	600	0.50	0.50
irbesartan	150	15,321,402	2,527,161	150	0.06	0.06
losartan potassium	50	43,142,921	10,854,773	50	0.04	0.04
olmesartan medoxomil	20	23,454,180	507,120	20	0.46	0.46
telmisartan	40	2,105,968	429,294	40	0.05	0.05
valsartan	80	7,680,126	790,946	80	0.10	0.10
**Depression**						
amitriptyline hydrochloride	75	42,327,326	12,089,330	25	0.04	0.11
amoxapine	150	65,772	168	100	3.92	5.87
clomipramine hydrochloride	100	5,343,700	663,607	50	0.08	0.16
dosulepin hydrochloride	150	9,835,231	1,582,426	75	0.06	0.12

Drug substance	DDD (mg)	NIC from PCA (pence)	Quantity from PCA	Strength from PCA (mg)	NIC/ Quantity (£)	Cost per day[a] (£)
doxepin[c]	100	-	-	50	0.20	0.41
imipramine hydrochloride	100	4,075,545	858,036	25	0.05	0.19
lofepramine	105	39,309,425	1,177,389	70	0.33	0.50
nortriptyline	75	115,762,972	1,077,134	25	1.07	3.22
trazodone hydrochloride	300	61,816,624	718,214	150	0.86	1.72
trimipramine	150	1,672	56	50	0.30	0.90
citalopram	20	86,323,713	23,901,861	20	0.04	0.04
escitalopram	10	5,540,518	1,121,092	10	0.05	0.05
fluoxetine	20	87,922,802	23,735,904	20	0.04	0.04
fluvoxamine maleate	100	4,073,827	55,899	100	0.73	0.73
paroxetine	20	25,034,806	3,116,825	20	0.08	0.08
sertraline	50	100,699,567	15,289,272	50	0.07	0.07
isocarboxazid	15	3,453,630	12,118	10	2.85	4.28
moclobemide	300	1,057,512	22,678	300	0.47	0.47
phenelzine	60	2,647,884	117,682	15	0.23	0.90
tranylcypromine	10	45,505,295	51,406	10	8.85	8.85
agomelatine	25	8,035,533	74,999	25	1.07	1.07
duloxetine	60	276,427,017	2,904,742	60	0.95	0.95
mirtazapine	30	26,199,391	4,793,099	30	0.05	0.05
reboxetine	8	4,467,383	141,747	4	0.32	0.63
tryptophan[d]	44.6	18,495	566	50	0.33	0.29
venlafaxine	100	17,440,533	3,256,789	75	0.05	0.07

DDD – defined daily dose; NIC – net ingredient cost; PCA – Prescription Cost Analysis

[a]Calculated as (NIC/Quantity) x (DDD/Strength from PCA).

[b]Data not available within the PCA for December 2015; cost per day based on an average of the values for other drugs within the same class.

[c]Data from PCA for December 2015 deemed unreliable; NIC/Quantity derived by dividing cost of 50 mg 28-cap pack by 28.

[d]Data for DDD not available; DDD based on an average of the values for other drugs within the same class.

Appendix V – Search strategy for prescriber time data

Date of Search: *July 8, 2016*

Databases: Ovid MEDLINE (R) Epub Ahead of Print, In-process & Other Non-Indexed Citations, Ovid MEDLINE (R) Daily and Ovid MEDLINE (R) 1946 to present; Embase 1974 to 2016 July 07

1	general practitioner.ab,hw,kf,kw,ot,ti,xs.
2	GP.ab,hw,kf,kw,ot,ti,xs.
3	physician.ab,hw,kf,kw,ot,ti,xs.
4	clinician.ab,hw,kf,kw,ot,ti,xs.
5	doctor.ab,hw,kf,kw,ot,ti,xs.
6	medic.ab,hw,kf,kw,ot,ti,xs.
7	consultant.ab,hw,kf,kw,ot,ti,xs.
8	medical specialist.ab,hw,kf,kw,ot,ti,xs.
9	physician assistant.ab,hw,kf,kw,ot,ti,xs.
10	physician associate.ab,hw,kf,kw,ot,ti,xs.
11	nurse.ab,hw,kf,kw,ot,ti,xs.
12	pharmacist.ab,hw,kf,kw,ot,ti,xs.
13	healthcare professional.ab,hw,kf,kw,ot,ti,xs.
14	medical professional.ab,hw,kf,kw,ot,ti,xs.
15	medical staff.ab,hw,kf,kw,ot,ti,xs.
16	1 or 2 or 3 or 4 or 5 or 6 or 7 or 8 or 9 or 10 or 11 or 12 or 13 or 14 or 15
17	prescriber time.ab,hw,kf,kw,ot,ti,xs.
18	staff time.ab,hw,kf,kw,ot,ti,xs.
19	time utilization.ab,hw,kf,kw,ot,ti,xs.
20	time utilisation.ab,hw,kf,kw,ot,ti,xs.
21	workload.ab,hw,kf,kw,ot,ti,xs.
22	workflow.ab,hw,kf,kw,ot,ti,xs.
23	work processes.ab,hw,kf,kw,ot,ti,xs.
24	medication management.ab,hw,kf,kw,ot,ti,xs.
25	17 or 18 or 19 or 20 or 21 or 22 or 23 or 24
26	time study.ab,hw,kf,kw,ot,ti,xs.
27	time motion study.ab,hw,kf,kw,ot,ti,xs.
28	time-motion study.ab,hw,kf,kw,ot,ti,xs.
29	(time and motion method).ab,hw,kf,kw,ot,ti,xs.
30	time-and-motion method.ab,hw,kf,kw,ot,ti,xs.
31	(time and motion study).ab,hw,kf,kw,ot,ti,xs.
32	time-and-motion study.ab,hw,kf,kw,ot,ti,xs.
33	time motion analysis.ab,hw,kf,kw,ot,ti,xs.
34	time-motion analysis.ab,hw,kf,kw,ot,ti,xs.
35	(before and after study).ab,hw,kf,kw,ot,ti,xs.
36	before-and-after study.ab,hw,kf,kw,ot,ti,xs.
37	26 or 27 or 28 or 29 or 30 or 31 or 32 or 33 or 34 or 35 or 36
**38**	16 and 25 and 37 – **Total Hits = 227**

Study selection details

The targeted literature search identified a total of 227 citations. After titles and abstracts were screened 216 citations were excluded and 11 citations were reviewed in full-text. Four studies contained relevant information and the most appropriate evidence was selected from the four studies by prioritising evidence from larger sample sizes and studies that reported prescriber time for different types of prescriptions (e.g., new versus renewals) and/or different types of prescribers (e.g., general practitioner versus nurse).[5-8] It should be noted that one of the four studies identified was a systematic review and led to the identification of two additional studies with relevant information based on their reporting of mean consultation times based on large sample sizes and in different types of prescribers.[9 10]

Appendix VI - Mean values used in the comparison of total unnecessary costs

	Glucose control with oral drug therapy in T2DM		Hypertension in T2DM		Lipid management in T2DM		Secondary prevention of myocardial infraction		Depression	
**Parameters**	**<60 days**	**≥60 days**	**<60 days**	**≥60 days**	**<60 days**	**≥60 days**	**<60 days**	**≥60 days**	**<60 days**	**≥60 days**
Mean days used ($Q_{daysused}$)	31.61	75.72	32.51	86.91	32.74	122.71	31.41	102.28	27.43	65.03
Mean days wasted ($Q_{dayswasted}$)	0.86	4.96	1.23	6.98	0.63	16.21	0.96	6.56	1.87	2.69
Mean drug cost per day (£) ($C_{drug/day}$)	0.36	0.28	0.084	0.082	0.10	0.10	0.074	0.068	0.14	0.11
Mean dispensing fee cost (£) ($C_{dispensing}$)	0.92	1.05	0.90	0.93	0.90	1.00	0.90	0.97	0.91	0.96
Mean prescriber (GP) time cost (£) ($C_{prescribertime}$)	3.39	3.44	3.54	3.55	3.12	3.15	3.76	3.77	3.23	3.18
Mean prescriber (Nurse) time cost (£) ($C_{prescribertime}$)	2.28	2.29	2.31	2.32	2.21	2.22	2.37	2.37	2.24	2.23

GP – general practitioner; T2DM – type II diabetes mellitus

Appendix VII – Study protocol (16_117R) approved on June 21, 2016 by the Independent Scientific Advisory Committee (ISAC)

ISAC APPLICATION FORM

PROTOCOLS FOR RESEARCH USING THE CLINICAL PRACTICE RESEARCH DATALINK (CPRD)

ISAC use only:<br>Protocol Number<br>Date submitted	 ....<br>............................	**IMPORTANT**<br>**If you have any queries, please contact ISAC Secretariat:**<br>ISAC@cprd.com

Section A: The study

1. Study Title

Three months versus 28 day prescriptions: a retrospective analysis of CPRD data to determine differences in the cost of drug wastage, dispensing fees and prescriber time

2. Has any part of this research proposal or a related proposal been previously submitted to ISAC?

Yes ☐ No ☒

If Yes, please provide previous protocol numbers:

3. Has this protocol been peer reviewed by another Committee? (e.g. grant award or ethics committee)

Yes ☒ No ☐

If Yes, please state the name of the reviewing Committee(s) and provide an outline of the review process and outcome: Proposal peer reviewed as part of a successful application to NIHR Health Technology Assessment Programme.

4. Type of Study (please tick all the relevant boxes which apply)

Adverse Drug Reaction/Drug Safety ☐
Drug Effectiveness ☐
Health/Public Health Services Research ☒
Drug Utilisation ☒
Pharmacoeconomics ☒
Disease Epidemiology ☐
Methodological ☐
Post-authorisation Safety ☐
Other* ☐

*Please specify the type of study in the lay summary

5. This study is intended for (please tick all the relevant boxes which apply)**:**

Publication in peer reviewed journals ☒
Presentation at company/institutional meetings ☐
Other: Publication in NIHR HTA Monograph
Presentation at scientific conference ☒
Regulatory purposes ☐

Section B: The Investigators

6. Chief Investigator (full name, job title, organisation name & e-mail address for correspondence- see guidance notes for eligibility)

Name: Ed Wilson
Job title: Senior Research Associate in Health Economics
Organisation: Cambridge Centre for Health Services Research, University of Cambridge
Email: ew442@medschl.cam.ac.uk

CV has been previously submitted to ISAC ☐ **CV number:**
A new CV is being submitted with this protocol ☒
An updated CV is being submitted with this protocol ☐

7. Affiliation (full address)

Institute of Public Health, Cambridge CB2 0SR, UK

8. Corresponding Applicant

Name: Brett Doble
Job title: Research Associate in Health Economics
Organisation: Cambridge Centre for Health Services Research, University of Cambridge
Email: brett.doble@medschl.cam.ac.uk

Same as chief investigator ☐
CV has been previously submitted to ISAC ☐ **CV number:**
A new CV is being submitted with this protocol ☒
An updated CV is being submitted with this protocol ☐

9. List of all investigators/collaborators (*please list the full names, affiliations and e-mail addresses* of all collaborators, other than the Chief Investigator*)

Other investigator: Rupert Payne,

Job title: Consultant Senior Lecturer in Primary Health Care
Organisation: University of Bristol
Email: rupert.payne@bristol.ac.uk

CV has been previously submitted to ISAC ☒ **CV number:** 177_15CEP
A new CV is being submitted with this protocol ☐
An updated CV is being submitted with this protocol ☐

Other investigator: Sarah King
Job title: Visiting Fellow
Organisation: University of Cambridge
Email: sek23@cam.ac.uk
CV has been previously submitted to ISAC ☐ **CV number:**
A new CV is being submitted with this protocol ☒
An updated CV is being submitted with this protocol ☐

[Please add more investigators as necessary] **Please note that your ISAC application form and protocol **must** be copied to all e-mail addresses listed above at the time of submission of your application to the ISAC mailbox. Failure to do so will result in delays in the processing of your application.*

10. Conflict of interest statement* (please provide a draft of the conflict (or competing) of interest (COI) statement that you intend to include in any publication which might result from this work)
The authors do not have any conflict of interest.
**Please refer to the International Committee of Medical Journal Editors (ICMJE) for guidance on what constitutes a COI*

11. Experience/expertise available (please complete the following questions to indicate the experience/expertise available within the team of investigators/collaborators actively involved in the proposed research, including the analysis of data and interpretation of results

	**Previous GPRD/CPRD Studies**	**Publications using GPRD/CPRD data**
None	☐	☒
1-3	☐	☐
> 3	☒	☐

	**Yes**	**No**
**Is statistical expertise available within the research team?**<br>*If yes, please indicate the name(s) of the relevant investigator(s)*<br>Rupert Payne and Ed Wilson with additional support provided by the Cambridge CPRD User Group, which includes Senior Statistician Dr. Katie Saunders.	☒	☐
**Is experience of handling large data sets (>1 million records) available within the research team?**<br>*If yes, please indicate the name(s) of the relevant investigator(s)*<br>Rupert Payne, Brett Doble<br>• Rupert Payne previously managed Cambridge's institutional license for CPRD, and is familiar with the CPRD GOLD dataset, analysis of HES records, and has relevant experience from analysis of other large, linked primary-secondary care datasets. He also has extensive programming experience as well as database management skills.<br>• Brett Doble has had experience managing, cleaning, and analysing large datasets in Australia (e.g., PBS, MBS, VAED, VEMD) during his PhD studies at Monash University. He has also recently had experience analysing UK GP practice prescribing data from the Health & Social Care Information Centre.	☒	☐
**Is experience of practising in UK primary care available within the research team?**<br>*If yes, please indicate the name(s) of the relevant investigator(s)*<br>Rupert Payne<br>• Dr Rupert Payne is a practising GP and Consultant Senior Lecturer in Primary Care at the School of Social and Community Medicine, University of Bristol	☒	☐

12. References relating to your study

Please list up to 3 references (most relevant) relating to your proposed study:

- Domino ME, Olinick J, Sleath B, Leinwand S, Byrns PJ, Carey T. Restricting patients' medication supply to one month: Saving or wasting money. Am J Health-Syst Pharm 2004;61:1375-1379.

**Section C: Access to the data**
**13. Financial Sponsor of study**<br><br>Pharmaceutical Industry ☐ *Please specify:* Academia ☒ *Please specify:* NIHR Health Technology Assessment Programme<br>Government / NHS ☐ *Please specify:* Charity ☐ *Please specify:*<br>Other ☐ *Please specify:* None ☐
**14. Type of Institution carrying out the analyses**<br><br>Pharmaceutical Industry ☐ *Please specify:* Academia ☒ *Please specify:* Cambridge Centre for Health Services Research, University of Cambridge<br>Government Department ☐ *Please specify:* Research Service Provider ☐ *Please specify:*<br>NHS ☐ *Please specify:* Other ☐ *Please specify:*

15. Data source

The sponsor has direct access to CPRD GOLD and will extract the relevant data* ☒

A data set will be supplied by CPRD** ☐

CPRD has been commissioned to extract the relevant data and to perform the analyses ☐
Other ☐ *Please specify:*

*If data sources other than CPRD GOLD are required, these will be supplied by CPRD
** Please note that datasets provided by CPRD are limited in size. Applicants should contact CPRD (KC@CPRD.com) if a dataset of >300,000 patients is required.

16. Primary care data (please specify which primary care data set(s) are required)
Vision only (Default for CPRD studies) ☒
EMIS® only* ☐
Both Vision and EMIS®* ☐

Note: Vision and EMIS are different clinical systems, Vision data has traditionally been used for CPRD, EMIS is currently undergoing beta-testing.
**Investigators requiring the use of EMIS data must discuss the study with a member of CPRD staff before submitting an ISAC application*
Please list below the name of the person/s at the CPRD with whom you have discussed your request for EMIS data:

Section D: Data linkage

17. Does this protocol also seek access to data held under the CPRD Data Linkage Scheme?

Yes* ☐ No ☒

If No, please move to section E.

**Investigators requiring linked data must discuss the study with a member of CPRD staff. It is important to be aware that linked data are not available for all patients in CPRD GOLD, the coverage periods for each data source may differ and charges may be applied. Please contact the CPRD Research Team on +44 (20) 3080 6383 or email kc@cprd.com to discuss your requirements before submitting your application.*
Please list below the name of the person/s at the CPRD with whom you have discussed your request:

Please note that as part of the ISAC review of linkages, the protocol may be shared - in confidence - with a representative of the requested linked data set(s) and summary details may be shared - in confidence - with the Confidentiality Advisory Group of the Health Research Authority.

18. Please select the source(s) of linked data being requested:

☐ ONS Mortality Data
☐ Inpatient Hospital Episode Statistics
☐ Outpatient Hospital Episode Statistics
☐ NCDR Cancer Registry Data*
☐ MINAP
☐ Mother Baby Link

☐ Index of Multiple Deprivation
☐ Townsend Score
☐ Other** *Please specify:*

Please note that applicants seeking access to cancer registry data must provide consent for publication of their study title and study institution on the UK Cancer Registry website. They must also complete a* ***Cancer Dataset Agreement Form* *(available from CPRD) and provide a* ***System level Security Policy*** *for each organisation involved in the study.*

*** If "Other" is specified, please name an individual in CPRD that this linage has been discussed with.*

19. Total number of linked datasets requested including CPRD GOLD:

20. Is linkage to a local dataset with <1 million patients being requested?

Yes* ☐ No ☐

** If yes, please provide further details:*

21. If you have requested linked data sets, please indicate whether the Chief Investigator or any of the collaborators listed in response to question 5 above, have access to any of the linked datasets in a patient identifiable form, or associated with a patient index.

Yes* ☐ No ☐

** If yes, please provide further details:*

22. Does this study involve linking to patient *identifiable* data from other sources?

Yes ☐ No ☐

Section E: Validation/verification

23. Does this protocol describe a purely observational study using CPRD data (this may include the review of anonymised free text)?

Yes* ☒ No** ☐

** Yes: If you will be using data obtained from the CPRD Group, this study does not require separate ethics approval from an NHS Research Ethics Committee.*
*** No: You may need to seek separate ethics approval from an NHS Research Ethics Committee for this study. The ISAC will provide advice on whether this may be needed.*

24. Does this study require anonymised free text?

Yes* ☐ No ☒

**Please note that work involving free text can only be performed on the July 2013 CPRD GOLD database build or earlier versions. CPRD can provide further advice on the use of anonymised free text.*

25. Does this protocol involve requesting any additional information from GPs?

Yes* ☐ No ☒

* *Please indicate what will be required:*
Completion of questionnaires by the GP[ψ] Yes ☐ No ☐
Provision of anonymised records (e.g. hospital discharge summaries) Yes ☐ No ☐
Other (please describe)

ψ Any questionnaire for completion by GPs or other health care professional must be approved by ISAC before circulation for completion.

26. Does this study require contact with patients in order for them to complete a questionnaire?

Yes* ☐ No ☒

**Please note that any questionnaire for completion by patients must be approved by ISAC before circulation for completion.*

27. Does this study require contact with patients in order to collect a sample?

Yes* ☐ No ☒

** Please state what will be collected:*

Section F: Signatures

28. Signature from the Chief Investigator

I confirm that the above information is to the best of my knowledge accurate, and I have read and understood the guidance to applicants.

Name: Ed Wilson Date: 25/05/16 E. signature (type name): ED WILSON

PROTOCOL INFORMATION

In order to help ensure that protocols submitted for review contain adequate information for protocol evaluation, ISAC have produced guidance on the content of protocols for research using CPRD data. This guidance is available on the CPRD website (www.cprd.com/ISAC). All protocols using CPRD data which are submitted for review by ISAC must contain information on all the areas detailed below. If a specific area required by ISAC is not applicable to your protocol, please provide the justification underneath the relevant heading.

The protocol section (next page) has pre-defined headings and the protocol must be written using these headings. Additional headings are not acceptable; however, supplementary information may be placed in one or more of the appendices providing this information is essential and an appropriate reference to it is made within the protocol. Unless very short, codes lists should be placed in an Appendix. Applications will be regarded as invalid and returned to the applicant if any of the headings below are missing or if additional sections are included.

Please note that ISAC will not consider any application where the protocol exceeds 12 pages (excluding sections A-F of the application form and annexes). Annexes should be kept to a minimum and contain only vital information that could not be provided in the main protocol section. A font-size of at least 12 should be used. Protocols not exceeding 15 pages would be acceptable if ISAC has required a resubmission where additional information is requested.

Please note, your protocol will not be reviewed by ISAC if it falls short of the above requirements. You are advised to speak to the Secretariat if you have any queries.

Voluntary registration of ISAC approved studies:

Epidemiological studies are increasingly being included in registries of research around the world, including those primarily set up for clinical trials. To increase awareness amongst researchers of ongoing research, ISAC encourages voluntary registration of epidemiological research conducted using MHRA databases. This will not replace information on ISAC approved protocols that may be published on the CPRD website. It is for the applicant to determine the most appropriate registry for their study. Please inform the ISAC secretariat that you have registered a protocol and provide the location.

Protocol Section

The following headings **must** be used to form the basis of the protocol. Pages should be numbered. All abbreviations must be defined on first use.

A. Lay Summary (Max. 200 words)

Please provide a succinct overview of your proposed research in plain English i.e. non-technical language. This should cover the background, purpose of the study and the potential importance of the findings. References and abbreviations should be avoided. If you have ticked the "other" box in response to question 4 on the application form, up to an additional 100 words should be used to describe the benefit to public health expected from the study.

In the NHS, general practitioners (GPs) have been encouraged to issue prescriptions of shorter duration (e.g., 28 days), to reduce drug expenditure and wastage. There is, however, the potential for shorter prescriptions to increase costs through increased GP workload and dispensing fees. Currently, the consequences of longer and shorter prescriptions for patients with chronic diseases are unknown and need to be assessed. The purpose of this study is to determine the if there are differences in the costs related to drug wastage, dispensing fees and prescriber time as a result of early refills and treatment switches for prescriptions issued as either a 28-day or 3-month supply in five selected case study scenarios representing common chronic conditions. This study will provide important information to the Department of Health in understanding the impact that encouraging GPs to issue shorter supplies of drugs has had on drug expenditure and drug wastage and additionally, help inform future prescribing policies.

B. Technical Summary (Max. 200 words)

Please provide a succinct overview of the objectives, methods and data analysis for the proposed research. Avoid the use of references in this section.

The aim of this study is to estimate the differences in the costs of drug wastage, dispensing fees and prescriber time as a result of early refills and treatment switches in patients receiving medications as either 28-day or 3-month supplies for a number of common chronic diseases. A retrospective cohort analysis will be conducted using data from a random sample of 50,000 patients for five case study conditions derived from all adult patients receiving at least one prescription relevant to the respective condition during the 10-year period between 2004 and 2014. The volume of wastage from early refills and treatment switches (defined as a repeat prescription or new prescription for a drug commonly prescribed for the same condition being issued prior to the expiry of the previously prescribed quantity) will be estimated. Unit costs from standard sources will be applied to estimate the cost of wastage and dispensing for a common price year. The cost of health professional time to issue the prescription will also be added. Changes in drug wastage and dispensing fees will then be estimated had all prescriptions been for 28 days rather than the observed length.

C. Objectives, Specific Aims and Rationale

Please include:

(i) The broad research objectives

(ii) The specific aims; any hypotheses to be tested should be stated here.

(iii) An explanation of how achievement of the specific aims will further the research objectives

The broad research objectives of the entire research project (note that the proposed study within this application is only one component of a larger NIHR-funded HTA project) are to assess whether shorter (28 day) or longer (3 month) prescription lengths have an impact on medication wastage, dispensing costs and health professional prescriber time.

The aims of the component of the study for which we require CPRD data are to investigate the patterns of treatment switching and early refills over a 10-year period in order to estimate differences in the cost of drug wastage, dispensing fees and health professional prescriber time for 28-day and 3-month prescription lengths.

The results of the study will provide evidence to guide policy on the optimal choice of prescription length based on the potential economic implications of different policy scenarios.

D. Background

Please provide a succinct review of the relevant background literature with references so as to explain the purpose of the study. Please ensure that you refer to any previous research in CPRD that is related, providing published references and, when known, the ISAC Protocol Number

In an effort to reduce expenditure on, and wastage of, drugs some commissioners have encouraged GPs to issue shorter prescriptions, typically 28 days in length.[NHS Cambridgeshire, 2009; NHS Dorset Clinical Commissioning Group, 2013] The rationale being, to strike a balance between patient convenience, good medical practice and drug wastage. It has been estimated that between £100 million and £300 million worth of prescriptions dispensed in the community was wasted in 2007 and 2009.[Trueman P et al., 2010] Some evidence suggests that this wastage could be reduced if prescriptions were limited to a 28 day supply.[Hawksworth GM et al., 1996]

Shorter prescriptions, however, may increase the costs to the healthcare system through increased GP workload and dispensing costs to pharmacists. Recent evidence suggested that dispensing fees, as a result of increased numbers of shorter prescriptions, cost the NHS approximately £150 million in 2009.[Wilson PM et al., 2013] If all 842.5 million prescription items dispensed in the community in England in 2008 had been 28-day repeats, dispensing fees would have been 50% higher (£700 million increase on £1.5 billion current expenditure).[White KG et al., 2010] This same conclusion followed from a simulation model published in 2004 comparing 100-day with 34-day supplies in a US Medicaid setting:[Domino ME et al., 2004] shorter prescription lengths were associated with a reduction in drug wastage of 5-14%. However, increases in dispensing fees more than exceeded this decrease in drug wastage.

Given the disparity and lack of evidence from the perspective of the NHS in the UK, it is clear that an analysis is required to assess the impact of prescription length on costs to health services in terms of wastage, dispensing fees and health professional prescriber time.

E. Study Type

Specify whether the study will be primarily descriptive, exploratory, hypothesis testing or a methodological piece of research.

Descriptive analysis.

F. Study Design

Describe the overall research design (for example, case-control, cohort) and reasons for choosing the proposed study design.

This study will be a retrospective cohort study of a random sample of 50,000 patients for each of the five case study conditions (see section K) derived from all adult (≥18 years old) patients receiving at least one prescription relevant to the respective condition (see Appendix 1) during the 10-year period between January 1, 2004 and December 31, 2014. The 10-year study period has been chosen to ensure a sufficient number of treatment switches (specifically switches between drugs that are in the same class or different classes, but therapeutically related) are observed as these may happen relatively infrequently over the course of treating some chronic diseases. Prescribing data over the 10-year period will be studied. Descriptive analyses of trends in treatment switching and early refills will be carried out for each annual period and 10-years overall.

G. Sample Size

Please provide an estimate of sample size, and, where possible, a formal power calculation. An estimate of the expected number of patients available in the CPRD database should normally be included.

A random sample of 50,000 patients for each of the five case study conditions (see section K) derived from all adult (≥18 years old) patients receiving at least one prescription relevant to the respective condition (see Appendix 1) during the 10-year period between 2004 and 2014 will be included. If we assume on average five years of follow up are available for a patient in CPRD [Herrett E et al., 2015] and that patients may be issued the prescriptions of interest for half that time (note this may be different depending on the condition of interest, but has been used as a lower limit) and that patients are likely to receive between 4 and 12 prescriptions per year (based on dispensing of either a one month or three month supply) than overall, patients in CPRD are likely to have between 10 and 30 prescriptions related to the conditions of interest. A random sample of 50,000 patients would result in roughly 500,000 to 1.5 million prescriptions in total. Given previously reported annual proportions of treatment switches for angiotensin-converting enzyme inhibitors (2.6%), sulfonylureas (0.8%) and selective serotonin reuptake inhibitors (1.0%) [Domino ME et al. 2004] and if we look to assess the number of switches each year over our 10-year study period, assuming the number of prescriptions is equally spread over the 10 years (~42,000/year) for a sample of 50,000 (lower limit 500,000 prescriptions in total) we would expect to detect these proportions of switches with acceptable precision [0.01 95% CI (0.0090705, 0.0109981) and 0.03 95% CI (0.0283892, 0.0316762)].

H. Data Linkage Required (if applicable)

Please provide a synopsis of the purpose(s) for which the each of the linkages requested in section 18 of the application form is required.

Not applicable.

I. Study Population

Define the source and study population, in terms of persons, place, time period, and listing the criteria which will be used to select the study population from the CPRD, i.e any inclusion or exclusion criteria. Please make clear any restrictions imposed by the use of linked datasets.

A random sample of 50,000 patients for each of the five case study conditions (see section K) derived from all adult (≥18 years old) patients receiving at least one prescription relevant to the respective condition (see Appendix 1) during the 10-year period between January 1, 2004 and December 31, 2014 will be included.

J. Selection of comparison group(s) or controls

Describe the criteria for eligibility and the procedure for control selection.

Not applicable.

K. Exposures, Outcomes and Covariates

For exposures and outcomes operational definitions (or procedures for developing them) must be provided, supported by preliminary code lists placed in an Annex. A comprehensive list of covariates should also be provided for any study which is not purely descriptive.

Five case study conditions were selected based on their frequency of occurrence within the population and the potential for a variety of expected frequencies in prescription changes over the course of treatment for each condition.

A list of medications routinely prescribed for the selected case study conditions was identified by review of appropriate clinical guidelines and consultation with clinical colleagues.

The five case study conditions are:

1) glucose control in type II diabetes (patients receiving at least one prescription for an anti-diabetic drug listed under 'BNF 6.1.2 Antidiabetic drugs');

2) primary prevention of hypertension in type II diabetes (in addition to receiving an anti-diabetic drug as defined in (1), patients receiving at least one prescription for a medication used for the primary prevention of hypertension in type II diabetes patients, including angiotensin-converting enzyme inhibitors, angiotensin II receptor antagonists, calcium-channel blockers and thiazide-like diuretics);

3) primary prevention of hyperlipidaemia in type II diabetes (in addition to receiving an anti-diabetic drug as defined in (1), patients receiving at least one prescription for a statin used for the primary prevention of hyperlipidaemia in type II diabetes patients;

4) secondary prevention of myocardial infraction (in addition to receiving concurrent prescriptions for a angiotensin-converting enzyme inhibitor, antiplatelet and statin for at least one year in duration, patients may also receive prescriptions for beta-adrenoceptor blockers, calcium-channel blockers, oral anticoagulants and aldosterone antagonists);

5) and depression (patients receiving at least one prescription for an anti-depressant drug listed under 'BNF 4.3 Antidepressant drugs').

Preliminary product code lists of potentially prescribed medications for each of the five case study conditions are provided in Appendix 1.

L. Data/ Statistical analysis

This section should cover both the analytic methods and also the analyses which are to be performed to meet all the specific aims listed earlier. It is important to ensure that this section is clear and specific about any comparisons which will be made.

For each of the five case study conditions the associated product codes listed in Appendix I will first be reviewed to create groups of similar products, where possible. Next, all possible substitutions between the available products will be mapped and will include:

1)substitutions between different dosages or formulations of the same drug substance (active ingredient);
2) substitutions between drugs within the same class (e.g., switch between two different statins);
3) and substitutions between drugs that are therapeutically related (e.g., switch from angiotensin-converting enzyme inhibitor to calcium-channel blocker)

We will then estimate the volume of medication wastage from early refills and treatment switches (defined as a repeat prescription or new prescription based on the mapped substitutions outlined above respectively, being issued prior to the expiry of the previously prescribed quantity). This can be estimated by dividing the total quantity entered by the GP for the prescribed product (qty) by the numeric daily dose prescribed for the event (ndd) and comparing this to the difference in the two dates associated with the events, as entered by the GP (eventdate). A threshold of one year after the initial prescription in a particular series will be used to estimate wastage for early refills (i.e., repeat prescriptions), in that any product prescribed over and above the expected quantity to be consumed within the one year time period will be considered waste. This is to account for the fact that some patients may fill their prescriptions before their existing supply is exhausted, but still consume all of the previous prescription before using the new supply. Therefore, if we assumed wastage for all early refills we may be overestimating the impact.

In contrast, for treatment switches we will assume any additional product not consumed before the switch date will be considered waste. There are, however, two exceptions: 1) prescriptions issued on the same day for drugs in the same class (e.g., two different statins) will be considered prescriber error and drop from the analysis as it is unlikely that prescriptions for drugs in the same class would be issued on the same day; and 2) to differentiate between add-ons and switches, particularly for therapeutically related drugs we will only define an overlap of prescriptions dates as wastage due to a treatment switch if there is not another prescription issued for the original product within a three month time period. The three month threshold has been chosen to ensure prescriptions issued for both one and three month periods are captured. The three month threshold will also be altered in sensitivity analysis to test the robustness of this assumption.

Alternatively, we may chose not to differentiate between actual medication wastage due to switches and the augmentation of medication through add-ons as well as count any overlapping dates as wastage for early refills. Under this scenario we may overstate the amount of wastage that occurred, but this can be considered a conservative assumption, as it puts an upper bound on the savings that would occur if premature medication switches could be eliminated entirely.

The cost of wastage can then be estimated by applying net ingredient costs (NICs) obtained from national general practice prescribing data provided by the Health & Social Care Information Centre for the respective prescription to the estimated quantity of waste. BNF codes will be used to link the NICs from the general prescribing data to CPRD. Since BNF codes from CPRD are limited to only a 7 digit numeric code representing chapter, section, paragraph and subparagraph (i.e., does not provide 8 digit alpha/numeric code representing the drug substance and specific formulation) it is necessary to make some additional assumptions to link the datasets. First, total NIC will be divided by total number of items prescribed and dispensed for each product listed to obtain a NIC per item. A weighted average using total quantities of all the NICs per item for a particular BNF paragraph will be calculated. For example, BNF code 0403010 in CPRD represents Chapter 4 “Central nervous system”, Section 3 “Antidepressant drugs”, Paragraph 1 “Tricyclic and related antidepressant drugs” and Subparagraph 0, which means the BNF does not extend to the subparagraph level in this case. Therefore a weighted average of all the NICs in Paragraph 1 “Tricyclic and related antidepressant drugs” will be applied to any drug falling in this category (e.g., amitriptyline, clomipramine, dosulepin, doxepin, imipramine, etc.).

Based on the number of prescriptions, dispensing fees related to each prescription from the Drug Tariff and the estimated cost of health professional prescriber time based on the literature can be added to the cost of wastage to determine the total cost from a NHS perspective. A targeted literature review will be designed to determine the time involved for a health professional to issue a prescription. Note this may be different depending on the type of health professional (e.g., general practitioner versus nurse), but this will be tested in sensitivity analysis. Hourly costs related to the health professionals’ time, derived from the PSSRU’s Unit Costs of Health & Social Care will then be applied.[Curtis L and Burns A, 2015]

Scenario analysis will then be conducted, estimating changes in drug wastage and dispensing fees if all prescriptions had been for 28 days rather than the observed length. Appropriate sensitivity analyses will also be conducted, for example, around the cost of health professional prescriber time required to issue a repeat prescription.

M. Plan for addressing confounding

Purely descriptive studies are exempt from this requirement. All other studies should here provide some discussion of what they are doing in the design and/or analysis to control for confounding.

Not applicable.

N. Plan for addressing missing data

The potential for missing data should be identified and how it will be addressed discussed here.

Missing data in the CPRD therapy file should not be a major issue. Our analysis does, however, rely on the use of the numeric daily dose (ndd) variable. As this variable is derived using a CPRD algorithm on common dosage strings there is the potential for it to be equal to zero in cases of a non-numeric textid (e.g., if the textid refers to say “apply as needed”). This type of textid is unlikely for the medications chosen to be included in our analysis and therefore our analysis will be limited to only those observations with a complete case (i.e., ndd is not missing or equal to zero and quantity is not missing). This seems to be acceptable as this approach has been employed in other similar CPRD studies using these two variables.[Brodie MJ et al., 2016 and Francis NA et al., 2016]

O. Limitations of the study design, data sources and analytical methods

The general limitations of the databases and observational research are well-known. Specific consideration of the potential impact of such limitations should be provided in the context of the proposed study.

The key limitations specific to this protocol are as follows:

1. To define three of the five case study conditions (see section K; conditions 2, 3 and 4) based only on the available prescription data from CPRD it was necessary to make assumptions regarding the population’s composition. For example, to define a population receiving medication for the secondary prevention myocardial infraction it was necessary to assume (based on clinical guidelines) that patients receiving a angiotensin-converting enzyme inhibitor, antiplatelet and statin for at least one year in duration had previously had a myocardial infraction.
2. From the data we will not be able to differentiate between repeat prescriptions and a number of acute prescriptions. Therefore to avoid overestimating wastage we have proposed to use a threshold of one year after the initial prescription in a particular series to estimate wastage for early refills (i.e., repeat prescriptions), in that any product prescribed over and above the expected quantity to be consumed within the one year time period will be considered waste.
3. Five case study conditions were purposively rather than randomly selected to represent the impact of medication refill and switching behaviour, but they may not be representative of prescribing behaviour in other chronic conditions. The conditions do, however, represent some of the most common chronic conditions treated with prescribed medications.
4. Our estimates of drug wastage will not account for imperfect adherence and therefore might represent an underestimate of the true quantity and cost of medication wastage. However, an additional aspect of this project (being conducted by other colleagues under the same NIHR HTA proposal) will attempt to quantify the impact of imperfect adherence for both long and short prescription lengths.
5. NICs used to estimate the cost of wastage are the prices listed on the Drug Tariff or if not on the tariff, the list prices published by the manufacturer. NICs do not include any discounts that may be applied. NICs also do not include any adjustment for income obtained where a prescription charge is paid at the time the prescription is dispensed or where the patient has purchased a pre-payment certificate. However, NICs are the only linkable source of prescription drug unit for large datasets like CPRD.
6. BNF codes will be used to link the NICs from the general prescribing data to CPRD. Since BNF codes from CPRD are limited to only a 7 digit numeric code representing chapter, section, paragraph and subparagraph (i.e., does not provide 8 digit alpha/numeric code representing the drug substance and specific formulation) it is

necessary to make some additional assumptions to link the datasets. First, total NIC will be divided by total number of items prescribed and dispensed for each product listed to obtain a NIC per item. A weighted average using total quantities of all the NICs per item for a particular BNF subparagraph will be calculated.

7. A main limitation of CPRD prescription data is that it does not indicate whether or not a medication has been dispensed or whether patients took their prescribed medications as recommended (i.e., it only indicate when a prescription has been issued). Therefore, our estimates may have overstated the amount of wastage that actually occurred. This, however, is a conservative assumption and can be seen as an upper bound on the savings that would occur if drug wastage from premature medication switches could be eliminated entirely.

P. Patient or user group involvement (if applicable)

Please indicate whether you have or intend to involve patient groups in your study. Such involvement is encouraged by ISAC and required for studies which directly involve patients.

In preparation for this proposal, we sent an outline of our proposed research to members of INsPIRE, a patient and public involvement panel for Bedfordshire and Cambridgeshire. Email comments were sent back from seven panel members. Five of the members stressed the importance of this research and six members maintained that three month prescriptions were preferable to 28 day prescriptions for chronic conditions. However, one member cautioned that 28 day prescriptions may be suitable for ‘concern medications’, such as sleeping pills. Six of the respondents mentioned the additional cost of shorter duration prescriptions, which they described in terms of drug wastage, patient time, GP time and prescription fees. Two members also mentioned the importance of synchronisation of prescriptions for patients with multiple co-morbidities. Finally, two members stressed the importance of focusing on individual patient needs when prescribing medications. During the writing of the proposal, we have taken these views into account.

Patients and the public were involved in the design of the research and will be involved in the dissemination of research findings.

Q. Plans for disseminating and communicating study results, including the presence or absence of any restrictions on the extent and timing of publication

ISAC expects most studies that it approves to be published in the scientific literature and considers it an ethical obligation for any study with potential public health implications. In cases where multiple publications are likely to arise, a publication plan should be provided in this section.

The primary audience for the proposed research will be policy makers, those who manage and provide care for patients with long-term stable chronic conditions (i.e., general practitioners and pharmacists), as well as patient groups with stable, chronic conditions who require regular repeat prescriptions. In addition to a HTA monograph, we plan to publish the findings in an academic peer-reviewed journal and present the findings at relevant academic conferences. Our patients and public involvement members will be asked to assist in the production of a short summary for non-technical audiences.

R. References

Please provide a numbered list of references at the end of the protocol.

Brodie MJ, Chung S, Wade A, Quelen C, Guiraud-Diawara A, François C, Verpillat P, Shen V, Isojarvi Clobazam and clonazepam use in epilepsy: Results from a UK database incident user cohort study. Epilepsy Research 2016;123:68-74.

Curtis L, Burns A. Personal Social Services Research Unit – Unit Costs of Health & Social Care 2015. The University of Kent. Canterbury.

Domino ME, Olinick J, Sleath B, Leinwand S, Byrns PJ, Carey T. Restricting patients' medication supply to one month: Saving or wasting money? Am J Health-Syst Pharm 2004;61:1375-1379.

Francis NA, Hood K, Lyons R, Butler CC. Understanding flucloxacillin prescribing trends and treatment non-response in UK primary care: a Clinical Practice Research Datalink (CPRD) study. J Antimicrob Chemother 2016 Apr 18.

Hawksworth GM, Wright D, Chrystyn H. A detailed analysis of the day to day unwanted medicinal products returned to community pharmacies for disposal. Journal of social and administrative pharmacy 1996;13:215-222.

Herrett E, Gallagher AM, Bhaskaran K, Forbes H, mathur R, van Staa T, Smeeth L. Data Resource Profile: Clinical Practice Research Datalink (CPRD). International Journal of Epidemiology 2015;44(3):827-836.

NHS Cambridgeshire. Repeat Medication for 28 Days. 2009

NHS Dorset Clinical Commissioning Group. Medicines Code Chapter 15: Policy for Repeat Prescribing and Medication Review. 2013

Trueman P, Lowson K, Blighe A et al. Evaluation of the Scale, Causes and Costs of Waste Medicines. York & London: York Health Economics Consortium & The School of Pharmacy, University of London, 2010.

White KG. UK interventions to control medicines wastage: a critical review. Int J Pharm Pract 2010;18(3):131-40.

Wilson PM, Kataria N, McNeilly E. patient and carer experience of obtaining regular prescribed medication for chronic disease in the English National Health Service: a qualitative study. BMC health services research 2013;13(1):192.

Appendices

Appendices should be used for essential supporting information only (e.g. code-lists) and they must be cited within the body of the protocol.

Please see accompanying document:

- Appendix 1: Preliminary product codes lists for each of the five case study conditions of interest (Excel file)

Appendix VIII - Comparison of medication wastage over 11-year period 2004-2014

	Proportion of days' supply wasted % (95% CI)		Mean number of days' supply wasted days (95% CI)		Mean cost of wastage per prescription 2015 £ (95% CI)	
	**<60 days**	**≥60 days**	**<60 days**	**≥60 days**	**<60 days**	**≥60 days**
Glucose control with oral drug therapy in T2DM	2.658 (2.641-2.675	4.920 (4.822-5.018)	0.859 (0.853-0.865)	4.962 (4.044-5.880)	0.329 (0.325-0.333)	1.370 (1.104-1.636)
Hypertension in T2DM	3.762 (3.747-3.777)	5.011 (4.935-5.087)	1.232 (1.227-1.237)	6.979 (5.956-8.002)	0.095 (0.094-0.096)	0.437 (0.389-0.486)
Lipid management in T2DM	1.652 (2.640-1.665)	4.071 (3.966-4.177)	0.628 (0.623-0.633)	16.211 (12.979-19.443)	0.048 (0.048-0.049)	1.426 (1.132-1.720)
Secondary prevention of myocardial infraction	3.325 (3.315-3.335)	3.663 (3.612-3.714)	0.956 (0.953-0.959)	6.557 (5.761-7.353)	0.066 (0.066-0.066)	0.510 (0.370-0.649)
Depression	6.340 (6.157-6.385)	3.663 (3.535-3.792)	1.866 (1.852-1.881)	2.695 (2.592-2.797)	0.207 (0.203-0.212)	0.429 (0.977-0.480)

CI – confidence interval; T2DM – type II diabetes mellitus

Appendix IX - Comparison of the mean cost of medication wastage per prescription each year from 2004 to 2014 (2015 £)

	**Glucose control with oral drug therapy in T2DM 2015 £ (95% CI)**		**Hypertension in T2DM 2015 £ (95% CI)**		**Lipid management in T2DM 2015 £ (95% CI)**		**Secondary prevention of myocardial infraction 2015 £ (95% CI)**		**Depression 2015 £ (95% CI)**	
**Year**	**<60 days**	**≥60 days**	**<60 days**	**≥60 days**	**<60 days**	**≥60 days**	**<60 days**	**≥60 days**	**<60 days**	**≥60 days**
2004	0.381 (0.364-0.397)	1.813 (0.574-3.052)	0.127 (0.122-0.131)	0.462 (0.351-0.573)	0.068 (0.065-0.070)	1.511 (0.599-2.422)	0.085 (0.084-0.087)	0.376 (0.175-0.578)	0.247 (0.227-0.266)	0.428 (0.308-0.548)
2005	0.423 (0.405-0.441)	1.331 (1.080-1.582)	0.123 (0.118-0.127)	0.525 (0.386-0.663)	0.055 (0.053-0.058)	2.363 (1.118-3.607)	0.082 (0.081-0.084)	0.521 (0.321-0.721)	0.221 (0.204-0.238)	0.452 (0.296-0.608)
2006	0.418 (0.400-0.435)	1.045 (0.906-1.185)	0.111 (0.107-0.114)	0.428 (0.328-0.529)	0.057 (0.055-0.060)	2.274 (1.059-3.488)	0.079 (0.077-0.080)	1.277 (0.132-2.421)	0.222 (0.205-0.239)	0.579 (0.359-0.800)
2007	0.387 (0.370-0.403)	1.110 (0.956-1.264)	0.101 (0.098-0.104)	0.467 (0.325-0.608)	0.050 (0.048-0.052)	1.533 (0.557-2.509)	0.072 (0.070-0.073)	0.400 (0.0240-0.559)	0.222 (0.205-0.239)	0.376 (0.247-0.505)
2008	0.343 (0.329-0.357)	1.086 (0.922-1.249)	0.096 (0.093-0.099)	0.530 (0.314-0.746)	0.045 (0.043-0.048)	1.663 (0.600-2.726)	0.065 (0.065-0.067)	0.457 (0.259-0.654)	0.207 (0.192-0.221)	0.359 (0.227-0.491)
2009	0.299 (0.287-0.311)	1.074 (0.849-1.298)	0.091 (0.088-0.093)	0.472 (0.282-0.662)	0.048 (0.046-0.051)	1.717 (0.638-2.796)	0.063 (0.062-0.064)	0.528 (0.302-0.754)	0.206 (0.191-0.220)	0.421 (0.243-0.599)
2010	0.330 (0.317-0.342)	1.396 (0.744-2.048)	0.085 (0.082-0.087)	0.379 (0.213-0.545)	0.046 (0.043-0.048)	0.991 (0.245-1.738)	0.061 (0.060-0.062)	0.477 (0.253-0.701)	0.183 (0.171-0.195)	0.366 (0.216-0.516)
2011	0.263 (0.254-0.272)	3.139 (0.659-5.620)	0.082 (0.080-0.085)	0.477 (0.259-0.695)	0.042 (0.040-0.044)	0.879 (0.154-1.605)	0.057 (0.056-0.058)	0.409 (0.208-0.611)	0.173 (0.162-0.183)	0.383 (0.239-0.528)
2012	0.251 (0.243-0.260)	0.932 (0.820-1.043)	0.078 (0.075-0.080)	0.402 (0.208-0.596)	0.045 (0.043-0.047)	0.740 (0.135-1.346)	0.056 (0.055-0.057)	0.354 (0.192-0.516)	0.182 (0.169-0.195)	0.350 (0.171-0.528)
2013	0.268 (0.258-0.277)	0.871 (0.758-0.984)	0.074 (0.072-0.076)	0.232 (0.189-0.275)	0.038 (0.036-0.039)	0.448 (0.063-0.834)	0.053 (0.052-0.054)	0.200 (0.131-0.268)	0.199 (0.186-0.213)	0.436 (0.189-0.683)
2014	0.301 (0.290-0.311)	1.168 (1.018-1.318)	0.079 (0.076-0.082)	0.271 (0.202-0.340)	0.047 (0.045-0.050)	0.796 (0.102-1.489)	0.054 (0.053-0.056)	0.188 (0.103-0.273)	0.233 (0.217-0.249)	0.593 (0.374-0.812)

NS – not significant at p<0.05 level; T2DM – type II diabetes mellitus

Appendix X – Differences in standardised (120 day) total unnecessary costs for short and long prescription length under various scenarios (2015 £)

	**Values tested**		**Total unnecessary cost standardised to 120 days**		**Difference (Cost savings with ≥60 day)**
Scenarios	**<60 days**	**≥60 days**	**<60 days**	**≥60 days**	
**Initial glucose control in type II diabetes**					
Base case	-	-	13.24	4.86	(8.38)
Nurse prescriber time cost, not GP	2.28	2.29	10.13	4.19	(5.94)
No prescriber time cost	0	0	3.76	2.85	(0.91)
Addition of prescription charge (loss in NHS revenue)	0.84	0.84	10.89	4.37	(6.52)
50% decrease quantity wasted, days	0.43	2.48	12.65	3.74	(8.91)
50% increase quantity wasted, days	1.29	7.44	13.84	5.98	(7.86)
50% decreased cost of drug per day	0.18	0.14	12.65	3.74	(8.91)
50% increase cost of drug per day	0.55	0.43	13.84	5.98	(7.86)
50% decrease dispensing fee	0.46	0.52	11.96	4.55	(7.41)
50% increase dispensing fee	1.38	1.57	14.53	5.16	(9.37)
50% decrease prescriber time cost	1.70	1.72	8.50	3.85	(4.65)
50% increase prescriber time cost	5.09	5.16	17.99	5.86	(12.13)
**Hypertension in type II diabetes**					
Base case	-	-	12.32	2.50	(9.82)
Nurse prescriber time cost, not GP	2.31	2.32	9.04	2.03	(7.01)
No prescriber time cost	0	0	2.81	1.15	(1.66)
Addition of prescription charge (loss in NHS revenue)	0.84	0.84	10.06	2.18	(7.88)
50% decrease quantity wasted, days	0.62	3.49	12.13	2.10	(10.03)
50% increase quantity wasted, days	1.85	10.47	12.52	2.89	(9.63)
50% decreased cost of drug per day	0.042	0.041	12.13	2.10	(10.03)

	Values tested		Total unnecessary cost standardised to 120 days		Difference (Cost savings with ≥60 day)
Scenarios	**<60 days**	**≥60 days**	**<60 days**	**≥60 days**	
50% increase cost of drug per day	0.13	0.12	12.52	2.89	(9.63)
50% decrease dispensing fee	0.45	0.46	11.11	2.32	(8.79)
50% increase dispensing fee	1.35	1.39	13.54	2.67	(10.87)
50% decrease prescriber time cost	1.77	1.77	7.57	1.82	(5.75)
50% increase prescriber time cost	5.30	5.32	17.08	3.17	(13.91)
**Hyperlipidaemia in type II diabetes**					
Base case	-	-	10.95	1.54	(9.41)
Nurse prescriber time cost, not GP	2.21	2.22	8.54	1.56	(6.98)
No prescriber time cost	0	0	2.64	1.61	(1.03)
Addition of prescription charge (loss in NHS revenue)	0.84	0.84	8.71	1.56	(7.15)
50% decrease quantity wasted, days	0.31	8.11	10.83	0.73	(10.10)
50% increase quantity wasted, days	0.94	24.32	11.07	2.36	(8.71)
50% decreased cost of drug per day	0.052	0.052	10.83	0.73	(10.10)
50% increase cost of drug per day	0.16	0.15	11.07	2.36	(8.71)
50% decrease dispensing fee	0.45	0.50	9.75	1.55	(8.20)
50% increase dispensing fee	1.35	1.50	12.15	1.53	(10.62)
50% decrease prescriber time cost	1.56	1.57	6.79	1.58	(5.21)
50% increase prescriber time cost	4.68	4.72	15.11	1.51	(13.60)
**Secondary prevention of myocardial infraction**					
Base case	-	-	13.40	1.34	(12.06)
Nurse prescriber time cost, not GP	2.37	2.37	9.49	1.10	(8.39)
No prescriber time cost	0	0	2.81	0.69	(2.12)
Addition of prescription charge (loss in NHS revenue)	0.84	0.84	11.03	1.20	(9.83)

	Values tested		Total unnecessary cost standardised to 120 days		Difference (Cost savings with ≥60 day)
Scenarios	**<60 days**	**≥60 days**	**<60 days**	**≥60 days**	
50% decrease quantity wasted, days	0.48	3.28	13.27	1.08	(12.19)
50% increase quantity wasted, days	1.43	9.84	13.54	1.60	(11.94)
50% decreased cost of drug per day	0.037	0.034	13.27	1.08	(12.19)
50% increase cost of drug per day	0.11	0.10	13.54	1.60	(11.94)
50% decrease dispensing fee	0.45	0.48	12.13	1.26	(10.87)
50% increase dispensing fee	1.35	1.45	14.67	1.43	(13.24)
50% decrease prescriber time cost	1.88	1.89	8.11	1.02	(7.09)
50% increase prescriber time cost	5.63	5.66	18.70	1.67	(17.03)
**Depression**					
Base case	-	-	15.06	4.04	(11.02)
Nurse prescriber time cost, not GP	2.24	2.23	11.72	3.24	(8.48)
No prescriber time cost	0	0	4.16	1.35	(2.81)
Addition of prescription charge (loss in NHS revenue)	0.84	0.84	12.22	3.33	(8.89)
50% decrease quantity wasted, days	0.93	1.35	14.51	3.77	(10.74)
50% increase quantity wasted, days	2.80	4.04	15.61	4.31	(11.30)
50% decreased cost of drug per day	0.068	0.054	14.51	3.77	(10.74)
50% increase cost of drug per day	0.20	0.16	15.61	4.31	(11.30)
50% decrease dispensing fee	0.45	0.48	13.53	3.64	(9.89)
50% increase dispensing fee	1.36	1.44	16.59	4.45	(12.14)
50% decrease prescriber time cost	1.61	1.59	9.61	2.70	(6.91)
50% increase prescriber time cost	4.84	4.78	20.51	5.39	(15.12)

REFERENCES FOR APPENDICIES

1. Taitel M, Fensterheim L, Kirkham H, et al. Medication Days' Supply, Adherence, Wastage, and Cost Among Chronic Patients in Medicaid. Medicare & Medicaid Research Review 2012;**2**(3):E1-E13.

2. National Institute for Health and Care Excellence (NICE). *Type 2 diabetes in adults: management (NG28)*. London, UK: NICE, December 2015.

3. National Institute for Health and Care Excellence (NICE). British National Formulary (BNF). London, UK: NICE, 2016.

4. National Institute for Health and Care Excellence (NICE). *Myocardial infraction: cardiac rehabilitation and prevention of further MI (CG172)*. London, UK: NICE, November 2013.

5. Hollingworth W, Devine EB, Hansen RN, et al. The impact of e-prescribing on prescriber and staff time in ambulatory care clinics: a time motion study. J Am Med Inform Assoc 2007;**14**(6):722-30.

6. Westbrook JI, Li L, Georgiou A, et al. Impact of an electronic medication management system on hospital doctors' and nurses' work: a controlled pre-post, time and motion study. J Am Med Inform Assoc 2013;**20**(6):1150-8.

7. Franklin BD, O'Grady K, Donyai P, et al. The impact of a closed-loop electronic prescribing and administration system on prescribing errors, administration errors and staff time: a before-and-after study. Qual Saf Health Care 2007;**16**(4):279-84.

8. Laurant M, Reeves D, Hermens R, et al. Substitution of doctors by nurses in primary care. Cochrane Database Syst Rev 2005(2):CD001271.

9. Shum C, Humphreys A, Wheeler D, et al. Nurse management of patients with minor illnesses in general practice: multicentre, randomised controlled trial. BMJ 2000;**320**(7241):1038-43.

10. Venning P, Durie A, Roland M, et al. Randomised controlled trial comparing cost effectiveness of general practitioners and nurse practitioners in primary care. BMJ 2000;**320**(7241):1048-53.
