# Supplementary material for: Retrospective, multicohort analysis of the Clinical Practice Research Datalink (CPRD) to determine differences in the cost of medication wastage, dispensing fees and prescriber time of issuing either short (<60 days) or long (≥60 days) prescription lengths in primary care for common, chronic conditions in the UK
Source: BMJ Open. 2017 Dec 4;7(12):e019382. doi: 10.1136/bmjopen-2017-019382 (PMC5719293; doi:10.1136/bmjopen-2017-019382)
Supplement: Author's manuscript [file bmjopen-2017-019382.draft_revisions.pdf]

BMJ Open is committed to open peer review. As part of this commitment we make the peer review history of every article we publish publicly available.

When an article is published we post the peer reviewers' comments and the authors' responses online. We also post the versions of the paper that were used during peer review. These are the versions that the peer review comments apply to.

The versions of the paper that follow are the versions that were submitted during the peer review process. They are not the versions of record or the final published versions. They should not be cited or distributed as the published version of this manuscript.

BMJ Open is an open access journal and the full, final, typeset and author-corrected version of record of the manuscript is available on our site with no access controls, subscription charges or pay-per-view fees (http://bmjopen.bmj.com).

If you have any questions on BMJ Open's open peer review process please email editorial.bmjopen@bmj.com

BMJ Open

Short versus long prescription lengths: a retrospective analysis of the Clinical Practice Research Datalink (CPRD) to determine differences in the cost of medication wastage, dispensing fees and prescriber time

Journal:	*BMJ Open*
Manuscript ID	bmjopen-2017-019382
Article Type:	Research
Date Submitted by the Author:	30-Aug-2017
Complete List of Authors:	Doble, Brett<br>Payne, Rupert; University of Bristol, Centre for Academic Primary Care<br>Harshfield, Amelia<br>Wilson, Edward
**Primary Subject Heading**:	Health economics
Secondary Subject Heading:	General practice / Family practice, Health services research, Health policy, Evidence based practice
Keywords:	PRIMARY CARE, Health policy < HEALTH SERVICES ADMINISTRATION & MANAGEMENT, HEALTH ECONOMICS, THERAPEUTICS

SCHOLARONE™
Manuscripts

For peer review only

For peer review only - http://bmjopen.bmj.com/site/about/guidelines.xhtml

Short versus long prescription lengths: a retrospective analysis of the Clinical Practice Research Datalink (CPRD) to determine differences in the cost of medication wastage, dispensing fees and prescriber time

Brett Doble*[1,2], Rupert Payne[1,3], Amelia Harshfield[1,4], Edward C.F. Wilson[1]

[1]Cambridge Centre for Health Services Research, University of Cambridge, Cambridge, CB2 0SR, United Kingdom

[2]Health Economics Research Centre, Nuffield Department of Population Health, University of Oxford, Oxford, OX3 7LF, United Kingdom

[3]Centre for Academic Primary Care, Department of Population Health Sciences, Bristol Medical School, University of Bristol, Bristol, BS8 2PS, United Kingdom

[4]Department of Public Health and Primary Care, University of Cambridge, Cambridge, CB1 8RN, United Kingdom

*Brett Doble, Health Economics Research Centre, Nuffield Department of Population Health, University of Oxford, Oxford, OX3 7LF, United Kingdom. Tel: +44(0)1865617913; Email: brett.doble@dph.ox.ac.uk

Funding Sources: This research was supported by a grant from the National Institute for Health Research, Health Technology Assessment funding stream (Grant Reference: NIHR HTA 14/159/07). This article presents independent research funded by the National Institute for Health Research (NIHR). The views expressed are those of the authors and not necessarily those of the National Health Service, the National Institute for Health Research or the Department of Health.

Acknowledgements: This study is based on data from the Clinical Practice Research Datalink obtained under license from the UK Medicines and Healthcare Products Regulatory Agency. The interpretation and conclusions contained in this study are those of the authors alone. We would also like acknowledge the valuable feedback received from Céline Miani, Adam Martin, Josephine Exley, Catherine Meads and Sarah King during the conduct of this study.

Word Count: 3,852

For peer review only - http://bmjopen.bmj.com/site/about/guidelines.xhtml

ABSTRACT

Objectives: To investigate patterns of early refills and treatment switching over an 11-year period to estimate differences in the cost of medication wastage, dispensing fees and prescriber time for short (<60 days) and long (≥60 days) prescription lengths from the perspective of the National Health Service in the United Kingdom.

Setting: Retrospective, multiple cohort study of primary care prescriptions from the Clinical Practice Research Datalink.

Participants: Five random samples of 50,000 patients each prescribed oral drugs for (1) glucose control in type 2 diabetes mellitus (T2DM), (2) hypertension in T2DM, (3) statins (lipid management) in T2DM, (4) secondary prevention of myocardial infarction and (5) depression.

Primary and secondary outcome measures: The volume of medication wastage from early refills and three other types of treatment switches was quantified and costed. Dispensing fees and prescriber time were also determined. Total unnecessary costs (TUC, cost of medication wastage, dispensing fees, and prescriber time) associated with <60 day and ≥60 day prescriptions, standardised to a 120-day period, were then compared.

Results: Longer prescription lengths were associated with more medication waste per prescription. However, when including dispensing fees and prescriber time, longer prescription lengths resulted in lower TUC. This finding was consistent across all five cohorts. Savings ranged from £8.38 to £12.06 per prescription per 120 days if a single long prescription were issued instead of multiple short prescriptions. Prescriber time costs accounted for the largest component of TUC**.**

Conclusions: Shorter prescription lengths could potentially reduce medication wastage, but they may also increase dispensing fees and/or the time burden of issuing prescriptions.

Keywords: costs, fill quantity, medication wastage, prescribing policy, prescription length

For peer review only - http://bmjopen.bmj.com/site/about/guidelines.xhtml

ARTICLE SUMMARY

Strengths and limitations of this study

- Our analysis builds on existing methodological approaches to estimate the unnecessary costs associated with different prescription lengths, providing the only evidence available from the perspective of the NHS in the UK.
- Limitations of our study do risk biasing the results and the reported savings (£8.38 to £12.06 per prescription per 120 days) should therefore be interpreted with caution and considered upper limits.
- CPRD prescription data only indicates whether a prescription has been issued and not whether it was dispensed or taken as recommended, potentially resulting in an over- or under-estimate of the amount of wastage, depending on patient behaviour not captured in CPRD.
- The five case study conditions used in our study were purposively rather than randomly selected to represent the impact of medication refill and switching behaviour on wastage; they may not be representative of prescribing behaviour in other chronic conditions.
- Overlap of dates between prescriptions does not necessarily mean wastage has occurred and despite incorporating methods to account for this there is the possibility that our analysis approach could be overestimating the amount of medication wastage.

For peer review only - http://bmjopen.bmj.com/site/about/guidelines.xhtml

INTRODUCTION

Healthcare systems worldwide are increasingly faced with the challenge of constraining rising pharmaceutical expenditures.[1] One approach to addressing this problem is to ensure prescribed medication is used as efficiently as possible, minimising wastage. Wastage may occur when patients refill their scripts early, or when changes are made to patients' drug regimens. Intuitively, the more drugs a patient has in her/his possession at the time of a refill or regimen change, the higher the wastage. Therefore, limiting the quantity of medication through shorter prescription lengths could minimise wastage and help contain expenditure. However, the resulting higher frequency of prescriptions will have the unintended consequence of increasing transactions costs, specifically dispensing fees charged by pharmacists and healthcare professionals' time to issue them.

Several studies have examined the costs associated with issuing either long (three-month) or short (one-month) supplies of prescriptions.[2-7] In general, these concluded that shorter prescriptions were associated with lower wastage and hence reduced cost, but the increased transactions costs of shorter prescriptions more than offset these savings. These studies are all US based, which has very different healthcare systems from the UK, particularly with regards to the cost and dispensing of drugs. Therefore, the generalisability of these conclusions to the UK are questionable. Furthermore, none of the studies include healthcare professionals' time burden associated with issuing prescriptions.

In this study we estimate differences in the costs of medication wastage and transactions costs (in terms of dispensing fees and prescriber time) in patients receiving medications within the NHS in the UK as either short or long prescription lengths for five drugs/classes of drugs prescribed in primary care for common, chronic conditions.

For peer review only - http://bmjopen.bmj.com/site/about/guidelines.xhtml

METHODS

Overview

We undertook an analysis of Clinical Practice Research Datalink (CPRD)[8] prescription data to estimate the cost of medication wastage associated with shorter and longer prescription lengths for drugs used to treat five case study conditions. In order to estimate the net cost impact of shorter and longer prescription lengths, the cost of dispensing fees and prescribers' time to issue a prescription were also assessed.

Study design and inclusion criteria

This retrospective multi-cohort study evaluated medication wastage and its associated cost plus dispensing fees and the cost associated with issuing a prescription (i.e., a general practitioner (GP) completing the process of producing a prescription; note this does not include clinical decision-making time or administrative staff time) in five, condition-specific, random samples of 50,000 patients each, obtained from CPRD.

We derived the five samples from all adult patients (≥18 years old) receiving one or more prescriptions for at least one medication relevant to a case study of interest (Table 1) between January 1, 2004 and December 31, 2014. In line with other studies of CPRD data [9 10] inclusion was restricted to patients with complete data for two variables (numeric daily dose [ndd] and quantity [qty]) required to calculate the prescription duration. The five case studies were defined using unique lists of product codes (CPRD unique code for treatment selected by the GP). They were: 1) glucose control with oral drug therapy in type II diabetes mellitus (T2DM); 2) treatment of hypertension in T2DM; 3) treatment with statins (lipid management) in T2DM; 4) treatment for the secondary prevention of myocardial infarction (MI); and 5) treatment of depression.

For peer review only - http://bmjopen.bmj.com/site/about/guidelines.xhtml

These were selected for study based on the chronic nature and prevalence of the associated condition within the population, and the potential for a variety of prescription changes over the course of treatment. Definitions of the relevant prescriptions and product code lists of the potentially prescribed medications for each of the five case studies are provided in Table 1 and Appendix I respectively. Sample data counts are provided in Appendix II.

Treatment patterns evaluated

For each cohort, data for each patient were first ordered in sequence from earliest to latest prescription date. To identify treatment patterns three main variables were used: 1) product code (used to identify a unique dosage, formulation and brand (or generic version) of one particular drug); 2) drug substance (used to identify different dosages and/or formulations of the same drug chemical substance); and 3) drug class (used to identify drugs with different, but related chemical composition, with similar mechanisms of action based on their categorisation in the British National Formulary (BNF)). Four different prescription patterns in an individual's sequence of prescriptions were identified: 1) refills of the same product code; 2) substitutions between different dosages or formulations of the same drug substance; 3) substitutions between drugs that are in the same class; and 4) substitutions between drugs that have similar clinical indications from different classes. Prescriptions issued on the same day for drugs in the same class with different product codes were considered prescriber error and the duplicates were dropped from the analysis. The exception to this was for antiplatelet drugs in secondary prevention of MI, as it was assumed that two different antiplatelet drugs could be prescribed at the same time. In addition, prescriptions for medications with similar clinical indications from different classes issued on the same day were not counted as a switch, but rather as an add-on to existing therapy or concomitant therapy (Appendix II).

Analysis of wastage

For peer review only - http://bmjopen.bmj.com/site/about/guidelines.xhtml

Wastage from early refills (pattern 1) was based on a cumulative excess supply built up over a period of 1 year. This avoided overestimation of wastage where a patient filled a prescription a few days early, but then finished their previous supply before starting the new one. In estimating wastage from switches (patterns 2-4), we adapted a previous approach [6] to differentiate between add-ons/concomitant therapy and actual switches. If the difference between the number of changes between medications with similar clinical indications from different classes and the number of unique drug classes within a rolling annual period was ≥1, then any overlap in prescription dates were considered to be an add-on rather than a switch. This is illustrated in Box 1. Similar constraints were also applied in three of the case studies (i.e., the glucose control in T2DM, treatment of hypertension in T2DM and secondary prevention of myocardial infraction cohorts) due to the potential for a number of the included therapies to be given concomitantly (Appendix III).

Costs

To estimate the costs of wastage, defined daily doses (DDD) associated with each drug substance code in the five cohorts were first obtained from the World Health Organisation's ATC/DDD Index 2016.[11] The Prescription Cost Analysis (PCA) 2015 which provides details of the quantity of individual doses and net ingredient costs (NICs) of all the prescriptions in England [12] was used to determine a NIC/quantity value of a specific strength of the medication associated with each drug substance code. This value was standardised using the associated DDD to obtain a cost per day for each drug substance code in all five of the cohorts. Details of these calculations are provided in Appendix IV.

Dispensing fees from the Drug Tariff (£0.90 per standard prescription and 2% of the cost per prescription [cost per day multiplied by prescription length] for prescriptions over

For peer review only - http://bmjopen.bmj.com/site/about/guidelines.xhtml

£100) [13] and the estimated cost of physician or nurse time to issue a prescription were determined for each prescription. Time to issue a prescription was extracted from a targeted literature review (Appendix V). Refills were assigned a shorter time compared to changes in dose/formulation, within drug classes and between drug classes (48.7 versus 61.2 seconds).[14] Per minute costs related to GPs' time (£3.80/minute) or a general practice nurse's time (£0.93/minute) were then applied.[15] All costs are reported in 2015 GBP.

Statistical analysis

Descriptive analyses of trends in treatment switching and early refills were used to assess medication wastage. The proportion of days' supply wasted, mean number of days' supply wasted and the mean costs of wastage per prescription were determined for two prescription lengths (<60 and ≥60 days, hereafter 'short' and 'long' prescriptions) over the 11-year period. Mean cost of wastage per prescription was reported for each of the four treatment patterns individually and for all treatment patterns combined for each annual period. Two-sample *t*-tests using groups (<60 and ≥60 days prescription lengths) assuming unequal variance were used to compare the differences between the <60 days and ≥60 days groups.

To determine and compare the total unnecessary costs (TUC, cost of medication wastage, dispensing fees, and prescriber time) associated with short and long prescription lengths, a model originally used by Walton et al.[5] was adapted and applied to the prescription data from the five cohorts (Appendix VI), and the two equations below were used, where 'C' represents cost and 'Q' represent quantity. An example calculation is provided in Box 2.

1) $TUC_{<60}=(C_{wastage<60} + C_{dispensing<60} + C_{prescribertime<60}) \text{ x } (120/Q_{daysused<60}) - (C_{dispensing<60} + C_{prescribertime<60})$

For peer review only - http://bmjopen.bmj.com/site/about/guidelines.xhtml

2) $TUC_{\geq 60}=(C_{wastage\geq 60} + C_{dispensing\geq 60} + C_{prescribertime\geq 60}) \times (120/Q_{daysused\geq 60}) - (C_{dispensing\geq 60} + C_{prescribertime\geq 60})$

One-way sensitivity analyses were conducted to examine differences in TUC under a variety of different scenarios, including scenarios assuming nurses issued the prescription instead of a GP, excluding prescriber time costs, accounting for changes in NHS revenue from patient charges per prescription, +/- 50% mean days wasted, +/- 50% the mean cost of drugs per day, dispensing fees, and prescriber time.

All statistical analyses were performed using Stata/MP 13.1 (Stata Corp LP, College Station, Texas, USA). The protocol (16_117R) for this study was approved on June 21, 2016 by the Independent Scientific Advisory Committee (ISAC), the independent body that approves use of CPRD data (Appendix VII).

RESULTS

Overall cohort selection

The proportion of observations dropped from the full sample due to missing or observations equal to zero in either the ndd or qty variables ranged from 6% in both the lipid management and hypertension cohorts to 21% in the glucose control in T2DM cohort. The numbers of observations were further reduced after accounting for prescription error (Appendix II).

Medication wastage

Over the 11-year study period there was a statistically significant difference in the proportion of days' supply wasted, mean number of day's supplied wasted and the mean cost of wastage per prescription between the short and long prescriptions groups for all five of the case studies (Appendix VIII). The proportion of days' supply wasted was consistently larger

For peer review only - http://bmjopen.bmj.com/site/about/guidelines.xhtml

for the long prescription group across all cohorts except depression where the short group had 6.3% of days' supply wasted compared to 3.7% in the longer group. The mean number of days' supply wasted was also consistently larger for the longer group, but the difference between the two prescription length groups was much smaller for the depression cohort in comparison to the other four cohorts.

Medication wastage by treatment pattern

In four of the five cohorts, mean cost of wastage per prescription was significantly higher with longer prescription lengths for all four treatment patterns (Table 2). The one exception was for the depression cohort where the mean cost of wastage per prescription for both dosage/formulation and within class treatment switches did not show statistically significant differences between the two prescription length groups. The refill treatment pattern consistently had the largest mean cost of wastage per prescription across the cohorts, particularly for the longer groups, except for the depression cohort. The lipid management cohort did not report any between class treatment switches as all medications included in the analysis were from the same class of statins.

Medication wastage over time

On an annual basis, mean cost of wastage per prescription was significantly higher in the longer prescription lengths for each study year, except 2012 and 2013 for depression (Appendix IX). In general, the magnitude of the mean costs remained relatively consistent over the study period, except for a few notable trends (Figure 1). In the glucose control in T2DM cohort the mean costs for the longer group in years 2004 and 2011 were slightly larger (range £1.81 to £3.14) compared to the other nine annual means (range £0.87 to £1.40). In the hypertension cohort there was a slight trend of decreasing magnitude of the mean cost over the 11 years for the shorter group; a decrease in mean cost was limited to years 2013 and 2014 in the longer group. For both the lipid management and secondary prevention of

For peer review only - http://bmjopen.bmj.com/site/about/guidelines.xhtml

myocardial infarction cohorts the magnitude of the mean costs remained relatively consistent over the 11 years for the shorter prescription length groups, whereas there was a slightly decreasing trend in the magnitude of the mean costs for the longer prescription length groups.

Differences in total unnecessary costs for short and long prescription lengths

TUC (wastage, dispensing fees and prescriber time) per 120-days was lower in the longer prescription group for all five cohorts (savings of £8.38 [glucose control in T2DM] to £12.06 [secondary prevention of MI] per prescription per 120 days if a single long prescription were issued instead of multiple short prescriptions, Appendix X). This roughly translates into savings of £25.14 to £36.18 per patient per year assuming patients would receive three prescriptions each with a 120-day supply instead of 12 prescriptions each with a 30-day supply.

Sensitivity analysis shows longer prescriptions remained cost saving compared with shorter prescriptions across all scenarios and ranges tested. The magnitude of the savings was lowest when prescriber time costs were excluded from the models (range £0.91 to £2.81 per prescription per 120 days) and reduced to a lesser extent when nurse prescriber time costs were used instead of physician's (range £5.94 to £8.48 per prescription per 120 days) and when loss of revenue to the NHS through a reduced number of prescription charges paid by patients was incorporated into the models (range £6.52 to £9.83 per prescription per 120 days). The other scenarios tested had relatively little impact on the magnitude of the savings, with the exception of increases and decreases of 50% in the cost of prescriber time (Appendix X).

For peer review only

For peer review only - http://bmjopen.bmj.com/site/about/guidelines.xhtml

DISCUSSION

Summary of findings

Longer prescription lengths are associated with more medication wastage per prescription compared to shorter prescription lengths. However, after taking into account transaction costs, longer prescription lengths are associated with overall cost savings (lower TUC) compared with shorter ones. In all five cohorts, most prescriptions were for ≤30 days with relatively small proportions of patients having prescription lengths between 31 and 60 days. Ninety five percent of prescriptions in the depression cohort were for <60 days. Some 39 million prescriptions are issued for antidepressants in the UK each year,[16] therefore, if the 95% issued as <60 day supplies were instead issued as longer ≥60 day prescriptions the total savings to the NHS could be as much as £408 million per year. Similarly, knowing 97.05% of statin prescriptions were issued as <60 day prescriptions from our CPRD analysis, the total savings to the NHS just in England could be as much as £563 million per year if the ~61.1 million[17] short statin prescriptions issued in 2015 for two statins (simvastatin and atorvastatin) were changed to longer prescriptions. However, it is critical to note that the majority of savings for both examples will not be cash releasing, but will be realised as savings in GP time, which could be used to increase primary care consultations with patients. Cash-releasing savings may come from reduced dispensing fees, for which we estimate an upper limit of £104 million and £62 million for antidepressants and the statins respectively. The magnitude of the savings for the other case studies will be of a similar scale given the prevalence of the conditions and frequency of shorter prescriptions. These figures should be interpreted with caution as they assume it is clinically appropriate for all prescriptions to be issued for a longer duration, which will certainly not be the case.

For peer review only - http://bmjopen.bmj.com/site/about/guidelines.xhtml

Comparison to previous studies

Several other studies have examined the costs associated with issuing either long (three-month) or short (one-month) supplies of prescriptions.[2-7] These studies all take the perspective of various payers in the US (e.g., different state-level Veterans Affairs and Medicaid programs as well as a non-institutionalised civilian population) and account for different cost items. Two studies found savings associated with longer prescriptions of a similar magnitude to ours, for example TUC of US$2.45 (£1.63 at April 2015 exchange rates)[5] and US$6.17 (£4.10)[3]. The former study[5] excluded prescriber time (the equivalent figure in our study is £1.03), and the latter[3] included costs of mail-order prescriptions.

Another study calculated per patient per year savings of US$7.70 (statins) to US$26.86 (oral hypoglycaemics) associated with 90- vs 30-day prescriptions.[6] A study of the financial impact on health care payers[4] detected statistically significant savings with 3-month supplies in only two of six cases as most savings accrued to patients through reductions in out-of-pocket costs. This study did not consider the cost of medication wastage making comparison with our study difficult. A simulation study found that any savings from reduced wastage from a shorter prescription length were more than offset by increases in dispensing fees as long as the dispensing fee was at least US$2.40 (base case assumption was US$5.60).[2]

In contrast to these, a comprehensive study on the impact of a policy to reduce the maximum prescription length from 100 to 34 days' supply in the North Carolina Medicaid program[7] found that total Medicaid expenditures (comprising outpatient, inpatient, emergency as well as pharmacy costs) decreased for patients initially receiving 100 day prescriptions after the implementation of the 34-day policy (range US$245 to $440 per-person per-quarter across six classes of medications [anti-hypertensives, anti-diabetic medications, lipid-lowering drugs, seizure-disorder medications, antidepressants and antipsychotics] assessed). However, the results are not broken down by expenditure category

For peer review only - http://bmjopen.bmj.com/site/about/guidelines.xhtml

(except for reporting decreases in expenditures for the targeted prescriptions across all six medication classes) and therefore it is unclear where the savings are accrued. This finding may be explained by small adverse health effects as a result of changes in adherence, patients absorbing any health effects through informal care or tolerating greater disease burden, or the follow-up period of the study (18 months post implementation) being too short to capture any spill-over effects of decreased adherence on other Medicaid services. The equivalent impact on NHS expenditure in the UK may differ due to differences in the organisation of care, in particular the gate-keeper role of primary care. Analysis of this was outside the scope of our analysis but would be a valuable future line of enquiry.

Study limitations

To the best of the authors' knowledge, this study provides the only evidence of the unnecessary costs associated with different prescription lengths from the perspective of the NHS in the UK and builds on existing methodological approaches available in the literature. However, there are a few limitations that warrant discussion. First, CPRD prescription data only indicates whether a prescription has been issued and not whether it was dispensed or taken as recommended. Our estimates may, therefore, either over- or understate the amount of wastage that actually occurred, depending on patient behaviour not captured in CPRD.

Second, the five case study conditions were purposively rather than randomly selected to represent the impact of medication refill and switching behaviour on wastage; they may not be representative of prescribing behaviour in other chronic conditions. However, those selected do represent some of the most common chronic conditions treated with prescribed medications. Nine of the top 20 prescribed medications within NHS England were included in at least one of the case study conditions in our analyses and combined they accounted for around £378 million (4%) of all drug expenditure within NHS England in 2015 and are therefore highly policy relevant.[17] Our analysis also excluded patients having one or more

For peer review only - http://bmjopen.bmj.com/site/about/guidelines.xhtml

observations with missing or zero values for either the ndd and/or qty variables. If this was non-random then the subsequent samples may not be truly representative of the general population. Appropriate methods to impute these variables are of limited value and our approach was similar to other studies using CPRD data.[9 10]

Third, the identification of patients within CPRD for the five case studies (Table 1) were based solely on product codes, rather than in conjunction with medical diagnoses. This was straight forward for four of the five case studies, but required additional assumptions for the secondary prevention of MI cohort. As the main aim of our study was to estimate drug wastage, the possible inclusion of patients without a previous MI event, but still receiving at least four of the prescriptions of interest for at least a year, provided our analysis with relevant information concerning drug wastage, dispensing fees and prescriber time.

Fourth, an overlap of dates between prescriptions does not necessarily mean wastage has occurred as consumption of early refills may be delayed until the initial supply is exhausted and treatment changes might actually be add-ons to existing prescriptions or concomitant therapy rather than switches in therapy. To ensure wastage was not overestimated a threshold of one year after the initial prescription in a particular series was used to estimate wastage for early refills and a threshold of <1 in the difference between the number of drug changes between medications with similar clinical indications from different classes and the number of unique drug classes within an annual period was used to identify wastage from between class treatment switches. There is, however, the possibility that our analysis approach could be overestimating the amount of medication wastage.

Fifth, for pragmatic purposes we dichotomised prescription lengths into 'short' versus 'long', with a cut-off of 60 days. This will have classified 56 day prescriptions as 'short'. Whilst this will have resulted in a loss of sensitivity (there may be differences in TUC

For peer review only - http://bmjopen.bmj.com/site/about/guidelines.xhtml

between one and two month prescriptions), the overall conclusions comparing 'shorter' (<60 day) and 'longer' (≥60 day) lengths are not affected.

Finally, a number of assumptions were required to assign unit costs to the estimated proportions of wastage. Mean cost per day values derived using DDDs, NICs and quantities at the drug substance level were calculated and then applied to any prescription categorised under that particular drug substance. This approach is not ideal, but necessary given the inability to link CPRD data to individual unit costs specific for each prescription. The direction and magnitude of any resulting bias is difficult to predict.

Furthermore, NICs do not include any discounts that may be applied or include any adjustment for revenue received by the NHS where a prescription charge is paid at the time the prescription is dispensed or where the patient has purchased a pre-payment certificate, and therefore maybe different from the net cost incurred specifically by the NHS. Patients with T2DM are exempt from the prescription charge,[18] and overall almost 90% of prescriptions dispensed in the NHS in England are exempt.[19]

All these limitations risk biasing the results. The projected savings should therefore be interpreted with caution and in any case be considered upper limits. Our analysis focused on drugs with low unit costs prescribed to large numbers of patients. The results may not be generalizable to high cost drugs used to treat relatively small patient groups.

CONCLUSIONS

Overall, the findings from the study indicate that from the perspective of the NHS in the UK, longer prescription lengths are cost-saving relative to shorter prescription lengths in a number of common chronic diseases. Policy-makers should recommend that GPs consider issuing longer prescriptions for common chronic conditions where clinically appropriate to

For peer review only - http://bmjopen.bmj.com/site/about/guidelines.xhtml

minimise the costs associated with dispensing fees and prescriber time as a result of issuing multiple prescriptions of shorter length.

For peer review only

For peer review only - http://bmjopen.bmj.com/site/about/guidelines.xhtml

CONTRIBUTORS

BD designed the study protocol, extracted, analysed and interpreted the data; drafted and revised this article; and gave final approval of this version to be published. AH extracted and assisted in organising the data, reviewed and edited the draft article and gave final approval of this version to be published. RP and EW conceptualised the study, assisted with its design and the interpretation of data, critically reviewed and edited the draft article and gave final approval of this version to be published.

COMPETING INTERESTS

All authors have no relevant affiliations or financial involvement with any organization or entity with a financial interest in or financial conflict with the subject matter or materials discussed in the manuscript, except support from a government grant as detailed in the acknowledgements. This includes employment, consultancies, grants, honoraria, stock ownership or options, expert testimony, or patents received or pending royalties. No writing assistance was utilised in the production of this manuscript.

DATA SHARING STATEMENT

No additional data available.

For peer review only

For peer review only - http://bmjopen.bmj.com/site/about/guidelines.xhtml

REFERENCES

1. Organization for Economic Cooperation and Development (OECD). Pharmaceutical expenditure. Health at a Glance 2011: OECD Indicators: OECD Publishing, 2011.
2. Domino ME, Olinick J, Sleath B, et al. Restricting patients' medication supply to one month: saving or wasting money? Am J Health Syst Pharm 2004;**61**(13):1375-9.
3. Parikh SP, Dishman BR, Smith TL. Ninety-day versus thirty-day drug-dispensing systems. Am J Health Syst Pharm 2001;**58**(13):1190-1.
4. Rabbani A, Alexander GC. Cost savings associated with filling a 3-month supply of prescription medicines. Appl Health Econ Health Policy 2009;**7**(4):255-64.
5. Walton SM, Arondekar BV, Johnson NE, et al. A model for Comparing Unecessary Costs Associated with Various Prescription Fill-Quantity Policies: illustration Using VA Data. J Managed Care Pharm 2001;**7**(5):384-90.
6. Taitel M, Fensterheim L, Kirkham H, et al. Medication Days' Supply, Adherence, Wastage, and Cost Among Chronic Patients in Medicaid. Medicare & Medicaid Research Review 2012;**2**(3):E1-E13.
7. Domino ME, Martin BC, Wiley-Exley E, et al. Increasing time costs and copayments for prescription drugs: an analysis of policy changes in a complex environment. Health Serv Res 2011;**46**(3):900-19.
8. Herrett E, Gallagher AM, Bhaskaran K, et al. Data Resource Profile: Clinical Practice Research Datalink (CPRD). Int J Epidemiol 2015;**44**(3):827-36.
9. Brodie MJ, Chung S, Wade A, et al. Clobazam and clonazepam use in epilepsy: Results from a UK database incident user cohort study. Epilepsy Res 2016;**123**:68-74.
10. Francis NA, Hood K, Lyons R, et al. Understanding flucloxacillin prescribing trends and treatment non-response in UK primary care: a Clinical Practice Research Datalink (CPRD) study. J Antimicrob Chemother 2016;**71**(7):2037-46.
11. WHO Collaborating Centre for Drug Statistics Methodology. ATC/DDD Index 2016. 16/12/2015 ed. Oslo, Norway: WHO, 2016.
12. National Health Service (NHS) - Prescription Services. Prescription Cost Analysis (PCA) Data. London, UK: NHS, December 2015.
13. National Health Service (NHS) - Prescription Services. Electronic Drug Tariff. Surrey, UK: NHS, December 2015.
14. Hollingworth W, Devine EB, Hansen RN, et al. The impact of e-prescribing on prescriber and staff time in ambulatory care clinics: a time motion study. J Am Med Inform Assoc 2007;**14**(6):722-30.
15. Curtis L, Burns A. *Unit Costs of Health and Social Care 2015*. Canterbury, UK: Personal Social Services Research Unit, The University of Kent, 2015.
16. Coupland C, Dhiman P, Morriss R, et al. Antidepressant use and risk of adverse outcomes in older people: population based cohort study. BMJ 2011;**343**:d4551.
17. National Health Service (NHS) - Business Services Authority. Prescription Cost Analysis (PCA) Data, 2015.
18. National Health Service (NHS) Choices. NHS in England - help with health costs. Secondary NHS in England - help with health costs 2016. http://www.nhs.uk/NHSEngland/Healthcosts/Pages/Prescriptioncosts.aspx.
19. Health and Social Care Information Centre. *Prescriptions dispensed in the community; England 2005-15*. Leeds: Health and Social Care Information Centre, 2016.
20. National Institute for Health and Care Excellence (NICE). British National Formulary (BNF). London, UK: NICE, 2016.
21. National Institute for Health and Care Excellence (NICE). *Type 2 diabetes in adults: management (NG28)*. London, UK: NICE, December 2015.

For peer review only - http://bmjopen.bmj.com/site/about/guidelines.xhtml

22. National Institute for Health and Care Excellence (NICE). *Myocardial infraction: cardiac rehabilitation and prevention of further MI (CG172)*. London, UK: NICE, November 2013.
23. Westbrook JI, Li L, Georgiou A, et al. Impact of an electronic medication management system on hospital doctors' and nurses' work: a controlled pre-post, time and motion study. J Am Med Inform Assoc 2013;**20**(6):1150-8.
24. Franklin BD, O'Grady K, Donyai P, et al. The impact of a closed-loop electronic prescribing and administration system on prescribing errors, administration errors and staff time: a before-and-after study. Qual Saf Health Care 2007;**16**(4):279-84.
25. Laurant M, Reeves D, Hermens R, et al. Substitution of doctors by nurses in primary care. Cochrane Database Syst Rev 2005(2):CD001271.
26. Shum C, Humphreys A, Wheeler D, et al. Nurse management of patients with minor illnesses in general practice: multicentre, randomised controlled trial. BMJ 2000;**320**(7241):1038-43.
27. Venning P, Durie A, Roland M, et al. Randomised controlled trial comparing cost effectiveness of general practitioners and nurse practitioners in primary care. BMJ 2000;**320**(7241):1048-53.

For peer review only

Table 1. Case study conditions and associated prescriptions

Case study	Relevant prescriptions/patient inclusion criteria
glucose control with oral drug therapy in type II diabetes mellitus	patients receiving one or more prescriptions for an oral anti-diabetic drug listed under the BNF Section 6.1.2 Antidiabetic drugs in any year from 2004 to 2014
treatment of hypertension in type II diabetes mellitus	in addition to receiving an oral anti-diabetic drug as defined in (1), patients receiving one or more prescriptions for any angiotensin-converting enzyme inhibitors, angiotensin II receptor antagonists, calcium-channel blockers, beta-adrenoceptor blockers, alpha-adrenoceptor blockers, potassium-sparing diuretics and/or thiazide-like diuretics in any year from 2004 to 2014
treatment with statins (lipid management) in type II diabetes mellitus	in addition to receiving an oral anti-diabetic drug as defined in (1), patients receiving one or more prescriptions for a statin in any year from 2004 to 2014
treatment for the secondary prevention of myocardial infraction	in addition to receiving concurrent[a] prescriptions for an angiotensin-converting enzyme inhibitor, antiplatelet and statin for at least one year in duration, patients receiving one or more prescriptions for beta-adrenoceptor blockers and/or angiotensin II receptor antagonists in any year from 2004 to 2014
treatment of depression	patients receiving one or more prescriptions for any anti-depressant drug listed under BNF Section 4.3 Antidepressant drugs in any year from 2004 to 2014

BNF – British National Formulary

[a]All patients receiving at least one prescription for an angiotensin-converting enzyme inhibitor, antiplatelet drug and statin were first identified in CPRD. Patients from this sample that did not have at least four prescriptions (chosen to represent one year of therapy) for each of these drugs in at least one of the 11 years of data available (i.e., 2004 to 2014) were excluded. From the remaining patients the additional constraint of receiving one or more prescriptions for any beta-adrenoceptor blockers and/or angiotensin II receptor antagonists was applied to define the full sample.

For peer review only

For peer review only - http://bmjopen.bmj.com/site/about/guidelines.xhtml

Table 2. Comparison of the mean cost of medication wastage per prescription over ten-year period 2004-2014 by treatment pattern (2015 £)

	**Mean cost of refill wastage per prescription 2015 £ (95% CI)**		**Mean cost of dosage/ formulation switch wastage per prescription**		**Mean cost of within class treatment switch wastage per prescription 2015 £ (95% CI)**		**Mean cost of treatment between class switch wastage per prescription 2015 £ (95% CI)**	
	**<60 days**	**≥60 days**	**<60 days**	**≥60 days**	**<60 days**	**≥60 days**	**<60 days**	**≥60 days**
Glucose control with oral drug therapy in T2DM	0.230 (0.226-0.233)	1.035 (0.772-1.298)	0.059 (0.058-0.061)	0.173 (0.143-0.204)	0.031 (0.029-0.032)	0.097 (0.080-0.114)	0.009 (0.008-0.010)	0.064 (0.051-0.078)
Hypertension in T2DM	0.050 (0.049-0.051)	0.271 (0.228-0.314)	0.038 (0.038-0.039)	0.128 (0.107-0.149)	0.004 (0.003-0.004)	0.013 (0.009-0.016)	0.003 (0.003-0.003)	0.026 (0.022-0.030)
Lipid management in T2DM	0.017 (0.017-0.017)	1.099 (0.832-1.367)	0.024 (0.023-0.024)	0.153 (0.081-0.225)	0.008 (0.007-0.008)	0.173 (0.075-0.271)	NA	
Secondary prevention of myocardial infraction	0.043 (0.042-0.043)	0.439 (0.300-0.578)	0.014 (0.014-0.014)	0.040 (0.036-0.045)	0.009 (0.009-0.009)	0.029 (0.027-0.031)	0.00005 (0.00004-0.00006)	0.0006 (0.0003-0.0008)
Depression	0.044 (0.042-0.046)	0.214 (0.180-0.249)	0.146 (0.143-0.150)	0.141 (0.113-0.169)	0.006 (0.005-0.007)	0.013 (0.004-0.021)	0.012 (0.010-0.013)	0.061 (0.036-0.086)

CI – confidence interval; NA – not applicable; T2DM – type II diabetes mellitus

For peer review only - http://bmjopen.bmj.com/site/about/guidelines.xhtml

Box 1. Example of differentiating between treatment switches and add-ons for a patient receiving medications for hypertension

In 2011, the patient has one change between clinically related drugs from different classes (ramipril to losartan) and receives medication belonging to two unique drug classes (ACE and ARA). One minus two is <1, so this change is considered a switch. The rationale being, if the number of changes was small or large and the number of unique drugs involved in the changes were also small or large respectively, switches in therapies were occurring and therefore there was potential for wastage to occur. In 2012, the patient has two changes between clinically related drugs from different classes (losartan to diltiazem and diltiazem to losartan) and receives medication belonging to two unique drug classes (ARA and CCB). Two minus two is <1, which indicates a switch, but in the treatment of hypertension ARAs and CCBs are commonly administered together as second-line therapy[20] and therefore these two changes were considered add-ons/concomitant therapy. In 2013, the patient has four changes between clinically related drugs from different classes (losartan to doxazosin, doxazosin to losartan, losartan to doxazosin and doxazosin to losartan) and receives medication belonging to two unique drug classes (ARA and AAB). Four minus two is ≥1, which indicates the four changes are add-ons, not switches. The rationale being that if the number of changes was large, but the number of unique drugs involved in the changes was low, an add-on or concomitant therapy was being prescribed and no wastage was occurring.

Year	Sequence of Prescriptions in Year	Drug	Class	Total Number of Treatment Switches Between Classes in Year (A)	Total Number Unique Classes in Year (B)	Difference for Year (A) - (B)	Count as Treatment Switch Between Classes[a]	Count as Add On
2011	1	Ramipril	ACE	1	2	-1	No	No
2011	2	Losartan potassium	ARA				Yes	No
2011	3	Losartan potassium	ARA				No	No
2011	4	Losartan potassium	ARA				No	No
2012	1	Losartan potassium	ARA	2	2	0	No	No
2012	2	Diltiazem hydrochloride	CCB				No	Yes
2012	3	Diltiazem hydrochloride	CCB				No	No
2012	4	Losartan potassium	ARA				No	Yes
2013	1	Losartan potassium	ARA	4	2	2	No	No
2013	2	Doxazosin	AAB				No	Yes
2013	3	Losartan potassium	ARA				No	Yes
2013	4	Doxazosin	AAB				No	Yes
2013	5	Losartan potassium	ARA				No	Yes

AAB – alpha-adrenoceptor blocker; ACE - angiotensin-converting enzyme inhibitor; ARA – angiotensin-II receptor antagonist; CCB – calcium channel blocker

[a]For the treatment of hypertension in T2DM cohort, overlaps in prescription dates involving angiotensin-converting enzyme inhibitors and angiotensin-II receptor antagonists with either calcium-channel blockers or thiazide-like diuretics were not counted as switches as these therapies are commonly administered together as second-line therapy.[20]

For peer review only - http://bmjopen.bmj.com/site/about/guidelines.xhtml

Box 2. Example comparing total unnecessary costs for <60 day and ≥60 day prescription lengths for a standardised time period of 120 days

Assume on average that the <60 day prescription length is 35 days and the average ≥60 day prescription lengths is 120 days. Also assume that regardless of prescription length, patients on average switch their prescription 30 days after a prescription is issued. The quantity used is therefore 30 days for both prescription lengths ($Q_{daysused<60} = Q_{daysused\geq60} = 30$), but the quantity wasted is much larger for the ≥60 day prescription (90 days compared to only five days wasted). Since over a 120-day period both prescription lengths will incur the same dispensing fees and prescriber time costs (four prescriptions will be issued regardless of prescription length as a switch occurs every 30 days) the ≥60 day prescription will be associated with higher total unnecessary costs. Note this example has been developed by adapting an example provided by Walton et al.[5] to the prescription lengths considered in our study.

For peer review only - http://bmjopen.bmj.com/site/about/guidelines.xhtml

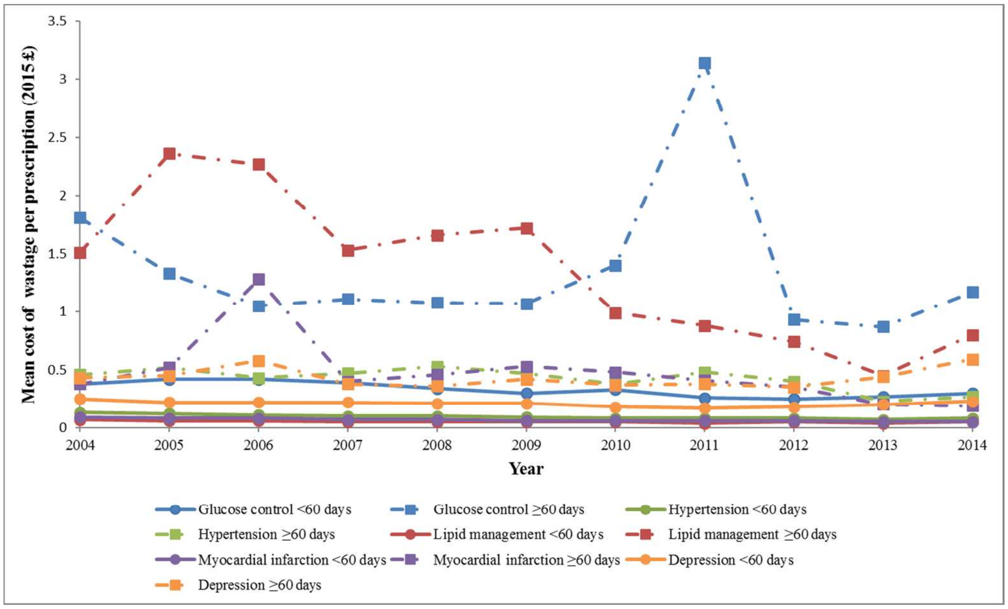

237x142mm (150 x 150 DPI)

Appendix I – CPRD product code lists of the potentially prescribed medications for each of the five case study conditions

Appendix I is available from the authors upon request as an Excel file containing five worksheets (one for each of the five case study conditions). Within each worksheet the lists of product codes obtained from the CPRD Research Applications Code Browser Version 3.0.0.0 that represent the possible medications that patients may be prescribed for the treatment of one of the five case study conditions is presented. The table below provides an example of the codes listed available in the associated Excel file. Please note that the lists in the table below are not comprehensive and readers should request the associated Excel file for the complete product code lists.

CPRD product code list

Study condition	Product code
Glucose control with oral drug therapy in type II diabetes mellitus	2219
	7912
	16602
	26218
	41558
Treatment of hypertension in type II diabetes mellitus	2
	58
	1209
	1211
	1213
Lipid management in type II diabetes mellitus	65193
	63140
	55034
	51200
	7374
Secondary prevention of myocardial infraction	59699
	56850
	34544
	3310
	26995
Treatment of depression	2525
	48065
	4690
	8831

For peer review only

Appendix II - Data processing of the five cohorts

Condition	Full sample (Patients)	Full sample (Rx)	ndd=0 or qty=0 (Patients)	ndd=0 or qty=0 (Rx)	Limited sample[a] (Patients)	Limited sample[a] (Rx)	Random sample (Patients)	Random sample (Rx)	Dropped prescription error[b] (Rx)	Final sample (Rx)	Same day switches not wastage[c] (Rx)
Glucose control with oral drug therapy in T2DM	310,391	21,091,529	170,967	4,518,765	139,424	7,135,397	50,000	2,577,282	6,483	2,570,799	548,850
Hypertension in T2DM	230,760	23,886,597	63,802	1,446,199	166,958	16,041,452	50,000	4,803,444	3,983	4,799,461	1,588,921
Lipid management in T2DM	242,741	13,388,759	36,577	776,718	206,164	11,216,086	50,000	2,718,216	913	2,717,303	NA
Secondary prevention of myocardial infraction	208,682	44,151,527	87,281	3,270,504	121,401	24,479,014	50,000	10,131,377	767	10,130,610	5,856,361
Depression	1,207,523	32,744,994	424,446	4,438,319	783,077	15,712,941	50,000	1,010,463	3,234	1,007,229	12,401

ndd – numeric daily dose; NA – not applicable; qty – quantity; Rx – prescriptions; T2DM – type II diabetes mellitus

[a]The limited sample consists of all patients that have received at least one of the relevant prescriptions for a respective case study condition and do not have any missing or observations equal to zero for both the ndd and qty variables.

[b]Prescriptions issued on the same day for medications in the same class and with different product codes (e.g., two different selective serotonin reuptake inhibitors or two different statins) were considered prescriber error and dropped from the analysis.

[c]Prescriptions for medications with similar clinical indications from different classes issued on the same day were assumed not to incur wastage due to a treatment switch, but rather were assumed to be an add-on to therapy or concomitant therapy.

For peer review only

Appendix III – Description of methods used to estimate medication wastage

Wastage was defined as either a repeat prescription or new prescription based on the three types of substitutions/switches (substitutions between different dosages or formulations of the same drug substance; substitutions between drugs that are in the same class; and substitutions between drugs that have similar clinical indications from different classes) respectively, being issued prior to the expiry of the previously prescribed quantity. The volume of medication wastage from early refills and treatment switches was estimated for prescriptions within the 11-year study period (i.e., any prescription not falling in this time period were excluded from the analyses). This was done by dividing the total quantity entered by the GP for the prescribed product (qty) by the numeric daily dose prescribed for the event (ndd) and comparing this to the difference in the two dates associated with the event and the next prescription in the sequence. Prescriptions issued on the same day for drugs in the same class with different product codes (e.g., two different statins) were considered prescriber error and dropped from the analysis. The one exception to this was for antiplatelet drugs in the secondary prevention of myocardial infarction cohort, as it was assumed that two different antiplatelet drugs could be prescribed at the same time. In addition, prescriptions for medications with similar clinical indications from different classes issued on the same day were not counted as a switch, but rather as an add-on to existing therapy or concomitant therapy.

As treatment patterns were assessed in sequence there was the potential to overstate the amount of wastage that occurred. For example, counting every overlap of prescription days associated with refills of the same product as wastage (i.e., even prescriptions issued one day before the expiry of the previous prescription would be counted as one day's worth of medication wastage or two prescriptions issued on the same day would count one prescription as entirely wasted) may overstate actual wastage. A threshold of one year after the initial

For peer review only

prescription in a particular series was, therefore, used to estimate wastage for early refills (i.e., repeat prescriptions), in that any product prescribed over and above the expected quantity to be consumed within the one-year time period was considered waste. This is to account for the fact that patients may fill their prescriptions before their existing supply is exhausted, but still consume all of the previous prescription before using the new supply.

In addition, while excessive switching of drugs could appear as wastage, consistent patterns could suggest a valid, prescribed treatment regimen. To avoid the overestimation of wastage, additional effort was made to differentiate between add-ons/concomitant therapy and switches for medications with similar clinical indications from different classes (i.e., product, drug substance and drug class codes are different for two prescriptions in sequence). Generally, if the difference between the number of changes between medications with similar clinical indications from different classes and the number of unique drug classes within an annual period was ≥1, then any overlap in prescription dates was not considered wastage due to a switch, but rather as an add-on or concomitant therapy. For example, within an annual period a patient may have six changes between clinically related drugs from different classes, but these changes are only between two different drugs from different classes (i.e., two unique drug classes). Since six minus two is ≥1 these changes were not considered switches, but rather add-on/concomitant prescriptions. The rationale being that if the number of changes was large, but the number of unique drugs involved in the changes was low an add-on or concomitant therapy was being prescribed, whereas if the number of changes was large and the number of unique drugs involved in the changes was also large, switches in therapies were occurring and therefore there was the potential for wastage to occur. A similar approach was applied by Taitel et al.[6] and is the only previous study that has attempted to differentiate between add-on/concomitant prescriptions and actual switches in therapy. Additional constraints in counting overlaps in dates for two prescriptions in sequence for medications

For peer review only

with similar clinical indications from different classes were also applied in three of the case study conditions due to the potential for a number of the included therapies to be given concomitantly. For the glucose control in T2DM cohort overlaps in prescription dates involving metformin with drug from other classes were not counted as switches (and therefore wastage) as metformin is usually administered in combination with other classes of oral anti-diabetics.[21] For the treatment of hypertension in T2DM cohort, overlaps in prescription dates involving angiotensin-converting enzyme inhibitors and angiotensin-II receptor antagonists with either calcium-channel blockers or thiazide-like diuretics were not counted as switches as these therapies are commonly administered together as second-line therapy.[20] Finally, for the secondary prevention of myocardial infraction cohort, only overlapping prescriptions dates involving beta-blockers and angiotensin-II receptor antagonists were counted as switches as patients are likely to receive the other classes of drugs included in the analysis (angiotensin-converting enzyme inhibitors, antiplatelet drugs and statins) continuously over the course of treatment.[22]

For peer review only

Appendix IV – Unit prescription drug cost calculations

Drug substance	DDD (mg)	NIC from PCA (pence)	Quantity from PCA	Strength from PCA (mg)	NIC/ Quantity (£)	Cost per day[a] (£)
**Initial glucose control in type II diabetes**						
glibenclamide	7	1,072,539	279,251	5	0.04	0.05
gliclazide	60	3,322,771	195,041	60	0.17	0.17
glimepiride	2	2,650,357	685,422	2	0.04	0.04
glipizide	10	8,679,376	762,384	5	0.11	0.23
tolbutamide	1500	17,494,096	266,373	500	0.66	1.97
metformin	2000	531,088,447	111,025,385	500	0.05	0.19
acarbose	300	108,607	1,234	100	0.88	2.64
alogliptin	25	6,918,604	10,147	25	6.82	6.82
canagliflozin	200	58,466,442	447,445	100	1.31	2.61
dapagliflozin	10	191,937,124	1,468,764	10	1.31	1.31
empagliflozin	17.5	16,291,281	124,666	10	1.31	2.29
exenatide	1	10,031,280	1,470	60	68.24	1.14
linagliptin	5	271,703,001	2,287,246	5	1.19	1.19
liraglutide	1.2	337,718,696	86,033	6	39.25	7.85
lixisenatide	0.02	45,851,757	15,830	0.28	28.97	2.07
nateglinide	360	631,585	17,827	180	0.35	0.71
pioglitazone	30	144,198,158	1,125,016	30	1.28	1.28
repaglinide	4	1,620,508	246,350	2	0.07	0.13
saxagliptin	5	80,113,342	709,874	5	1.13	1.13
sitagliptin	100	670,533,026	5,644,766	100	1.19	1.19
vildagliptin	100	18,582,165	312,012	50	0.60	1.19
rosiglitazone[b]	6	-	-	-	-	2.34
guar gum[b,d]	68.5	-	-	-	-	2.34
dulaglutide	0.16	1,717,714	938	0.75	18.31	3.91
**Hypertension in type II diabetes**						
bendroflumethiazide	2.5	115,395,532	38,916,033	2.5	0.03	0.03
chlortalidone	25	410	70	50	0.06	0.03
cyclopenthiazide[b]	0.5	-	-	-	-	1.07
indapamide	2.5	40,603,410	7,849,048	2.5	0.05	0.05
xipamide	20	274,581	19,761	20	0.14	0.14
chlorothiazide	500	92,160	200	500	4.61	4.61
hydrochlorothiazide	25	743,206	4,701	25	1.58	1.58
Hydroflumethiazide[b]	25	-	-	-	-	1.07
candesartan cilexetil	8	27,228,854	6,187,293	8	0.04	0.04
eprosartan	600	7,848,330	156,629	600	0.50	0.50
irbesartan	150	15,321,402	2,527,161	150	0.06	0.06
losartan potassium	50	43,142,921	10,854,773	50	0.04	0.04
olmesartan medoxomil	20	23,454,180	507,120	20	0.46	0.46
telmisartan	40	2,105,968	429,294	40	0.05	0.05
valsartan	80	7,680,126	790,946	80	0.10	0.10

For peer review only

Drug substance	DDD (mg)	NIC from PCA (pence)	Quantity from PCA	Strength from PCA (mg)	NIC/ Quantity (£)	Cost per day[a] (£)
captopril	50	1,209,326	274,077	50	0.04	0.04
enalapril maleate	10	8,273,970	2,164,274	10	0.04	0.04
fosinopril sodium	15	1,717,978	32,658	10	0.53	0.79
imidapril hydrochloride	10	523,685	20,308	10	0.26	0.26
lisinopril	10	30,787,577	8,978,102	10	0.03	0.03
moexipril hydrochloride	15	22,272	896	15	0.25	0.25
perindopril erbumine	4	25,785,508	604,0253	4	0.04	0.04
perindopril arginine	4	1,125,376	53,760	5	0.21	0.17
quinapril	15	2,130,784	69,778	10	0.31	0.46
ramipril	2.5	75,859,752	18,621,897	2.5	0.04	0.04
ramipril with felodipine	2.5	456,727	5,209	2.5	0.88	0.88
trandolapril	2	4,668,257	191,955	2	0.24	0.24
cilazapril	2.5	1,662,225	8,985	5	1.85	0.93
perindopril tosilate	4	11,821	594	5	0.20	0.16
amlodipine	5	153,321,726	47,137,956	5	0.03	0.03
diltiazem hydrochloride	240	3,855,905	93,720	240	0.41	0.41
felodipine	5	63,115,662	4,197,918	5	0.15	0.15
isradipine	5	4,429,879	13,442	2.5	3.30	6.59
lacidipine	4	14,646,423	910,750	4	0.16	0.16
lercanidipine hydrochloride	10	77,973,540	3,825,663	10	0.20	0.20
nicardipine hydrochloride	90	397,902	35,119	30	0.11	0.34
nifedipine	30	3,656,530	149,471	30	0.24	0.24
nisoldipine[b]	20	-	-	-	-	0.93
verapamil hydrochloride	240	7,104,750	358,394	240	0.20	0.20
doxazosin	4	52,769,814	14,187,844	4	0.04	0.04
indoramin[d]	4.7	5,674,414	34,4947	20	0.16	0.04
prazosin	5	74,527	205	5	3.64	3.64
terazosin	5	1,372,116	136,216	5	0.10	0.10
amiloride hydrochloride	10	18,835,995	565,354	5	0.33	0.67
amiloride hydrochloride with thiazide	10	868,138	75,026	5	0.12	0.23
triamterene	100	211,025	1,511	50	1.40	2.79
triamterene with thiazide	100	160,534	4,540	50	0.35	0.71
spironolactone	75	24,174,720	4,339,112	25	0.06	0.17
atenolol	75	25,567,900	8,522,336	25	0.03	0.09
**Hyperlipidaemia in type II diabetes**						
atorvastatin	20	158,989,904	31,324,266	20	0.05	0.05
fluvastatin	60	1,475,946	163,264	20	0.09	0.27

For peer review only

Drug substance	DDD (mg)	NIC from PCA (pence)	Quantity from PCA	Strength from PCA (mg)	NIC/ Quantity (£)	Cost per day[a] (£)
pravastatin	30	6,780,883	1,648,896	10	0.04	0.12
rosuvastatin	10	134,267,696	2,085,108	10	0.64	0.64
simvastatin	30	15,635,680	5,206,048	10	0.03	0.09
**Secondary prevention of myocardial infraction**						
captopril	50	1,209,326	274,077	50	0.04	0.04
enalapril maleate	10	8,273,970	2,164,274	10	0.04	0.04
fosinopril sodium	15	1,717,978	32,658	10	0.53	0.79
lisinopril	10	30,787,577	8,978,102	10	0.03	0.03
perindopril erbumine	4	25,785,508	6,040,253	4	0.04	0.04
perindopril arginine	4	1,125,376	53,760	5	0.21	0.17
quinapril	15	728,515	23,878	5	0.31	0.92
ramipril	2.5	75,859,752	18,621,897	2.5	0.04	0.04
ramipril with felodipine	2.5	456,727	5,209	2.5	0.88	0.88
trandolapril	2	4,668,257	191,955	2	0.24	0.24
cilazapril	2.5	1,662,225	8,985	5	1.85	0.93
perindopril tosilate	4	11,821	594	5	0.20	0.16
acebutolol	400	1,202,284	18,079	400	0.67	0.67
atenolol	75	25,567,900	8,522,336	25	0.03	0.09
bisoprolol	10	20,420,456	5,828,018	10	0.04	0.04
carvedilol	37.5	2,684,299	577,569	6.25	0.05	0.28
metoprolol tartrate	150	15,398,869	2,318,257	50	0.07	0.20
aspirin[d]	127.5	154,533,218	52,719,924	75	0.03	0.05
clopidogrel	75	129,484,315	19,793,087	75	0.07	0.07
ticagrelor	180	190,143,810	1,950,196	90	0.97	1.95
atorvastatin	20	158,989,904	31,324,266	20	0.05	0.05
fluvastatin	60	1,475,946	163,264	20	0.09	0.27
pravastatin	30	6,780,883	1,648,896	10	0.04	0.12
rosuvastatin	10	134,267,696	2,085,108	10	0.64	0.64
simvastatin	30	15,635,680	5,206,048	10	0.03	0.09
candesartan cilexetil	8	27,228,854	6,187,293	8	0.04	0.04
eprosartan	600	7,848,330	156,629	600	0.50	0.50
irbesartan	150	15,321,402	2,527,161	150	0.06	0.06
losartan potassium	50	43,142,921	10,854,773	50	0.04	0.04
olmesartan medoxomil	20	23,454,180	507,120	20	0.46	0.46
telmisartan	40	2,105,968	429,294	40	0.05	0.05
valsartan	80	7,680,126	790,946	80	0.10	0.10
**Depression**						
amitriptyline hydrochloride	75	42,327,326	12,089,330	25	0.04	0.11
amoxapine	150	65,772	168	100	3.92	5.87
clomipramine hydrochloride	100	5,343,700	663,607	50	0.08	0.16
dosulepin hydrochloride	150	9,835,231	1,582,426	75	0.06	0.12

For peer review only

Drug substance	DDD (mg)	NIC from PCA (pence)	Quantity from PCA	Strength from PCA (mg)	NIC/ Quantity (£)	Cost per day[a] (£)
doxepin[c]	100	-	-	50	0.20	0.41
imipramine hydrochloride	100	4,075,545	858,036	25	0.05	0.19
lofepramine	105	39,309,425	1,177,389	70	0.33	0.50
nortriptyline	75	115,762,972	1,077,134	25	1.07	3.22
trazodone hydrochloride	300	61,816,624	718,214	150	0.86	1.72
trimipramine	150	1,672	56	50	0.30	0.90
citalopram	20	86,323,713	23,901,861	20	0.04	0.04
escitalopram	10	5,540,518	1,121,092	10	0.05	0.05
fluoxetine	20	87,922,802	23,735,904	20	0.04	0.04
fluvoxamine maleate	100	4,073,827	55,899	100	0.73	0.73
paroxetine	20	25,034,806	3,116,825	20	0.08	0.08
sertraline	50	100,699,567	15,289,272	50	0.07	0.07
isocarboxazid	15	3,453,630	12,118	10	2.85	4.28
moclobemide	300	1,057,512	22,678	300	0.47	0.47
phenelzine	60	2,647,884	117,682	15	0.23	0.90
tranylcypromine	10	45,505,295	51,406	10	8.85	8.85
agomelatine	25	8,035,533	74,999	25	1.07	1.07
duloxetine	60	276,427,017	2,904,742	60	0.95	0.95
mirtazapine	30	26,199,391	4,793,099	30	0.05	0.05
reboxetine	8	4,467,383	141,747	4	0.32	0.63
tryptophan[d]	44.6	18,495	566	50	0.33	0.29
venlafaxine	100	17,440,533	3,256,789	75	0.05	0.07

DDD – defined daily dose; NIC – net ingredient cost; PCA – Prescription Cost Analysis

[a]Calculated as (NIC/Quantity) x (DDD/Strength from PCA).

[b]Data not available within the PCA for December 2015; cost per day based on an average of the values for other drugs within the same class.

[c]Data from PCA for December 2015 deemed unreliable; NIC/Quantity derived by dividing cost of 50 mg 28-cap pack by 28.

[d]Data for DDD not available; DDD based on an average of the values for other drugs within the same class.

For peer review only

Appendix V – Search strategy for prescriber time data

Date of Search: *July 8, 2016*

Databases: Ovid MEDLINE (R) Epub Ahead of Print, In-process & Other Non-Indexed Citations, Ovid MEDLINE (R) Daily and Ovid MEDLINE (R) 1946 to present; Embase 1974 to 2016 July 07

1	general practitioner.ab,hw,kf,kw,ot,ti,xs.
2	GP.ab,hw,kf,kw,ot,ti,xs.
3	physician.ab,hw,kf,kw,ot,ti,xs.
4	clinician.ab,hw,kf,kw,ot,ti,xs.
5	doctor.ab,hw,kf,kw,ot,ti,xs.
6	medic.ab,hw,kf,kw,ot,ti,xs.
7	consultant.ab,hw,kf,kw,ot,ti,xs.
8	medical specialist.ab,hw,kf,kw,ot,ti,xs.
9	physician assistant.ab,hw,kf,kw,ot,ti,xs.
10	physician associate.ab,hw,kf,kw,ot,ti,xs.
11	nurse.ab,hw,kf,kw,ot,ti,xs.
12	pharmacist.ab,hw,kf,kw,ot,ti,xs.
13	healthcare professional.ab,hw,kf,kw,ot,ti,xs.
14	medical professional.ab,hw,kf,kw,ot,ti,xs.
15	medical staff.ab,hw,kf,kw,ot,ti,xs.
16	1 or 2 or 3 or 4 or 5 or 6 or 7 or 8 or 9 or 10 or 11 or 12 or 13 or 14 or 15
17	prescriber time.ab,hw,kf,kw,ot,ti,xs.
18	staff time.ab,hw,kf,kw,ot,ti,xs.
19	time utilization.ab,hw,kf,kw,ot,ti,xs.
20	time utilisation.ab,hw,kf,kw,ot,ti,xs.
21	workload.ab,hw,kf,kw,ot,ti,xs.
22	workflow.ab,hw,kf,kw,ot,ti,xs.
23	work processes.ab,hw,kf,kw,ot,ti,xs.
24	medication management.ab,hw,kf,kw,ot,ti,xs.
25	17 or 18 or 19 or 20 or 21 or 22 or 23 or 24
26	time study.ab,hw,kf,kw,ot,ti,xs.
27	time motion study.ab,hw,kf,kw,ot,ti,xs.
28	time-motion study.ab,hw,kf,kw,ot,ti,xs.
29	(time and motion method).ab,hw,kf,kw,ot,ti,xs.
30	time-and-motion method.ab,hw,kf,kw,ot,ti,xs.
31	(time and motion study).ab,hw,kf,kw,ot,ti,xs.
32	time-and-motion study.ab,hw,kf,kw,ot,ti,xs.
33	time motion analysis.ab,hw,kf,kw,ot,ti,xs.
34	time-motion analysis.ab,hw,kf,kw,ot,ti,xs.
35	(before and after study).ab,hw,kf,kw,ot,ti,xs.
36	before-and-after study.ab,hw,kf,kw,ot,ti,xs.
37	26 or 27 or 28 or 29 or 30 or 31 or 32 or 33 or 34 or 35 or 36
**38**	16 and 25 and 37 – **Total Hits = 227**

For peer review only

Study selection details

The targeted literature search identified a total of 227 citations. After titles and abstracts were screened 216 citations were excluded and 11 citations were reviewed in full-text. Four studies contained relevant information and the most appropriate evidence was selected from the four studies by prioritising evidence from larger sample sizes and studies that reported prescriber time for different types of prescriptions (e.g., new versus renewals) and/or different types of prescribers (e.g., general practitioner versus nurse).[14 23-25] It should be noted that one of the four studies identified was a systematic review and led to the identification of two additional studies with relevant information based on their reporting of mean consultation times based on large sample sizes and in different types of prescribers. [26 27]

For peer review only

Appendix VI - Mean values used in the comparison of total unnecessary costs

	Glucose control with oral drug therapy in T2DM		Hypertension in T2DM		Lipid management in T2DM		Secondary prevention of myocardial infraction		Depression	
**Parameters**	**<60 days**	**≥60 days**	**<60 days**	**≥60 days**	**<60 days**	**≥60 days**	**<60 days**	**≥60 days**	**<60 days**	**≥60 days**
Mean days used ($Q_{daysused}$)	31.61	75.72	32.51	86.91	32.74	122.71	31.41	102.28	27.43	65.03
Mean days wasted ($Q_{dayswasted}$)	0.86	4.96	1.23	6.98	0.63	16.21	0.96	6.56	1.87	2.69
Mean drug cost per day (£) ($C_{drug/day}$)	0.36	0.28	0.084	0.082	0.10	0.10	0.074	0.068	0.14	0.11
Mean dispensing fee cost (£) ($C_{dispensing}$)	0.92	1.05	0.90	0.93	0.90	1.00	0.90	0.97	0.91	0.96
Mean prescriber (GP) time cost (£) ($C_{prescribertime}$)	3.39	3.44	3.54	3.55	3.12	3.15	3.76	3.77	3.23	3.18
Mean prescriber (Nurse) time cost (£) ($C_{prescribertime}$)	2.28	2.29	2.31	2.32	2.21	2.22	2.37	2.37	2.24	2.23

GP – general practitioner; T2DM – type II diabetes mellitus

For peer review only - http://bmjopen.bmj.com/site/about/guidelines.xhtml

Appendix VII – Study protocol (16_117R) approved on June 21, 2016 by the Independent Scientific Advisory Committee (ISAC)

ISAC APPLICATION FORM

PROTOCOLS FOR RESEARCH USING THE CLINICAL PRACTICE RESEARCH DATALINK (CPRD)

ISAC use only:<br>Protocol Number<br>Date submitted	 ....<br>............................	**IMPORTANT**<br>**If you have any queries, please contact ISAC Secretariat:**<br>ISAC@cprd.com

Section A: The study

1. Study Title

Three months versus 28 day prescriptions: a retrospective analysis of CPRD data to determine differences in the cost of drug wastage, dispensing fees and prescriber time

2. Has any part of this research proposal or a related proposal been previously submitted to ISAC?

Yes ☐ No ☒

If Yes, please provide previous protocol numbers:

3. Has this protocol been peer reviewed by another Committee? (e.g. grant award or ethics committee)

Yes ☒ No ☐

If Yes, please state the name of the reviewing Committee(s) and provide an outline of the review process and outcome: Proposal peer reviewed as part of a successful application to NIHR Health Technology Assessment Programme.

4. Type of Study (please tick all the relevant boxes which apply)

Adverse Drug Reaction/Drug Safety ☐
Drug Effectiveness ☐
Health/Public Health Services Research ☒
Other* ☐
Drug Utilisation ☒
Pharmacoeconomics ☒
Disease Epidemiology ☐
Methodological ☐
Post-authorisation Safety ☐

*Please specify the type of study in the lay summary

5. This study is intended for (please tick all the relevant boxes which apply)**:**

Publication in peer reviewed journals ☒
Presentation at company/institutional meetings ☐
Other: Publication in NIHR HTA Monograph
Presentation at scientific conference ☒
Regulatory purposes ☐

Section B: The Investigators

6. Chief Investigator (full name, job title, organisation name & e-mail address for correspondence- see guidance notes for eligibility)

Name: Ed Wilson
Job title: Senior Research Associate in Health Economics
Organisation: Cambridge Centre for Health Services Research, University of Cambridge
Email: ew442@medschl.cam.ac.uk

CV has been previously submitted to ISAC ☐ **CV number:**
A new CV is being submitted with this protocol ☒
An updated CV is being submitted with this protocol ☐

7. Affiliation (full address)

Institute of Public Health, Cambridge CB2 0SR, UK

8. Corresponding Applicant

Name: Brett Doble
Job title: Research Associate in Health Economics
Organisation: Cambridge Centre for Health Services Research, University of Cambridge
Email: brett.doble@medschl.cam.ac.uk

Same as chief investigator ☐
CV has been previously submitted to ISAC ☐ **CV number:**
A new CV is being submitted with this protocol ☒
An updated CV is being submitted with this protocol ☐

9. List of all investigators/collaborators (*please list the full names, affiliations and e-mail addresses* of all collaborators, other than the Chief Investigator*)

Other investigator: Rupert Payne,

For peer review only - http://bmjopen.bmj.com/site/about/guidelines.xhtml

Job title: Consultant Senior Lecturer in Primary Health Care
Organisation: University of Bristol
Email: rupert.payne@bristol.ac.uk

CV has been previously submitted to ISAC ☒ **CV number:** 177_15CEP
A new CV is being submitted with this protocol ☐
An updated CV is being submitted with this protocol ☐

Other investigator: Sarah King
Job title: Visiting Fellow
Organisation: University of Cambridge
Email: sek23@cam.ac.uk
CV has been previously submitted to ISAC ☐ **CV number:**
A new CV is being submitted with this protocol ☒
An updated CV is being submitted with this protocol ☐

[Please add more investigators as necessary] **Please note that your ISAC application form and protocol* ***must*** *be copied to all e-mail addresses listed above at the time of submission of your application to the ISAC mailbox. Failure to do so will result in delays in the processing of your application.*

10. Conflict of interest statement* (please provide a draft of the conflict (or competing) of interest (COI) statement that you intend to include in any publication which might result from this work)
The authors do not have any conflict of interest.
**Please refer to the International Committee of Medical Journal Editors (ICMJE) for guidance on what constitutes a COI*

11. Experience/expertise available (please complete the following questions to indicate the experience/expertise available within the team of investigators/collaborators actively involved in the proposed research, including the analysis of data and interpretation of results

	Previous GPRD/CPRD Studies	Publications using GPRD/CPRD data
None	☐	☒
1-3	☐	☐
> 3	☒	☐

	Yes	No
**Is statistical expertise available within the research team?**<br>*If yes, please indicate the name(s) of the relevant investigator(s)*<br>Rupert Payne and Ed Wilson with additional support provided by the Cambridge CPRD User Group, which includes Senior Statistician Dr. Katie Saunders.	☒	☐
**Is experience of handling large data sets (>1 million records) available within the research team?**<br><br>*If yes, please indicate the name(s) of the relevant investigator(s)*<br>Rupert Payne, Brett Doble<br>• Rupert Payne previously managed Cambridge's institutional license for CPRD, and is familiar with the CPRD GOLD dataset, analysis of HES records, and has relevant experience from analysis of other large, linked primary-secondary care datasets. He also has extensive programming experience as well as database management skills.<br>• Brett Doble has had experience managing, cleaning, and analysing large datasets in Australia (e.g., PBS, MBS, VAED, VEMD) during his PhD studies at Monash University. He has also recently had experience analysing UK GP practice prescribing data from the Health & Social Care Information Centre.	☒	☐
**Is experience of practising in UK primary care available within the research team?**<br><br>*If yes, please indicate the name(s) of the relevant investigator(s)*<br>Rupert Payne<br>• Dr Rupert Payne is a practising GP and Consultant Senior Lecturer in Primary Care at the School of Social and Community Medicine, University of Bristol	☒	☐

12. References relating to your study

Please list up to 3 references (most relevant) relating to your proposed study:

- Domino ME, Olinick J, Sleath B, Leinwand S, Byrns PJ, Carey T. Restricting patients' medication supply to one month: Saving or wasting money. Am J Health-Syst Pharm 2004;61:1375-1379.

For peer review only

For peer review only - http://bmjopen.bmj.com/site/about/guidelines.xhtml

Section C: Access to the data			
**13. Financial Sponsor of study**			
Pharmaceutical Industry	☐ *Please specify:*	Academia	☒ *Please specify:* NIHR Health Technology Assessment Programme
Government / NHS	☐ *Please specify:*	Charity	☐ *Please specify:*
Other	☐ *Please specify:*	None	☐
**14. Type of Institution carrying out the analyses**			
Pharmaceutical Industry	☐ *Please specify:*	Academia	☒ *Please specify:* Cambridge Centre for Health Services Research, University of Cambridge
Government Department	☐ *Please specify:*	Research Service Provider	☐ *Please specify:*
NHS	☐ *Please specify:*	Other	☐ *Please specify:*

For peer review only

15. Data source

The sponsor has direct access to CPRD GOLD and will extract the relevant data* ☒

A data set will be supplied by CPRD** ☐

CPRD has been commissioned to extract the relevant data and to perform the analyses ☐
Other ☐ *Please specify:*

*If data sources other than CPRD GOLD are required, these will be supplied by CPRD
** Please note that datasets provided by CPRD are limited in size. Applicants should contact CPRD (KC@CPRD.com) if a dataset of >300,000 patients is required.

16. Primary care data (please specify which primary care data set(s) are required)
Vision only (Default for CPRD studies) ☒
EMIS® only* ☐
Both Vision and EMIS®* ☐

Note: Vision and EMIS are different clinical systems, Vision data has traditionally been used for CPRD, EMIS is currently undergoing beta-testing.
**Investigators requiring the use of EMIS data must discuss the study with a member of CPRD staff before submitting an ISAC application*
Please list below the name of the person/s at the CPRD with whom you have discussed your request for EMIS data:

Section D: Data linkage

17. Does this protocol also seek access to data held under the CPRD Data Linkage Scheme?

Yes* ☐ No ☒

If No, please move to section E.

**Investigators requiring linked data must discuss the study with a member of CPRD staff. It is important to be aware that linked data are not available for all patients in CPRD GOLD, the coverage periods for each data source may differ and charges may be applied. Please contact the CPRD Research Team on +44 (20) 3080 6383 or email kc@cprd.com to discuss your requirements before submitting your application.*
Please list below the name of the person/s at the CPRD with whom you have discussed your request:

Please note that as part of the ISAC review of linkages, the protocol may be shared - in confidence - with a representative of the requested linked data set(s) and summary details may be shared - in confidence - with the Confidentiality Advisory Group of the Health Research Authority.

For peer review only - http://bmjopen.bmj.com/site/about/guidelines.xhtml

18. Please select the source(s) of linked data being requested:

☐ ONS Mortality Data
☐ Inpatient Hospital Episode Statistics
☐ Outpatient Hospital Episode Statistics
☐ NCDR Cancer Registry Data*
☐ MINAP
☐ Mother Baby Link

☐ Index of Multiple Deprivation
☐ Townsend Score
☐ Other** *Please specify:*

Please note that applicants seeking access to cancer registry data must provide consent for publication of their study title and study institution on the UK Cancer Registry website. They must also complete a **Cancer Dataset Agreement Form (available from CPRD) and provide a **System level Security Policy** for each organisation involved in the study.*

*** If "Other" is specified, please name an individual in CPRD that this linage has been discussed with.*

19. Total number of linked datasets requested including CPRD GOLD:

20. Is linkage to a local dataset with <1 million patients being requested?

Yes* ☐ No ☐

** If yes, please provide further details:*

21. If you have requested linked data sets, please indicate whether the Chief Investigator or any of the collaborators listed in response to question 5 above, have access to any of the linked datasets in a patient identifiable form, or associated with a patient index.

Yes* ☐ No ☐

** If yes, please provide further details:*

22. Does this study involve linking to patient *identifiable* data from other sources?

Yes ☐ No ☐

Section E: Validation/verification

23. Does this protocol describe a purely observational study using CPRD data (this may include the review of anonymised free text)?

Yes* ☒ No** ☐

** Yes: If you will be using data obtained from the CPRD Group, this study does not require separate ethics approval from an NHS Research Ethics Committee.*
*** No: You may need to seek separate ethics approval from an NHS Research Ethics Committee for this study. The ISAC will provide advice on whether this may be needed.*

24. Does this study require anonymised free text?

Yes* ☐ No ☒

**Please note that work involving free text can only be performed on the July 2013 CPRD GOLD database build or earlier versions. CPRD can provide further advice on the use of anonymised free text.*

25. Does this protocol involve requesting any additional information from GPs?

Yes* ☐ No ☒

** Please indicate what will be required:*

Completion of questionnaires by the GPΨ	Yes ☐	No ☐
Provision of anonymised records (e.g. hospital discharge summaries)	Yes ☐	No ☐
Other (please describe)		

Ψ Any questionnaire for completion by GPs or other health care professional must be approved by ISAC before circulation for completion.

For peer review only - http://bmjopen.bmj.com/site/about/guidelines.xhtml
For peer review only

**26. Does this study require contact with patients in order for them to complete a questionnaire?**<br><br>Yes* ☐ No ☒<br><br>**Please note that any questionnaire for completion by patients must be approved by ISAC before circulation for completion.*
**27. Does this study require contact with patients in order to collect a sample?**<br><br>Yes* ☐ No ☒<br><br>** Please state what will be collected:*
**Section F: Signatures**
**28. Signature from the Chief Investigator**<br><br>I confirm that the above information is to the best of my knowledge accurate, and I have read and understood the guidance to applicants.<br><br>Name: Ed Wilson Date: 25/05/16 E. signature (type name): ED WILSON

For peer review only

For peer review only - http://bmjopen.bmj.com/site/about/guidelines.xhtml

PROTOCOL INFORMATION

In order to help ensure that protocols submitted for review contain adequate information for protocol evaluation, ISAC have produced guidance on the content of protocols for research using CPRD data. This guidance is available on the CPRD website (www.cprd.com/ISAC). All protocols using CPRD data which are submitted for review by ISAC must contain information on all the areas detailed below. If a specific area required by ISAC is not applicable to your protocol, please provide the justification underneath the relevant heading.

The protocol section (next page) has pre-defined headings and the protocol must be written using these headings. Additional headings are not acceptable; however, supplementary information may be placed in one or more of the appendices providing this information is essential and an appropriate reference to it is made within the protocol. Unless very short, codes lists should be placed in an Appendix. Applications will be regarded as invalid and returned to the applicant if any of the headings below are missing or if additional sections are included.

Please note that ISAC will not consider any application where the protocol exceeds 12 pages (excluding sections A-F of the application form and annexes). Annexes should be kept to a minimum and contain only vital information that could not be provided in the main protocol section. A font-size of at least 12 should be used. Protocols not exceeding 15 pages would be acceptable if ISAC has required a resubmission where additional information is requested.

Please note, your protocol will not be reviewed by ISAC if it falls short of the above requirements. You are advised to speak to the Secretariat if you have any queries.

Voluntary registration of ISAC approved studies:

Epidemiological studies are increasingly being included in registries of research around the world, including those primarily set up for clinical trials. To increase awareness amongst researchers of ongoing research, ISAC encourages voluntary registration of epidemiological research conducted using MHRA databases. This will not replace information on ISAC approved protocols that may be published on the CPRD website. It is for the applicant to determine the most appropriate registry for their study. Please inform the ISAC secretariat that you have registered a protocol and provide the location.

For peer review only - http://bmjopen.bmj.com/site/about/guidelines.xhtml
For peer review only

Protocol Section

The following headings **must** be used to form the basis of the protocol. Pages should be numbered. All abbreviations must be defined on first use.

A. Lay Summary (Max. 200 words)

Please provide a succinct overview of your proposed research in plain English i.e. non-technical language. This should cover the background, purpose of the study and the potential importance of the findings. References and abbreviations should be avoided. If you have ticked the "other" box in response to question 4 on the application form, up to an additional 100 words should be used to describe the benefit to public health expected from the study.

In the NHS, general practitioners (GPs) have been encouraged to issue prescriptions of shorter duration (e.g., 28 days), to reduce drug expenditure and wastage. There is, however, the potential for shorter prescriptions to increase costs through increased GP workload and dispensing fees. Currently, the consequences of longer and shorter prescriptions for patients with chronic diseases are unknown and need to be assessed. The purpose of this study is to determine the if there are differences in the costs related to drug wastage, dispensing fees and prescriber time as a result of early refills and treatment switches for prescriptions issued as either a 28-day or 3-month supply in five selected case study scenarios representing common chronic conditions. This study will provide important information to the Department of Health in understanding the impact that encouraging GPs to issue shorter supplies of drugs has had on drug expenditure and drug wastage and additionally, help inform future prescribing policies.

B. Technical Summary (Max. 200 words)

Please provide a succinct overview of the objectives, methods and data analysis for the proposed research. Avoid the use of references in this section.

The aim of this study is to estimate the differences in the costs of drug wastage, dispensing fees and prescriber time as a result of early refills and treatment switches in patients receiving medications as either 28-day or 3-month supplies for a number of common chronic diseases. A retrospective cohort analysis will be conducted using data from a random sample of 50,000 patients for five case study conditions derived from all adult patients receiving at least one prescription relevant to the respective condition during the 10-year period between 2004 and 2014. The volume of wastage from early refills and treatment switches (defined as a repeat prescription or new prescription for a drug commonly prescribed for the same condition being issued prior to the expiry of the previously prescribed quantity) will be estimated. Unit costs from standard sources will be applied to estimate the cost of wastage and dispensing for a common price year. The cost of health professional time to issue the prescription will also be added. Changes in drug wastage and dispensing fees will then be estimated had all prescriptions been for 28 days rather than the observed length.

C. Objectives, Specific Aims and Rationale

Please include:
(i) The broad research objectives
(ii) The specific aims; any hypotheses to be tested should be stated here.

For peer review only - http://bmjopen.bmj.com/site/about/guidelines.xhtml

(iii) An explanation of how achievement of the specific aims will further the research objectives

The broad research objectives of the entire research project (note that the proposed study within this application is only one component of a larger NIHR-funded HTA project) are to assess whether shorter (28 day) or longer (3 month) prescription lengths have an impact on medication wastage, dispensing costs and health professional prescriber time.

The aims of the component of the study for which we require CPRD data are to investigate the patterns of treatment switching and early refills over a 10-year period in order to estimate differences in the cost of drug wastage, dispensing fees and health professional prescriber time for 28-day and 3-month prescription lengths.

The results of the study will provide evidence to guide policy on the optimal choice of prescription length based on the potential economic implications of different policy scenarios.

D. Background

Please provide a succinct review of the relevant background literature with references so as to explain the purpose of the study. Please ensure that you refer to any previous research in CPRD that is related, providing published references and, when known, the ISAC Protocol Number

In an effort to reduce expenditure on, and wastage of, drugs some commissioners have encouraged GPs to issue shorter prescriptions, typically 28 days in length.[NHS Cambridgeshire, 2009; NHS Dorset Clinical Commissioning Group, 2013] The rationale being, to strike a balance between patient convenience, good medical practice and drug wastage. It has been estimated that between £100 million and £300 million worth of prescriptions dispensed in the community was wasted in 2007 and 2009.[Trueman P et al., 2010] Some evidence suggests that this wastage could be reduced if prescriptions were limited to a 28 day supply.[Hawksworth GM et al., 1996]

Shorter prescriptions, however, may increase the costs to the healthcare system through increased GP workload and dispensing costs to pharmacists. Recent evidence suggested that dispensing fees, as a result of increased numbers of shorter prescriptions, cost the NHS approximately £150 million in 2009.[Wilson PM et al., 2013] If all 842.5 million prescription items dispensed in the community in England in 2008 had been 28-day repeats, dispensing fees would have been 50% higher (£700 million increase on £1.5 billion current expenditure).[White KG et al., 2010] This same conclusion followed from a simulation model published in 2004 comparing 100-day with 34-day supplies in a US Medicaid setting:[Domino ME et al., 2004] shorter prescription lengths were associated with a reduction in drug wastage of 5-14%. However, increases in dispensing fees more than exceeded this decrease in drug wastage.

Given the disparity and lack of evidence from the perspective of the NHS in the UK, it is clear that an analysis is required to assess the impact of prescription length on costs to health services in terms of wastage, dispensing fees and health professional prescriber time.

E. Study Type

For peer review only - http://bmjopen.bmj.com/site/about/guidelines.xhtml

Specify whether the study will be primarily descriptive, exploratory, hypothesis testing or a methodological piece of research.

Descriptive analysis.

F. Study Design

Describe the overall research design (for example, case-control, cohort) and reasons for choosing the proposed study design.

This study will be a retrospective cohort study of a random sample of 50,000 patients for each of the five case study conditions (see section K) derived from all adult (≥18 years old) patients receiving at least one prescription relevant to the respective condition (see Appendix 1) during the 10-year period between January 1, 2004 and December 31, 2014. The 10-year study period has been chosen to ensure a sufficient number of treatment switches (specifically switches between drugs that are in the same class or different classes, but therapeutically related) are observed as these may happen relatively infrequently over the course of treating some chronic diseases. Prescribing data over the 10-year period will be studied. Descriptive analyses of trends in treatment switching and early refills will be carried out for each annual period and 10-years overall.

G. Sample Size

Please provide an estimate of sample size, and, where possible, a formal power calculation. An estimate of the expected number of patients available in the CPRD database should normally be included.

A random sample of 50,000 patients for each of the five case study conditions (see section K) derived from all adult (≥18 years old) patients receiving at least one prescription relevant to the respective condition (see Appendix 1) during the 10-year period between 2004 and 2014 will be included. If we assume on average five years of follow up are available for a patient in CPRD [Herrett E et al., 2015] and that patients may be issued the prescriptions of interest for half that time (note this may be different depending on the condition of interest, but has been used as a lower limit) and that patients are likely to receive between 4 and 12 prescriptions per year (based on dispensing of either a one month or three month supply) than overall, patients in CPRD are likely to have between 10 and 30 prescriptions related to the conditions of interest. A random sample of 50,000 patients would result in roughly 500,000 to 1.5 million prescriptions in total. Given previously reported annual proportions of treatment switches for angiotensin-converting enzyme inhibitors (2.6%), sulfonylureas (0.8%) and selective serotonin reuptake inhibitors (1.0%) [Domino ME et al. 2004] and if we look to assess the number of switches each year over our 10-year study period, assuming the number of prescriptions is equally spread over the 10 years (~42,000/year) for a sample of 50,000 (lower limit 500,000 prescriptions in total) we would expect to detect these proportions of switches with acceptable precision [0.01 95% CI (0.0090705, 0.0109981) and 0.03 95% CI (0.0283892, 0.0316762)].

H. Data Linkage Required (if applicable)

Please provide a synopsis of the purpose(s) for which the each of the linkages requested in section 18 of the application form is required.

Not applicable.

For peer review only - http://bmjopen.bmj.com/site/about/guidelines.xhtml

I. Study Population

Define the source and study population, in terms of persons, place, time period, and listing the criteria which will be used to select the study population from the CPRD, i.e any inclusion or exclusion criteria. Please make clear any restrictions imposed by the use of linked datasets.

A random sample of 50,000 patients for each of the five case study conditions (see section K) derived from all adult (≥18 years old) patients receiving at least one prescription relevant to the respective condition (see Appendix 1) during the 10-year period between January 1, 2004 and December 31, 2014 will be included.

J. Selection of comparison group(s) or controls

Describe the criteria for eligibility and the procedure for control selection.

Not applicable.

K. Exposures, Outcomes and Covariates

For exposures and outcomes operational definitions (or procedures for developing them) must be provided, supported by preliminary code lists placed in an Annex. A comprehensive list of covariates should also be provided for any study which is not purely descriptive.

Five case study conditions were selected based on their frequency of occurrence within the population and the potential for a variety of expected frequencies in prescription changes over the course of treatment for each condition.

A list of medications routinely prescribed for the selected case study conditions was identified by review of appropriate clinical guidelines and consultation with clinical colleagues.

The five case study conditions are:

1) glucose control in type II diabetes (patients receiving at least one prescription for an anti-diabetic drug listed under 'BNF 6.1.2 Antidiabetic drugs');

2) primary prevention of hypertension in type II diabetes (in addition to receiving an anti-diabetic drug as defined in (1), patients receiving at least one prescription for a medication used for the primary prevention of hypertension in type II diabetes patients, including angiotensin-converting enzyme inhibitors, angiotensin II receptor antagonists, calcium-channel blockers and thiazide-like diuretics);

3) primary prevention of hyperlipidaemia in type II diabetes (in addition to receiving an anti-diabetic drug as defined in (1), patients receiving at least one prescription for a statin used for the primary prevention of hyperlipidaemia in type II diabetes patients;

4) secondary prevention of myocardial infraction (in addition to receiving concurrent prescriptions for a angiotensin-converting enzyme inhibitor, antiplatelet and statin for at least one year in duration, patients may also receive prescriptions for beta-

For peer review only - http://bmjopen.bmj.com/site/about/guidelines.xhtml

adrenoceptor blockers, calcium-channel blockers, oral anticoagulants and aldosterone antagonists);

5) and depression (patients receiving at least one prescription for an anti-depressant drug listed under 'BNF 4.3 Antidepressant drugs').

Preliminary product code lists of potentially prescribed medications for each of the five case study conditions are provided in Appendix 1.

L. Data/ Statistical analysis

This section should cover both the analytic methods and also the analyses which are to be performed to meet all the specific aims listed earlier. It is important to ensure that this section is clear and specific about any comparisons which will be made.

For each of the five case study conditions the associated product codes listed in Appendix I will first be reviewed to create groups of similar products, where possible. Next, all possible substitutions between the available products will be mapped and will include:

1)substitutions between different dosages or formulations of the same drug substance (active ingredient);
2) substitutions between drugs within the same class (e.g., switch between two different statins);
3) and substitutions between drugs that are therapeutically related (e.g., switch from angiotensin-converting enzyme inhibitor to calcium-channel blocker)

We will then estimate the volume of medication wastage from early refills and treatment switches (defined as a repeat prescription or new prescription based on the mapped substitutions outlined above respectively, being issued prior to the expiry of the previously prescribed quantity). This can be estimated by dividing the total quantity entered by the GP for the prescribed product (qty) by the numeric daily dose prescribed for the event (ndd) and comparing this to the difference in the two dates associated with the events, as entered by the GP (eventdate). A threshold of one year after the initial prescription in a particular series will be used to estimate wastage for early refills (i.e., repeat prescriptions), in that any product prescribed over and above the expected quantity to be consumed within the one year time period will be considered waste. This is to account for the fact that some patients may fill their prescriptions before their existing supply is exhausted, but still consume all of the previous prescription before using the new supply. Therefore, if we assumed wastage for all early refills we may be overestimating the impact.

In contrast, for treatment switches we will assume any additional product not consumed before the switch date will be considered waste. There are, however, two exceptions: 1) prescriptions issued on the same day for drugs in the same class (e.g., two different statins) will be considered prescriber error and drop from the analysis as it is unlikely that prescriptions for drugs in the same class would be issued on the same day; and 2) to differentiate between add-ons and switches, particularly for therapeutically related drugs we will only define an overlap of prescriptions dates as wastage due to a treatment switch if there is not another prescription issued for the original product within a three month time period. The three month threshold has been chosen to ensure prescriptions issued for both one and

For peer review only - http://bmjopen.bmj.com/site/about/guidelines.xhtml

three month periods are captured. The three month threshold will also be altered in sensitivity analysis to test the robustness of this assumption.

Alternatively, we may chose not to differentiate between actual medication wastage due to switches and the augmentation of medication through add-ons as well as count any overlapping dates as wastage for early refills. Under this scenario we may overstate the amount of wastage that occurred, but this can be considered a conservative assumption, as it puts an upper bound on the savings that would occur if premature medication switches could be eliminated entirely.

The cost of wastage can then be estimated by applying net ingredient costs (NICs) obtained from national general practice prescribing data provided by the Health & Social Care Information Centre for the respective prescription to the estimated quantity of waste. BNF codes will be used to link the NICs from the general prescribing data to CPRD. Since BNF codes from CPRD are limited to only a 7 digit numeric code representing chapter, section, paragraph and subparagraph (i.e., does not provide 8 digit alpha/numeric code representing the drug substance and specific formulation) it is necessary to make some additional assumptions to link the datasets. First, total NIC will be divided by total number of items prescribed and dispensed for each product listed to obtain a NIC per item. A weighted average using total quantities of all the NICs per item for a particular BNF paragraph will be calculated. For example, BNF code 0403010 in CPRD represents Chapter 4 “Central nervous system”, Section 3 “Antidepressant drugs”, Paragraph 1 “Tricyclic and related antidepressant drugs” and Subparagraph 0, which means the BNF does not extend to the subparagraph level in this case. Therefore a weighted average of all the NICs in Paragraph 1 “Tricyclic and related antidepressant drugs” will be applied to any drug falling in this category (e.g., amitriptyline, clomipramine, dosulepin, doxepin, imipramine, etc.).

Based on the number of prescriptions, dispensing fees related to each prescription from the Drug Tariff and the estimated cost of health professional prescriber time based on the literature can be added to the cost of wastage to determine the total cost from a NHS perspective. A targeted literature review will be designed to determine the time involved for a health professional to issue a prescription. Note this may be different depending on the type of health professional (e.g., general practitioner versus nurse), but this will be tested in sensitivity analysis. Hourly costs related to the health professionals’ time, derived from the PSSRU’s Unit Costs of Health & Social Care will then be applied.[Curtis L and Burns A, 2015]

Scenario analysis will then be conducted, estimating changes in drug wastage and dispensing fees if all prescriptions had been for 28 days rather than the observed length. Appropriate sensitivity analyses will also be conducted, for example, around the cost of health professional prescriber time required to issue a repeat prescription.

M. Plan for addressing confounding

Purely descriptive studies are exempt from this requirement. All other studies should here provide some discussion of what they are doing in the design and/or analysis to control for confounding.

Not applicable.

N. Plan for addressing missing data

For peer review only - http://bmjopen.bmj.com/site/about/guidelines.xhtml

For peer review only

The potential for missing data should be identified and how it will be addressed discussed here.

Missing data in the CPRD therapy file should not be a major issue. Our analysis does, however, rely on the use of the numeric daily dose (ndd) variable. As this variable is derived using a CPRD algorithm on common dosage strings there is the potential for it to be equal to zero in cases of a non-numeric textid (e.g., if the textid refers to say "apply as needed"). This type of textid is unlikely for the medications chosen to be included in our analysis and therefore our analysis will be limited to only those observations with a complete case (i.e., ndd is not missing or equal to zero and quantity is not missing). This seems to be acceptable as this approach has been employed in other similar CPRD studies using these two variables.[Brodie MJ et al., 2016 and Francis NA et al., 2016]

O. Limitations of the study design, data sources and analytical methods

The general limitations of the databases and observational research are well-known. Specific consideration of the potential impact of such limitations should be provided in the context of the proposed study.

The key limitations specific to this protocol are as follows:

1. To define three of the five case study conditions (see section K; conditions 2, 3 and 4) based only on the available prescription data from CPRD it was necessary to make assumptions regarding the population's composition. For example, to define a population receiving medication for the secondary prevention myocardial infraction it was necessary to assume (based on clinical guidelines) that patients receiving a angiotensin-converting enzyme inhibitor, antiplatelet and statin for at least one year in duration had previously had a myocardial infraction.
2. From the data we will not be able to differentiate between repeat prescriptions and a number of acute prescriptions. Therefore to avoid overestimating wastage we have proposed to use a threshold of one year after the initial prescription in a particular series to estimate wastage for early refills (i.e., repeat prescriptions), in that any product prescribed over and above the expected quantity to be consumed within the one year time period will be considered waste.
3. Five case study conditions were purposively rather than randomly selected to represent the impact of medication refill and switching behaviour, but they may not be representative of prescribing behaviour in other chronic conditions. The conditions do, however, represent some of the most common chronic conditions treated with prescribed medications.
4. Our estimates of drug wastage will not account for imperfect adherence and therefore might represent an underestimate of the true quantity and cost of medication wastage. However, an additional aspect of this project (being conducted by other colleagues under the same NIHR HTA proposal) will attempt to quantify the impact of imperfect adherence for both long and short prescription lengths.
5. NICs used to estimate the cost of wastage are the prices listed on the Drug Tariff or if not on the tariff, the list prices published by the manufacturer. NICs do not include any discounts that may be applied. NICs also do not include any adjustment for income obtained where a prescription charge is paid at the time the prescription is dispensed or where the patient has purchased a pre-payment certificate. However, NICs are the only linkable source of prescription drug unit for large datasets like CPRD.

For peer review only - http://bmjopen.bmj.com/site/about/guidelines.xhtml

6. BNF codes will be used to link the NICs from the general prescribing data to CPRD. Since BNF codes from CPRD are limited to only a 7 digit numeric code representing chapter, section, paragraph and subparagraph (i.e., does not provide 8 digit alpha/numeric code representing the drug substance and specific formulation) it is necessary to make some additional assumptions to link the datasets. First, total NIC will be divided by total number of items prescribed and dispensed for each product listed to obtain a NIC per item. A weighted average using total quantities of all the NICs per item for a particular BNF subparagraph will be calculated.
7. A main limitation of CPRD prescription data is that it does not indicate whether or not a medication has been dispensed or whether patients took their prescribed medications as recommended (i.e., it only indicate when a prescription has been issued). Therefore, our estimates may have overstated the amount of wastage that actually occurred. This, however, is a conservative assumption and can be seen as an upper bound on the savings that would occur if drug wastage from premature medication switches could be eliminated entirely.

P. Patient or user group involvement (if applicable)

Please indicate whether you have or intend to involve patient groups in your study. Such involvement is encouraged by ISAC and required for studies which directly involve patients.

In preparation for this proposal, we sent an outline of our proposed research to members of INsPIRE, a patient and public involvement panel for Bedfordshire and Cambridgeshire. Email comments were sent back from seven panel members. Five of the members stressed the importance of this research and six members maintained that three month prescriptions were preferable to 28 day prescriptions for chronic conditions. However, one member cautioned that 28 day prescriptions may be suitable for 'concern medications', such as sleeping pills. Six of the respondents mentioned the additional cost of shorter duration prescriptions, which they described in terms of drug wastage, patient time, GP time and prescription fees. Two members also mentioned the importance of synchronisation of prescriptions for patients with multiple co-morbidities. Finally, two members stressed the importance of focusing on individual patient needs when prescribing medications. During the writing of the proposal, we have taken these views into account.

Patients and the public were involved in the design of the research and will be involved in the dissemination of research findings.

Q. Plans for disseminating and communicating study results, including the presence or absence of any restrictions on the extent and timing of publication

ISAC expects most studies that it approves to be published in the scientific literature and considers it an ethical obligation for any study with potential public health implications. In cases where multiple publications are likely to arise, a publication plan should be provided in this section.

The primary audience for the proposed research will be policy makers, those who manage and provide care for patients with long-term stable chronic conditions (i.e., general practitioners and pharmacists), as well as patient groups with stable, chronic conditions who require regular repeat prescriptions. In addition to a HTA monograph, we plan to publish the findings in an academic peer-reviewed journal and present the findings at relevant academic

For peer review only - http://bmjopen.bmj.com/site/about/guidelines.xhtml

conferences. Our patients and public involvement members will be asked to assist in the production of a short summary for non-technical audiences.

R. References

Please provide a numbered list of references at the end of the protocol.

Brodie MJ, Chung S, Wade A, Quelen C, Guiraud-Diawara A, François C, Verpillat P, Shen V, Isojarvi Clobazam and clonazepam use in epilepsy: Results from a UK database incident user cohort study. Epilepsy Research 2016;123:68-74.

Curtis L, Burns A. Personal Social Services Research Unit – Unit Costs of Health & Social Care 2015. The University of Kent. Canterbury.

Domino ME, Olinick J, Sleath B, Leinwand S, Byrns PJ, Carey T. Restricting patients' medication supply to one month: Saving or wasting money? Am J Health-Syst Pharm 2004;61:1375-1379.

Francis NA, Hood K, Lyons R, Butler CC. Understanding flucloxacillin prescribing trends and treatment non-response in UK primary care: a Clinical Practice Research Datalink (CPRD) study. J Antimicrob Chemother 2016 Apr 18.

Hawksworth GM, Wright D, Chrystyn H. A detailed analysis of the day to day unwanted medicinal products returned to community pharmacies for disposal. Journal of social and administrative pharmacy 1996;13:215-222.

Herrett E, Gallagher AM, Bhaskaran K, Forbes H, mathur R, van Staa T, Smeeth L. Data Resource Profile: Clinical Practice Research Datalink (CPRD). International Journal of Epidemiology 2015;44(3):827-836.

NHS Cambridgeshire. Repeat Medication for 28 Days. 2009

NHS Dorset Clinical Commissioning Group. Medicines Code Chapter 15: Policy for Repeat Prescribing and Medication Review. 2013

Trueman P, Lowson K, Blighe A et al. Evaluation of the Scale, Causes and Costs of Waste Medicines. York & London: York Health Economics Consortium & The School of Pharmacy, University of London, 2010.

White KG. UK interventions to control medicines wastage: a critical review. Int J Pharm Pract 2010;18(3):131-40.

Wilson PM, Kataria N, McNeilly E. patient and carer experience of obtaining regular prescribed medication for chronic disease in the English National Health Service: a qualitative study. BMC health services research 2013;13(1):192.

Appendices

Appendices should be used for essential supporting information only (e.g. code-lists) and they must be cited within the body of the protocol.

For peer review only - http://bmjopen.bmj.com/site/about/guidelines.xhtml

Please see accompanying document:

- Appendix 1: Preliminary product codes lists for each of the five case study conditions of interest (Excel file)

For peer review only

For peer review only - http://bmjopen.bmj.com/site/about/guidelines.xhtml

Appendix VIII - Comparison of medication wastage over 11-year period 2004-2014

	Proportion of days' supply wasted % (95% CI)		Mean number of days' supply wasted days (95% CI)		Mean cost of wastage per prescription 2015 £ (95% CI)	
	<60 days	≥60 days	<60 days	≥60 days	<60 days	≥60 days
Glucose control with oral drug therapy in T2DM	2.658 (2.641-2.675	4.920 (4.822-5.018)	0.859 (0.853-0.865)	4.962 (4.044-5.880)	0.329 (0.325-0.333)	1.370 (1.104-1.636)
Hypertension in T2DM	3.762 (3.747-3.777)	5.011 (4.935-5.087)	1.232 (1.227-1.237)	6.979 (5.956-8.002)	0.095 (0.094-0.096)	0.437 (0.389-0.486)
Lipid management in T2DM	1.652 (2.640-1.665)	4.071 (3.966-4.177)	0.628 (0.623-0.633)	16.211 (12.979-19.443)	0.048 (0.048-0.049)	1.426 (1.132-1.720)
Secondary prevention of myocardial infraction	3.325 (3.315-3.335)	3.663 (3.612-3.714)	0.956 (0.953-0.959)	6.557 (5.761-7.353)	0.066 (0.066-0.066)	0.510 (0.370-0.649)
Depression	6.340 (6.157-6.385)	3.663 (3.535-3.792)	1.866 (1.852-1.881)	2.695 (2.592-2.797)	0.207 (0.203-0.212)	0.429 (0.977-0.480)

CI – confidence interval; T2DM – type II diabetes mellitus

For peer review only

Appendix IX - Comparison of the mean cost of medication wastage per prescription each year from 2004 to 2014 (2015 £)

	Glucose control with oral drug therapy in T2DM 2015 £ (95% CI)		Hypertension in T2DM 2015 £ (95% CI)		Lipid management in T2DM 2015 £ (95% CI)		Secondary prevention of myocardial infraction 2015 £ (95% CI)		Depression 2015 £ (95% CI)	
**Year**	**<60 days**	**≥60 days**	**<60 days**	**≥60 days**	**<60 days**	**≥60 days**	**<60 days**	**≥60 days**	**<60 days**	**≥60 days**
2004	0.381 (0.364-0.397)	1.813 (0.574-3.052)	0.127 (0.122-0.131)	0.462 (0.351-0.573)	0.068 (0.065-0.070)	1.511 (0.599-2.422)	0.085 (0.084-0.087)	0.376 (0.175-0.578)	0.247 (0.227-0.266)	0.428 (0.308-0.548)
2005	0.423 (0.405-0.441)	1.331 (1.080-1.582)	0.123 (0.118-0.127)	0.525 (0.386-0.663)	0.055 (0.053-0.058)	2.363 (1.118-3.607)	0.082 (0.081-0.084)	0.521 (0.321-0.721)	0.221 (0.204-0.238)	0.452 (0.296-0.608)
2006	0.418 (0.400-0.435)	1.045 (0.906-1.185)	0.111 (0.107-0.114)	0.428 (0.328-0.529)	0.057 (0.055-0.060)	2.274 (1.059-3.488)	0.079 (0.077-0.080)	1.277 (0.132-2.421)	0.222 (0.205-0.239)	0.579 (0.359-0.800)
2007	0.387 (0.370-0.403)	1.110 (0.956-1.264)	0.101 (0.098-0.104)	0.467 (0.325-0.608)	0.050 (0.048-0.052)	1.533 (0.557-2.509)	0.072 (0.070-0.073)	0.400 (0.0240-0.559)	0.222 (0.205-0.239)	0.376 (0.247-0.505)
2008	0.343 (0.329-0.357)	1.086 (0.922-1.249)	0.096 (0.093-0.099)	0.530 (0.314-0.746)	0.045 (0.043-0.048)	1.663 (0.600-2.726)	0.065 (0.065-0.067)	0.457 (0.259-0.654)	0.207 (0.192-0.221)	0.359 (0.227-0.491)
2009	0.299 (0.287-0.311)	1.074 (0.849-1.298)	0.091 (0.088-0.093)	0.472 (0.282-0.662)	0.048 (0.046-0.051)	1.717 (0.638-2.796)	0.063 (0.062-0.064)	0.528 (0.302-0.754)	0.206 (0.191-0.220)	0.421 (0.243-0.599)
2010	0.330 (0.317-0.342)	1.396 (0.744-2.048)	0.085 (0.082-0.087)	0.379 (0.213-0.545)	0.046 (0.043-0.048)	0.991 (0.245-1.738)	0.061 (0.060-0.062)	0.477 (0.253-0.701)	0.183 (0.171-0.195)	0.366 (0.216-0.516)
2011	0.263 (0.254-0.272)	3.139 (0.659-5.620)	0.082 (0.080-0.085)	0.477 (0.259-0.695)	0.042 (0.040-0.044)	0.879 (0.154-1.605)	0.057 (0.056-0.058)	0.409 (0.208-0.611)	0.173 (0.162-0.183)	0.383 (0.239-0.528)
2012	0.251 (0.243-0.260)	0.932 (0.820-1.043)	0.078 (0.075-0.080)	0.402 (0.208-0.596)	0.045 (0.043-0.047)	0.740 (0.135-1.346)	0.056 (0.055-0.057)	0.354 (0.192-0.516)	0.182 (0.169-0.195)	0.350 (0.171-0.528)
2013	0.268 (0.258-0.277)	0.871 (0.758-0.984)	0.074 (0.072-0.076)	0.232 (0.189-0.275)	0.038 (0.036-0.039)	0.448 (0.063-0.834)	0.053 (0.052-0.054)	0.200 (0.131-0.268)	0.199 (0.186-0.213)	0.436 (0.189-0.683)
2014	0.301 (0.290-0.311)	1.168 (1.018-1.318)	0.079 (0.076-0.082)	0.271 (0.202-0.340)	0.047 (0.045-0.050)	0.796 (0.102-1.489)	0.054 (0.053-0.056)	0.188 (0.103-0.273)	0.233 (0.217-0.249)	0.593 (0.374-0.812)

NS – not significant at $p<0.05$ level; T2DM – type II diabetes mellitus

For peer review only

Appendix X – Differences in standardised (120 day) total unnecessary costs for short and long prescription length under various scenarios (2015 £)

	**Values tested**		**Total unnecessary cost standardised to 120 days**		**Difference (Cost savings with ≥60 day)**
Scenarios	**<60 days**	**≥60 days**	**<60 days**	**≥60 days**	
**Initial glucose control in type II diabetes**					
Base case	-	-	13.24	4.86	(8.38)
Nurse prescriber time cost, not GP	2.28	2.29	10.13	4.19	(5.94)
No prescriber time cost	0	0	3.76	2.85	(0.91)
Addition of prescription charge (loss in NHS revenue)	0.84	0.84	10.89	4.37	(6.52)
50% decrease quantity wasted, days	0.43	2.48	12.65	3.74	(8.91)
50% increase quantity wasted, days	1.29	7.44	13.84	5.98	(7.86)
50% decreased cost of drug per day	0.18	0.14	12.65	3.74	(8.91)
50% increase cost of drug per day	0.55	0.43	13.84	5.98	(7.86)
50% decrease dispensing fee	0.46	0.52	11.96	4.55	(7.41)
50% increase dispensing fee	1.38	1.57	14.53	5.16	(9.37)
50% decrease prescriber time cost	1.70	1.72	8.50	3.85	(4.65)
50% increase prescriber time cost	5.09	5.16	17.99	5.86	(12.13)
**Hypertension in type II diabetes**					
Base case	-	-	12.32	2.50	(9.82)
Nurse prescriber time cost, not GP	2.31	2.32	9.04	2.03	(7.01)
No prescriber time cost	0	0	2.81	1.15	(1.66)
Addition of prescription charge (loss in NHS revenue)	0.84	0.84	10.06	2.18	(7.88)
50% decrease quantity wasted, days	0.62	3.49	12.13	2.10	(10.03)
50% increase quantity wasted, days	1.85	10.47	12.52	2.89	(9.63)
50% decreased cost of drug per day	0.042	0.041	12.13	2.10	(10.03)
50% increase cost of drug per day	0.13	0.12	12.52	2.89	(9.63)

For peer review only - http://bmjopen.bmj.com/site/about/guidelines.xhtml

	Values tested		Total unnecessary cost standardised to 120 days		Difference (Cost savings with ≥60 day)
Scenarios	<60 days	≥60 days	<60 days	≥60 days	
50% decrease dispensing fee	0.45	0.46	11.11	2.32	(8.79)
50% increase dispensing fee	1.35	1.39	13.54	2.67	(10.87)
50% decrease prescriber time cost	1.77	1.77	7.57	1.82	(5.75)
50% increase prescriber time cost	5.30	5.32	17.08	3.17	(13.91)
**Hyperlipidaemia in type II diabetes**					
Base case	-	-	10.95	1.54	(9.41)
Nurse prescriber time cost, not GP	2.21	2.22	8.54	1.56	(6.98)
No prescriber time cost	0	0	2.64	1.61	(1.03)
Addition of prescription charge (loss in NHS revenue)	0.84	0.84	8.71	1.56	(7.15)
50% decrease quantity wasted, days	0.31	8.11	10.83	0.73	(10.10)
50% increase quantity wasted, days	0.94	24.32	11.07	2.36	(8.71)
50% decreased cost of drug per day	0.052	0.052	10.83	0.73	(10.10)
50% increase cost of drug per day	0.16	0.15	11.07	2.36	(8.71)
50% decrease dispensing fee	0.45	0.50	9.75	1.55	(8.20)
50% increase dispensing fee	1.35	1.50	12.15	1.53	(10.62)
50% decrease prescriber time cost	1.56	1.57	6.79	1.58	(5.21)
50% increase prescriber time cost	4.68	4.72	15.11	1.51	(13.60)
**Secondary prevention of myocardial infraction**					
Base case	-	-	13.40	1.34	(12.06)
Nurse prescriber time cost, not GP	2.37	2.37	9.49	1.10	(8.39)
No prescriber time cost	0	0	2.81	0.69	(2.12)
Addition of prescription charge (loss in NHS revenue)	0.84	0.84	11.03	1.20	(9.83)
50% decrease quantity wasted, days	0.48	3.28	13.27	1.08	(12.19)
50% increase	1.43	9.84	13.54	1.60	(11.94)

For peer review only - http://bmjopen.bmj.com/site/about/guidelines.xhtml

	Values tested		Total unnecessary cost standardised to 120 days		Difference (Cost savings with ≥60 day)
Scenarios	**<60 days**	**≥60 days**	**<60 days**	**≥60 days**	
quantity wasted, days					
50% decreased cost of drug per day	0.037	0.034	13.27	1.08	(12.19)
50% increase cost of drug per day	0.11	0.10	13.54	1.60	(11.94)
50% decrease dispensing fee	0.45	0.48	12.13	1.26	(10.87)
50% increase dispensing fee	1.35	1.45	14.67	1.43	(13.24)
50% decrease prescriber time cost	1.88	1.89	8.11	1.02	(7.09)
50% increase prescriber time cost	5.63	5.66	18.70	1.67	(17.03)
**Depression**					
Base case	-	-	15.06	4.04	(11.02)
Nurse prescriber time cost, not GP	2.24	2.23	11.72	3.24	(8.48)
No prescriber time cost	0	0	4.16	1.35	(2.81)
Addition of prescription charge (loss in NHS revenue)	0.84	0.84	12.22	3.33	(8.89)
50% decrease quantity wasted, days	0.93	1.35	14.51	3.77	(10.74)
50% increase quantity wasted, days	2.80	4.04	15.61	4.31	(11.30)
50% decreased cost of drug per day	0.068	0.054	14.51	3.77	(10.74)
50% increase cost of drug per day	0.20	0.16	15.61	4.31	(11.30)
50% decrease dispensing fee	0.45	0.48	13.53	3.64	(9.89)
50% increase dispensing fee	1.36	1.44	16.59	4.45	(12.14)
50% decrease prescriber time cost	1.61	1.59	9.61	2.70	(6.91)
50% increase prescriber time cost	4.84	4.78	20.51	5.39	(15.12)

For peer review only

BMJ Open

A retrospective, multi-cohort analysis of the Clinical Practice Research Datalink (CPRD) to determine differences in the cost of medication wastage, dispensing fees and prescriber time of issuing either short (<60 day) or long (≥60 day) prescription lengths in primary care for common, chronic conditions in the United Kingdom

Journal:	*BMJ Open*
Manuscript ID	bmjopen-2017-019382.R1
Article Type:	Research
Date Submitted by the Author:	11-Oct-2017
Complete List of Authors:	Doble, Brett; University of Cambridge, Cambridge Centre for Health Services Research; University of Oxford, Nuffield Department of Population Health<br>Payne, Rupert; University of Cambridge, Cambridge Centre for Health Services Research; University of Bristol, Centre for Academic Primary Care<br>Harshfield, Amelia; University of Cambridge, Cambridge Centre for Health Services Research; University of Cambridge, Department of Public Health and Primary Care<br>Wilson, Edward; University of Cambridge, Cambridge Centre for Health Services Research
**Primary Subject Heading**:	Health economics
Secondary Subject Heading:	General practice / Family practice, Health services research, Health policy, Evidence based practice
Keywords:	PRIMARY CARE, Health policy < HEALTH SERVICES ADMINISTRATION & MANAGEMENT, HEALTH ECONOMICS, THERAPEUTICS

SCHOLARONE™
Manuscripts

A retrospective, multi-cohort analysis of the Clinical Practice Research Datalink (CPRD) to determine differences in the cost of medication wastage, dispensing fees and prescriber time of issuing either short (<60 day) or long (≥60 day) prescription lengths in primary care for common, chronic conditions in the United Kingdom

For peer review only

Brett Doble*[1,2], Rupert Payne[1,3], Amelia Harshfield[1,4], Edward C.F. Wilson[1]

[1]Cambridge Centre for Health Services Research, University of Cambridge, Cambridge, CB2 0SR, United Kingdom

[2]Health Economics Research Centre, Nuffield Department of Population Health, University of Oxford, Oxford, OX3 7LF, United Kingdom

[3]Centre for Academic Primary Care, Department of Population Health Sciences, Bristol Medical School, University of Bristol, Bristol, BS8 2PS, United Kingdom

[4]Department of Public Health and Primary Care, University of Cambridge, Cambridge, CB1 8RN, United Kingdom

*Brett Doble, Health Economics Research Centre, Nuffield Department of Population Health, University of Oxford, Oxford, OX3 7LF, United Kingdom. Tel: +44(0)1865617913; Email: brett.doble@dph.ox.ac.uk

Funding Sources: This research was supported by a grant from the National Institute for Health Research, Health Technology Assessment funding stream (Grant Reference: NIHR HTA 14/159/07). This article presents independent research funded by the National Institute for Health Research (NIHR). The views expressed are those of the authors and not necessarily those of the National Health Service, the National Institute for Health Research or the Department of Health.

Acknowledgements: This study is based on data from the Clinical Practice Research Datalink obtained under license from the UK Medicines and Healthcare Products Regulatory Agency. The interpretation and conclusions contained in this study are those of the authors alone. We would also like acknowledge the valuable feedback received from Céline Miani, Adam Martin, Josephine Exley, Catherine Meads and Sarah King during the conduct of this study.

Word Count: 3,993

For peer review only - http://bmjopen.bmj.com/site/about/guidelines.xhtml

ABSTRACT

Objectives: To investigate patterns of early repeat prescriptions and treatment switching over an 11-year period to estimate differences in the cost of medication wastage, dispensing fees and prescriber time for short (<60 days) and long (≥60 days) prescription lengths from the perspective of the National Health Service in the United Kingdom.

Setting: Retrospective, multiple cohort study of primary care prescriptions from the Clinical Practice Research Datalink.

Participants: Five random samples of 50,000 patients each prescribed oral drugs for (1) glucose control in type 2 diabetes mellitus (T2DM), (2) hypertension in T2DM, (3) statins (lipid management) in T2DM, (4) secondary prevention of myocardial infarction and (5) depression.

Primary and secondary outcome measures: The volume of medication wastage from early repeat prescriptions and three other types of treatment switches was quantified and costed. Dispensing fees and prescriber time were also determined. Total unnecessary costs (TUC, cost of medication wastage, dispensing fees, and prescriber time) associated with <60 day and ≥60 day prescriptions, standardised to a 120-day period, were then compared.

Results: Longer prescription lengths were associated with more medication waste per prescription. However, when including dispensing fees and prescriber time, longer prescription lengths resulted in lower TUC. This finding was consistent across all five cohorts. Savings ranged from £8.38 to £12.06 per prescription per 120 days if a single long prescription were issued instead of multiple short prescriptions. Prescriber time costs accounted for the largest component of TUC.

Conclusions: Shorter prescription lengths could potentially reduce medication wastage, but they may also increase dispensing fees and/or the time burden of issuing prescriptions.

Keywords: costs, fill quantity, medication wastage, prescribing policy, prescription length

For peer review only - http://bmjopen.bmj.com/site/about/guidelines.xhtml

ARTICLE SUMMARY

Strengths and limitations of this study

- Our analysis builds on existing methodological approaches to estimate the unnecessary costs associated with different prescription lengths, providing the only evidence available from the perspective of the NHS in the UK.
- Limitations of our study do risk biasing the results and the reported savings (£8.38 to £12.06 per prescription per 120 days) should therefore be interpreted with caution and considered upper limits.
- CPRD prescription data only indicates whether a prescription has been issued and not whether it was dispensed or taken as recommended, potentially resulting in an over- or under-estimate of the amount of wastage, depending on patient behaviour not captured in CPRD.
- The five case study conditions used in our study were purposively rather than randomly selected to represent the impact of repeat prescriptions and switching behaviour on wastage; they may not be representative of prescribing behaviour in other chronic conditions.
- Overlap of dates between prescriptions does not necessarily mean wastage has occurred and despite incorporating methods to account for this there is the possibility that our analysis approach could be overestimating the amount of medication wastage.

For peer review only - http://bmjopen.bmj.com/site/about/guidelines.xhtml

INTRODUCTION

Healthcare systems worldwide are increasingly faced with the challenge of constraining rising pharmaceutical expenditures.[1] One approach to addressing this problem is to ensure prescribed medication is used as efficiently as possible, minimising wastage. Wastage may occur when patients collect repeat prescriptions early, or when changes are made to patients' drug regimens. Intuitively, the more drugs a patient has in her/his possession at the time of a repeat prescription or regimen change, the higher the wastage. Therefore, limiting the quantity of medication through shorter prescription lengths could minimise wastage and help contain expenditure. However, the resulting higher frequency of prescriptions will have the unintended consequence of increasing transactions costs, specifically dispensing fees charged by pharmacists and healthcare professionals' time to issue them.

Several studies have examined the costs associated with issuing either long (three-month) or short (one-month) supplies of prescriptions.[2-7] In general, these concluded that shorter prescriptions were associated with lower wastage and hence reduced cost, but the increased transactions costs of shorter prescriptions more than offset these savings. These studies are all US based, which has very different healthcare systems from the UK, particularly with regards to the cost and dispensing of drugs. Therefore, the generalisability of these conclusions to the UK are questionable. Furthermore, none of the studies include healthcare professionals' time burden associated with issuing prescriptions.

In this study we estimate differences in the costs of medication wastage and transactions costs (in terms of dispensing fees and prescriber time) in patients receiving medications within the NHS in the UK as either short or long prescription lengths for five drugs/classes of drugs prescribed in primary care for common, chronic conditions.

For peer review only - http://bmjopen.bmj.com/site/about/guidelines.xhtml

METHODS

Overview

We undertook an analysis of Clinical Practice Research Datalink (CPRD)[8] prescription data to estimate the cost of medication wastage associated with shorter and longer prescription lengths for drugs used to treat five case study conditions. In order to estimate the net cost impact of shorter and longer prescription lengths, the cost of dispensing fees and prescribers' time to issue a prescription were also assessed.

Study design and inclusion criteria

This retrospective multi-cohort study evaluated medication wastage and its associated cost plus dispensing fees and the cost associated with issuing a prescription (i.e., a general practitioner (GP) completing the process of producing a prescription; note this does not include clinical decision-making time or administrative staff time) in five, condition-specific, random samples of 50,000 patients each, obtained from CPRD.

We derived the five samples from all adult patients (≥18 years old) receiving one or more prescriptions for at least one medication relevant to a case study of interest (Table 1) between January 1, 2004 and December 31, 2014. In line with other studies of CPRD data [9,10] inclusion was restricted to patients with complete data for two variables (numeric daily dose [ndd] and quantity [qty]) required to calculate the prescription duration. The five case studies were defined using unique lists of product codes (CPRD unique code for treatment selected by the GP). They were: 1) glucose control with oral drug therapy in type II diabetes mellitus (T2DM); 2) treatment of hypertension in T2DM; 3) treatment with statins (lipid management) in T2DM; 4) treatment for the secondary prevention of myocardial infarction (MI); and 5) treatment of depression.

For peer review only - http://bmjopen.bmj.com/site/about/guidelines.xhtml

These were selected for study based on the chronic nature and prevalence of the associated condition within the population, the potential for a variety of prescription changes over the course of treatment and the fact that medications used in their treatment have stable dosing once therapeutic effect has been achieved, making either short or long prescription clinically appropriate.[7] Definitions of the relevant prescriptions and product code lists of the potentially prescribed medications for each of the five case studies are provided in Table 1 and Appendix I respectively. Sample data counts are provided in Appendix II.

Treatment patterns evaluated

For each cohort, data for each patient were first ordered in sequence from earliest to latest prescription date. To identify treatment patterns three main variables were used: 1) product code (used to identify a unique dosage, formulation and brand (or generic version) of one particular drug); 2) drug substance (used to identify different dosages and/or formulations of the same drug chemical substance); and 3) drug class (used to identify drugs with different, but related chemical composition, with similar mechanisms of action based on their categorisation in the British National Formulary (BNF)). Four different prescription patterns in an individual's sequence of prescriptions were identified: 1) repeat prescriptions of the same product code; 2) substitutions between different dosages or formulations of the same drug substance; 3) substitutions between drugs that are in the same class; and 4) substitutions between drugs that have similar clinical indications from different classes. Prescriptions issued on the same day for drugs in the same class with different product codes were considered prescriber error and the duplicates were dropped from the analysis. The exception to this was for antiplatelet drugs in secondary prevention of MI, as it was assumed that two different antiplatelet drugs could be prescribed at the same time. In addition, prescriptions for medications with similar clinical indications from different classes issued on the same day

For peer review only - http://bmjopen.bmj.com/site/about/guidelines.xhtml

were not counted as a switch, but rather as an add-on to existing therapy or concomitant therapy (Appendix II).

Analysis of wastage

Wastage from early repeat prescriptions (pattern 1) was based on a cumulative excess supply built up over a period of 1 year. This avoided overestimation of wastage where a patient filled a prescription a few days early, but then finished their previous supply before starting the new one. In estimating wastage from switches (patterns 2-4), we adapted a previous approach [6] to differentiate between add-ons/concomitant therapy and actual switches. If the difference between the number of changes between medications with similar clinical indications from different classes and the number of unique drug classes within a rolling annual period was ≥1, then any overlap in prescription dates were considered to be an add-on rather than a switch. This is illustrated in Box 1. Similar constraints were also applied in three of the case studies (i.e., the glucose control in T2DM, treatment of hypertension in T2DM and secondary prevention of myocardial infraction cohorts) due to the potential for a number of the included therapies to be given concomitantly (Appendix III).

Costs

To estimate the costs of wastage, defined daily doses (DDD) associated with each drug substance code in the five cohorts were first obtained from the World Health Organisation's ATC/DDD Index 2016.[11] The Prescription Cost Analysis (PCA) 2015 which provides details of the quantity of individual doses and net ingredient costs (NICs) of all the prescriptions in England [12] was used to determine a NIC/quantity value of a specific strength of the medication associated with each drug substance code. This value was standardised using the associated DDD to obtain a cost per day for each drug substance code in all five of the cohorts. Details of these calculations are provided in Appendix IV.

For peer review only - http://bmjopen.bmj.com/site/about/guidelines.xhtml

Dispensing fees from the Drug Tariff (£0.90 per standard prescription and 2% of the cost per prescription [cost per day multiplied by prescription length] for prescriptions over £100) [13] and the estimated cost of physician or nurse time to issue a prescription were determined for each prescription. Time to issue a prescription was extracted from a targeted literature review (Appendix V). It should be noted that none of the identified studies reported times from a UK-specific primary care context. It was therefore necessary to prioritise the use of available evidence based on studies with the largest sample sizes and those studies reporting prescriber time for different types of prescriptions. Repeat prescriptions were assigned a shorter time compared to changes in dose/formulation, within drug classes and between drug classes (48.7 versus 61.2 seconds).[14] Per minute costs related to GPs' time (£3.80/minute) or a general practice nurse's time (£0.93/minute) were then applied.[15] All costs are reported in 2015 GBP.

Statistical analysis

Descriptive analyses of trends in treatment switching and early repeat prescriptions were used to assess medication wastage. The proportion of days' supply wasted, mean number of days' supply wasted and the mean costs of wastage per prescription were determined for two prescription lengths (<60 and ≥60 days, hereafter 'short' and 'long' prescriptions) over the 11-year period. Mean cost of wastage per prescription was reported for each of the four treatment patterns individually and for all treatment patterns combined for each annual period. Two-sample *t*-tests using groups (<60 and ≥60 days prescription lengths) assuming unequal variance were used to compare the differences between the <60 days and ≥60 days groups.

To determine and compare the total unnecessary costs (TUC, cost of medication wastage, dispensing fees, and prescriber time) associated with short and long prescription lengths, a model originally used by Walton et al.[5] was adapted and applied to the prescription

For peer review only - http://bmjopen.bmj.com/site/about/guidelines.xhtml

data from the five cohorts (Appendix VI), and the two equations below were used, where 'C' represents cost and 'Q' represent quantity. An example calculation is provided in Box 2.

1) $TUC_{<60}=(C_{wastage<60} + C_{dispensing<60} + C_{prescribertime<60}) \times (120/Q_{daysused<60}) - (C_{dispensing<60} + C_{prescribertime<60})$

2) $TUC_{\geq 60}=(C_{wastage\geq 60} + C_{dispensing\geq 60} + C_{prescribertime\geq 60}) \times (120/Q_{daysused\geq 60}) - (C_{dispensing\geq 60} + C_{prescribertime\geq 60})$

One-way sensitivity analyses were conducted to examine differences in TUC under a variety of different scenarios, including scenarios assuming nurses issued the prescription instead of a GP, excluding prescriber time costs, accounting for changes in NHS revenue from patient charges per prescription, +/- 50% mean days wasted, +/- 50% the mean cost of drugs per day, dispensing fees, and prescriber time.

All statistical analyses were performed using Stata/MP 13.1 (Stata Corp LP, College Station, Texas, USA). The protocol (16_117R) for this study was approved on June 21, 2016 by the Independent Scientific Advisory Committee (ISAC), the independent body that approves use of CPRD data (Appendix VII).

RESULTS

Overall cohort selection

The proportion of observations dropped from the full sample due to missing or observations equal to zero in either the ndd or qty variables ranged from 6% in both the lipid management and hypertension cohorts to 21% in the glucose control in T2DM cohort. The numbers of observations were further reduced after accounting for prescription error (Appendix II).

For peer review only - http://bmjopen.bmj.com/site/about/guidelines.xhtml

Medication wastage

Over the 11-year study period there was a statistically significant difference in the proportion of days' supply wasted, mean number of day's supplied wasted and the mean cost of wastage per prescription between the short and long prescriptions groups for all five of the case studies (Appendix VIII). The proportion of days' supply wasted was consistently larger for the long prescription group across all cohorts except depression where the short group had 6.3% of days' supply wasted compared to 3.7% in the longer group. The mean number of days' supply wasted was also consistently larger for the longer group, but the difference between the two prescription length groups was much smaller for the depression cohort in comparison to the other four cohorts.

Medication wastage by treatment pattern

In four of the five cohorts, mean cost of wastage per prescription was significantly higher with longer prescription lengths for all four treatment patterns (Table 2). The one exception was for the depression cohort where the mean cost of wastage per prescription for both dosage/formulation and within class treatment switches did not show statistically significant differences between the two prescription length groups. The repeat prescription treatment pattern consistently had the largest mean cost of wastage per prescription across the cohorts, particularly for the longer groups, except for the depression cohort. The lipid management cohort did not report any between class treatment switches as all medications included in the analysis were from the same class of statins.

Medication wastage over time

On an annual basis, mean cost of wastage per prescription was significantly higher in the longer prescription lengths for each study year, except 2012 and 2013 for depression (Appendix IX). In general, the magnitude of the mean costs remained relatively consistent over the study period, except for a few notable trends (Figure 1). In the glucose control in

For peer review only - http://bmjopen.bmj.com/site/about/guidelines.xhtml

T2DM cohort the mean costs for the longer group in years 2004 and 2011 were slightly larger (range £1.81 to £3.14) compared to the other nine annual means (range £0.87 to £1.40). In the hypertension cohort there was a slight trend of decreasing magnitude of the mean cost over the 11 years for the shorter group; a decrease in mean cost was limited to years 2013 and 2014 in the longer group. For both the lipid management and secondary prevention of myocardial infarction cohorts the magnitude of the mean costs remained relatively consistent over the 11 years for the shorter prescription length groups, whereas there was a slightly decreasing trend in the magnitude of the mean costs for the longer prescription length groups.

Differences in total unnecessary costs for short and long prescription lengths

TUC (wastage, dispensing fees and prescriber time) per 120-days was lower in the longer prescription group for all five cohorts (savings of £8.38 [glucose control in T2DM] to £12.06 [secondary prevention of MI] per prescription per 120 days if a single long prescription were issued instead of multiple short prescriptions, Appendix X). This roughly translates into savings of £25.14 to £36.18 per patient per year assuming patients would receive three prescriptions each with a 120-day supply instead of 12 prescriptions each with a 30-day supply.

Sensitivity analysis shows longer prescriptions remained cost saving compared with shorter prescriptions across all scenarios and ranges tested. The magnitude of the savings was lowest when prescriber time costs were excluded from the models (range £0.91 to £2.81 per prescription per 120 days) and reduced to a lesser extent when nurse prescriber time costs were used instead of physician's (range £5.94 to £8.48 per prescription per 120 days) and when loss of revenue to the NHS through a reduced number of prescription charges paid by patients was incorporated into the models (range £6.52 to £9.83 per prescription per 120 days). The other scenarios tested had relatively little impact on the magnitude of the savings,

For peer review only - http://bmjopen.bmj.com/site/about/guidelines.xhtml

with the exception of increases and decreases of 50% in the cost of prescriber time (Appendix X).

DISCUSSION

Summary of findings

Longer prescription lengths are associated with more medication wastage per prescription compared to shorter prescription lengths. However, after taking into account transaction costs, longer prescription lengths are associated with overall cost savings (lower TUC) compared with shorter ones. In all five cohorts, most prescriptions were for ≤30 days with relatively small proportions of patients having prescription lengths between 31 and 60 days (18%, 27%, 28%, 27% and 25% for the depression, T2DM, hypertension, lipid management and myocardial infarction cohorts respectively). Ninety five percent of prescriptions in the depression cohort were for <60 days. Some 39 million prescriptions are issued for antidepressants in the UK each year,[16] therefore, if the 95% issued as <60 day supplies were instead issued as longer ≥60 day prescriptions the total savings to the NHS could be as much as £408 million per year. Similarly, knowing 97.05% of statin prescriptions were issued as <60 day prescriptions from our CPRD analysis, the total savings to the NHS just in England could be as much as £563 million per year if the ~61.1 million[17] short statin prescriptions issued in 2015 for two statins (simvastatin and atorvastatin) were changed to longer prescriptions. However, it is critical to note that the majority of savings for both examples will not be cash releasing, but will be realised as savings in GP time, which could be used to increase primary care consultations with patients. Cash-releasing savings may come from reduced dispensing fees, for which we estimate an upper limit of £104 million and £62 million for antidepressants and the statins respectively. However, these cash savings will come at the expense of community pharmacies that may rely on dispensing fees to support

For peer review only - http://bmjopen.bmj.com/site/about/guidelines.xhtml

their businesses, a fact that should be considered if longer prescription lengths are to be adopted in practice. The magnitude of the savings for the other case studies will be of a similar scale given the prevalence of the conditions and frequency of shorter prescriptions. These figures should be interpreted with caution as they assume it is clinically appropriate for all prescriptions to be issued for a longer duration, which will certainly not be the case.

Comparison to previous studies

Several other studies have examined the costs associated with issuing either long (three-month) or short (one-month) supplies of prescriptions.[2-7] These studies all take the perspective of various payers in the US (e.g., different state-level Veterans Affairs and Medicaid programs as well as a non-institutionalised civilian population) and account for different cost items. Two studies found savings associated with longer prescriptions of a similar magnitude to ours, for example TUC of US$2.45 (£1.63 at April 2015 exchange rates)[5] and US$6.17 (£4.10)[3]. The former study[5] excluded prescriber time (the equivalent figure in our study is £1.03), and the latter[3] included costs of mail-order prescriptions.

Another study calculated per patient per year savings of US$7.70 (statins) to US$26.86 (oral hypoglycaemics) associated with 90- vs 30-day prescriptions.[6] A study of the financial impact on health care payers[4] detected statistically significant savings with 3-month supplies in only two of six cases as most savings accrued to patients through reductions in out-of-pocket costs. This study did not consider the cost of medication wastage making comparison with our study difficult. A simulation study found that any savings from reduced wastage from a shorter prescription length were more than offset by increases in dispensing fees as long as the dispensing fee was at least US$2.40 (base case assumption was US$5.60).[2]

In contrast to these, a comprehensive study on the impact of a policy to reduce the maximum prescription length from 100 to 34 days' supply in the North Carolina Medicaid

For peer review only - http://bmjopen.bmj.com/site/about/guidelines.xhtml

program[7] found that total Medicaid expenditures (comprising outpatient, inpatient, emergency as well as pharmacy costs) decreased for patients initially receiving 100 day prescriptions after the implementation of the 34-day policy (range US$245 to $440 per-person per-quarter across six classes of medications [anti-hypertensives, anti-diabetic medications, lipid-lowering drugs, seizure-disorder medications, antidepressants and antipsychotics] assessed). However, the results are not broken down by expenditure category (except for reporting decreases in expenditures for the targeted prescriptions across all six medication classes) and therefore it is unclear where the savings are accrued. This finding may be explained by small adverse health effects as a result of changes in adherence, patients absorbing any health effects through informal care or tolerating greater disease burden, or the follow-up period of the study (18 months post implementation) being too short to capture any spill-over effects of decreased adherence on other Medicaid services. The equivalent impact on NHS expenditure in the UK may differ due to differences in the organisation of care, in particular the gate-keeper role of primary care. Analysis of this was outside the scope of our analysis but would be a valuable future line of enquiry.

Study limitations

To the best of the authors' knowledge, this study provides the only evidence of the unnecessary costs associated with different prescription lengths from the perspective of the NHS in the UK and builds on existing methodological approaches available in the literature. However, there are a few limitations that warrant discussion. First, CPRD prescription data only indicates whether a prescription has been issued and not whether it was dispensed or taken as recommended. Our estimates may, therefore, either over- or understate the amount of wastage that actually occurred, depending on patient behaviour not captured in CPRD.

Second, the five case study conditions were purposively rather than randomly selected to represent the impact of repeat prescriptions and switching behaviour on wastage; they may

For peer review only - http://bmjopen.bmj.com/site/about/guidelines.xhtml

For peer review only

not be representative of prescribing behaviour in other chronic conditions. However, those selected do represent some of the most common chronic conditions treated with prescribed medications. Nine of the top 20 prescribed medications within NHS England were included in at least one of the case study conditions in our analyses and combined they accounted for around £378 million (4%) of all drug expenditure within NHS England in 2015 and are therefore highly policy relevant.[17] Our analysis also excluded patients having one or more observations with missing or zero values for either the ndd and/or qty variables. If this was non-random then the subsequent samples may not be truly representative of the general population. Appropriate methods to impute these variables are of limited value and our approach was similar to other studies using CPRD data.[9 10]

Third, the identification of patients within CPRD for the five case studies (Table 1) were based solely on product codes, rather than in conjunction with medical diagnoses. It is therefore possible that some of the patients in the five cohorts may be receiving medications for other conditions not of specific interest in our study (e.g., anti-depressants used for anxiety or metformin used for polycystic ovary disease). However, as the main aim of our study was to estimate drug wastage, the possible inclusion of patients with conditions outside our cohort definitions still provided our analysis with relevant information concerning drug wastage, dispensing fees and prescriber time.

Fourth, an overlap of dates between prescriptions does not necessarily mean wastage has occurred as consumption of early repeat prescriptions may be delayed until the initial supply is exhausted and treatment changes might actually be add-ons to existing prescriptions or concomitant therapy rather than switches in therapy. To ensure wastage was not overestimated a threshold of one year after the initial prescription in a particular series was used to estimate wastage for early repeat prescriptions and a threshold of <1 in the difference between the number of drug changes between medications with similar clinical indications

For peer review only - http://bmjopen.bmj.com/site/about/guidelines.xhtml

from different classes and the number of unique drug classes within an annual period was used to identify wastage from between class treatment switches. There is, however, the possibility that our analysis approach could be overestimating the amount of medication wastage.

Fifth, for pragmatic purposes we dichotomised prescription lengths into 'short' versus 'long', with a cut-off of 60 days. This will have classified 56 day prescriptions as 'short'. Whilst this will have resulted in a loss of sensitivity (there may be differences in TUC between one and two month prescriptions), the overall conclusions comparing 'shorter' (<60 day) and 'longer' (≥60 day) lengths are not affected.

Finally, a number of assumptions were required to assign unit costs to the estimated proportions of wastage. Mean cost per day values derived using DDDs, NICs and quantities at the drug substance level were calculated and then applied to any prescription categorised under that particular drug substance. This approach is not ideal, but necessary given the inability to link CPRD data to individual unit costs specific for each prescription. The direction and magnitude of any resulting bias is difficult to predict.

Furthermore, NICs do not include any discounts that may be applied or include any adjustment for revenue received by the NHS where a prescription charge is paid at the time the prescription is dispensed or where the patient has purchased a pre-payment certificate, and therefore maybe different from the net cost incurred specifically by the NHS. Patients with T2DM are exempt from the prescription charge,[18] and overall almost 90% of prescriptions dispensed in the NHS in England are exempt.[19]

All these limitations risk biasing the results. The projected savings should therefore be interpreted with caution and in any case be considered upper limits. Our analysis focused on

For peer review only - http://bmjopen.bmj.com/site/about/guidelines.xhtml

drugs with low unit costs prescribed to large numbers of patients. The results may not be generalizable to high cost drugs used to treat relatively small patient groups.

CONCLUSIONS

Overall, the findings from the study indicate that from the perspective of the NHS in the UK, longer prescription lengths are cost-saving relative to shorter prescription lengths in a number of common chronic diseases. Policy-makers should recommend that GPs consider issuing longer prescriptions for common chronic conditions where clinically appropriate to minimise the costs associated with dispensing fees and prescriber time as a result of issuing multiple prescriptions of shorter length.

For peer review only

For peer review only - http://bmjopen.bmj.com/site/about/guidelines.xhtml

CONTRIBUTORS

BD designed the study protocol, extracted, analysed and interpreted the data; drafted and revised this article; and gave final approval of this version to be published. AH extracted and assisted in organising the data, reviewed and edited the draft article and gave final approval of this version to be published. RP and EW conceptualised the study, assisted with its design and the interpretation of data, critically reviewed and edited the draft article and gave final approval of this version to be published.

COMPETING INTERESTS

All authors have no relevant affiliations or financial involvement with any organization or entity with a financial interest in or financial conflict with the subject matter or materials discussed in the manuscript, except support from a government grant as detailed in the acknowledgements. This includes employment, consultancies, grants, honoraria, stock ownership or options, expert testimony, or patents received or pending royalties. No writing assistance was utilised in the production of this manuscript.

DATA SHARING STATEMENT

No additional data available.

For peer review only

REFERENCES

1. Organization for Economic Cooperation and Development (OECD). Pharmaceutical expenditure. Health at a Glance 2011: OECD Indicators: OECD Publishing, 2011.

2. Domino ME, Olinick J, Sleath B, et al. Restricting patients' medication supply to one month: saving or wasting money? Am J Health Syst Pharm 2004;**61**(13):1375-9.

3. Parikh SP, Dishman BR, Smith TL. Ninety-day versus thirty-day drug-dispensing systems. Am J Health Syst Pharm 2001;**58**(13):1190-1.

4. Rabbani A, Alexander GC. Cost savings associated with filling a 3-month supply of prescription medicines. Appl Health Econ Health Policy 2009;**7**(4):255-64.

5. Walton SM, Arondekar BV, Johnson NE, et al. A model for Comparing Unecessary Costs Associated with Various Prescription Fill-Quantity Policies: illustration Using VA Data. J Managed Care Pharm 2001;**7**(5):384-90.

6. Taitel M, Fensterheim L, Kirkham H, et al. Medication Days' Supply, Adherence, Wastage, and Cost Among Chronic Patients in Medicaid. Medicare & Medicaid Research Review 2012;**2**(3):E1-E13.

7. Domino ME, Martin BC, Wiley-Exley E, et al. Increasing time costs and copayments for prescription drugs: an analysis of policy changes in a complex environment. Health Serv Res 2011;**46**(3):900-19.

8. Herrett E, Gallagher AM, Bhaskaran K, et al. Data Resource Profile: Clinical Practice Research Datalink (CPRD). Int J Epidemiol 2015;**44**(3):827-36.

9. Brodie MJ, Chung S, Wade A, et al. Clobazam and clonazepam use in epilepsy: Results from a UK database incident user cohort study. Epilepsy Res 2016;**123**:68-74.

10. Francis NA, Hood K, Lyons R, et al. Understanding flucloxacillin prescribing trends and treatment non-response in UK primary care: a Clinical Practice Research Datalink (CPRD) study. J Antimicrob Chemother 2016;**71**(7):2037-46.

11. WHO Collaborating Centre for Drug Statistics Methodology. ATC/DDD Index 2016. 16/12/2015 ed. Oslo, Norway: WHO, 2016.

12. National Health Service (NHS) - Prescription Services. Prescription Cost Analysis (PCA) Data. London, UK: NHS, December 2015.

13. National Health Service (NHS) - Prescription Services. Electronic Drug Tariff. Surrey, UK: NHS, December 2015.

14. Hollingworth W, Devine EB, Hansen RN, et al. The impact of e-prescribing on prescriber and staff time in ambulatory care clinics: a time motion study. J Am Med Inform Assoc 2007;**14**(6):722-30.

15. Curtis L, Burns A. *Unit Costs of Health and Social Care 2015*. Canterbury, UK: Personal Social Services Research Unit, The University of Kent, 2015.

16. Coupland C, Dhiman P, Morriss R, et al. Antidepressant use and risk of adverse outcomes in older people: population based cohort study. BMJ 2011;**343**:d4551.

For peer review only - http://bmjopen.bmj.com/site/about/guidelines.xhtml

17. National Health Service (NHS) - Business Services Authority. Prescription Cost Analysis (PCA) Data, 2015.

18. National Health Service (NHS) Choices. NHS in England - help with health costs. Secondary NHS in England - help with health costs 2016. http://www.nhs.uk/NHSEngland/Healthcosts/Pages/Prescriptioncosts.aspx.

19. Health and Social Care Information Centre. *Prescriptions dispensed in the community; England 2005-15*. Leeds: Health and Social Care Information Centre, 2016.

20. National Institute for Health and Care Excellence (NICE). British National Formulary (BNF). London, UK: NICE, 2016.

For peer review only

Table 1. Case study conditions and associated prescriptions

**Case study**	**Relevant prescriptions/patient inclusion criteria**
glucose control with oral drug therapy in type II diabetes mellitus	patients receiving one or more prescriptions for an oral anti-diabetic drug listed under the BNF Section 6.1.2 Antidiabetic drugs in any year from 2004 to 2014
treatment of hypertension in type II diabetes mellitus	in addition to receiving an oral anti-diabetic drug as defined in (1), patients receiving one or more prescriptions for any angiotensin-converting enzyme inhibitors, angiotensin II receptor antagonists, calcium-channel blockers, beta-adrenoceptor blockers, alpha-adrenoceptor blockers, potassium-sparing diuretics and/or thiazide-like diuretics in any year from 2004 to 2014
treatment with statins (lipid management) in type II diabetes mellitus	in addition to receiving an oral anti-diabetic drug as defined in (1), patients receiving one or more prescriptions for a statin in any year from 2004 to 2014
treatment for the secondary prevention of myocardial infraction	in addition to receiving concurrent[a] prescriptions for an angiotensin-converting enzyme inhibitor, antiplatelet and statin for at least one year in duration, patients receiving one or more prescriptions for beta-adrenoceptor blockers and/or angiotensin II receptor antagonists in any year from 2004 to 2014
treatment of depression	patients receiving one or more prescriptions for any anti-depressant drug listed under BNF Section 4.3 Antidepressant drugs in any year from 2004 to 2014

BNF – British National Formulary

[a]All patients receiving at least one prescription for an angiotensin-converting enzyme inhibitor, antiplatelet drug and statin were first identified in CPRD. Patients from this sample that did not have at least four prescriptions (chosen to represent one year of therapy) for each of these drugs in at least one of the 11 years of data available (i.e., 2004 to 2014) were excluded. From the remaining patients the additional constraint of receiving one or more prescriptions for any beta-adrenoceptor blockers and/or angiotensin II receptor antagonists was applied to define the full sample.

For peer review only

Table 2. Comparison of the mean cost of medication wastage per prescription over ten-year period 2004-2014 by treatment pattern (2015 £)

	Mean cost of repeat prescription wastage per prescription 2015 £ (95% CI)		Mean cost of dosage/ formulation switch wastage per prescription		Mean cost of within class treatment switch wastage per prescription 2015 £ (95% CI)		Mean cost of treatment between class switch wastage per prescription 2015 £ (95% CI)	
	<60 days	≥60 days	<60 days	≥60 days	<60 days	≥60 days	<60 days	≥60 days
Glucose control with oral drug therapy in T2DM	0.230 (0.226-0.233)	1.035 (0.772-1.298)	0.059 (0.058-0.061)	0.173 (0.143-0.204)	0.031 (0.029-0.032)	0.097 (0.080-0.114)	0.009 (0.008-0.010)	0.064 (0.051-0.078)
Hypertension in T2DM	0.050 (0.049-0.051)	0.271 (0.228-0.314)	0.038 (0.038-0.039)	0.128 (0.107-0.149)	0.004 (0.003-0.004)	0.013 (0.009-0.016)	0.003 (0.003-0.003)	0.026 (0.022-0.030)
Lipid management in T2DM	0.017 (0.017-0.017)	1.099 (0.832-1.367)	0.024 (0.023-0.024)	0.153 (0.081-0.225)	0.008 (0.007-0.008)	0.173 (0.075-0.271)	NA	
Secondary prevention of myocardial infraction	0.043 (0.042-0.043)	0.439 (0.300-0.578)	0.014 (0.014-0.014)	0.040 (0.036-0.045)	0.009 (0.009-0.009)	0.029 (0.027-0.031)	0.00005 (0.00004-0.00006)	0.0006 (0.0003-0.0008)
Depression	0.044 (0.042-0.046)	0.214 (0.180-0.249)	0.146 (0.143-0.150)	0.141 (0.113-0.169)	0.006 (0.005-0.007)	0.013 (0.004-0.021)	0.012 (0.010-0.013)	0.061 (0.036-0.086)

CI – confidence interval; NA – not applicable; T2DM – type II diabetes mellitus

For peer review only

For peer review only - http://bmjopen.bmj.com/site/about/guidelines.xhtml

Box 1. Example of differentiating between treatment switches and add-ons for a patient receiving medications for hypertension

In 2011, the patient has one change between clinically related drugs from different classes (ramipril to losartan) and receives medication belonging to two unique drug classes (ACE and ARA). One minus two is <1, so this change is considered a switch. The rationale being, if the number of changes was small or large and the number of unique drugs involved in the changes were also small or large respectively, switches in therapies were occurring and therefore there was potential for wastage to occur. In 2012, the patient has two changes between clinically related drugs from different classes (losartan to diltiazem and diltiazem to losartan) and receives medication belonging to two unique drug classes (ARA and CCB). Two minus two is <1, which indicates a switch, but in the treatment of hypertension ARAs and CCBs are commonly administered together as second-line therapy[20] and therefore these two changes were considered add-ons/concomitant therapy. In 2013, the patient has four changes between clinically related drugs from different classes (losartan to doxazosin, doxazosin to losartan, losartan to doxazosin and doxazosin to losartan) and receives medication belonging to two unique drug classes (ARA and AAB). Four minus two is ≥1, which indicates the four changes are add-ons, not switches. The rationale being that if the number of changes was large, but the number of unique drugs involved in the changes was low, an add-on or concomitant therapy was being prescribed and no wastage was occurring.

Year	Sequence of Prescriptions in Year	Drug	Class	Total Number of Treatment Switches Between Classes in Year (A)	Total Number Unique Classes in Year (B)	Difference for Year (A) - (B)	Count as Treatment Switch Between Classes[a]	Count as Add On
2011	1	Ramipril	ACE	1	2	-1	No	No
2011	2	Losartan potassium	ARA				Yes	No
2011	3	Losartan potassium	ARA				No	No
2011	4	Losartan potassium	ARA				No	No
2012	1	Losartan potassium	ARA	2	2	0	No	No
2012	2	Diltiazem hydrochloride	CCB				No	Yes
2012	3	Diltiazem hydrochloride	CCB				No	No
2012	4	Losartan potassium	ARA				No	Yes
2013	1	Losartan potassium	ARA	4	2	2	No	No
2013	2	Doxazosin	AAB				No	Yes
2013	3	Losartan potassium	ARA				No	Yes
2013	4	Doxazosin	AAB				No	Yes
2013	5	Losartan potassium	ARA				No	Yes

AAB – alpha-adrenoceptor blocker; ACE - angiotensin-converting enzyme inhibitor; ARA – angiotensin-II receptor antagonist; CCB – calcium channel blocker

[a]For the treatment of hypertension in T2DM cohort, overlaps in prescription dates involving angiotensin-converting enzyme inhibitors and angiotensin-II receptor antagonists with either calcium-channel blockers or thiazide-like diuretics were not counted as switches as these therapies are commonly administered together as second-line therapy.[20]

For peer review only

For peer review only - http://bmjopen.bmj.com/site/about/guidelines.xhtml

Box 2. Example comparing total unnecessary costs for <60 day and ≥60 day prescription lengths for a standardised time period of 120 days

Assume on average that the <60 day prescription length is 35 days and the average ≥60 day prescription lengths is 120 days. Also assume that regardless of prescription length, patients on average switch their prescription 30 days after a prescription is issued. The quantity used is therefore 30 days for both prescription lengths ($Q_{daysused<60} = Q_{daysused\geq60} = 30$), but the quantity wasted is much larger for the ≥60 day prescription (90 days compared to only five days wasted). Since over a 120-day period both prescription lengths will incur the same dispensing fees and prescriber time costs (four prescriptions will be issued regardless of prescription length as a switch occurs every 30 days) the ≥60 day prescription will be associated with higher total unnecessary costs. Note this example has been developed by adapting an example provided by Walton et al.[5] to the prescription lengths considered in our study.

For peer review only

For peer review only - http://bmjopen.bmj.com/site/about/guidelines.xhtml

FIGURE LENGENDS

Figure 1. Trends in the mean cost of medication wastage per prescription over 11-year study period

For peer review only

For peer review only - http://bmjopen.bmj.com/site/about/guidelines.xhtml

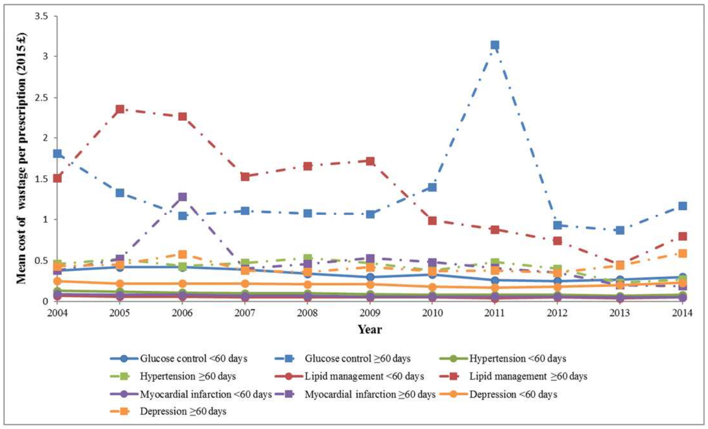

Figure 1. Trends in the mean cost of medication wastage per prescription over 11-year study period

190x107mm (300 x 300 DPI)

review only

Appendix I – CPRD product code lists of the potentially prescribed medications for each of the five case study conditions

Appendix I is available from the authors upon request as an Excel file containing five worksheets (one for each of the five case study conditions). Within each worksheet the lists of product codes obtained from the CPRD Research Applications Code Browser Version 3.0.0.0 that represent the possible medications that patients may be prescribed for the treatment of one of the five case study conditions is presented. The table below provides an example of the codes listed available in the associated Excel file. Please note that the lists in the table below are not comprehensive and readers should request the associated Excel file for the complete product code lists.

CPRD product code list

Study condition	Product code
Glucose control with oral drug therapy in type II diabetes mellitus	2219
	7912
	16602
	26218
	41558
Treatment of hypertension in type II diabetes mellitus	2
	58
	1209
	1211
	1213
Lipid management in type II diabetes mellitus	65193
	63140
	55034
	51200
	7374
Secondary prevention of myocardial infraction	59699
	56850
	34544
	3310
	26995
Treatment of depression	2525
	48065
	4690
	8831

For peer review only

Appendix II - Data processing of the five cohorts

Condition	Full sample (Patients)	Full sample (Rx)	ndd=0 or qty=0 (Patients)	ndd=0 or qty=0[a] (Rx)	Limited sample[b] (Patients)	Limited sample[b] (Rx)	Random sample (Patients)	Random sample (Rx)	Dropped prescription error[c] (Rx)	Final sample (Rx)	Same day switches not wastage[d] (Rx)
Glucose control with oral drug therapy in T2DM	310,391	21,091,529	170,967	4,518,765	139,424	7,135,397	50,000	2,577,282	6,483	2,570,799	548,850
Hypertension in T2DM	230,760	23,886,597	63,802	1,446,199	166,958	16,041,452	50,000	4,803,444	3,983	4,799,461	1,588,921
Lipid management in T2DM	242,741	13,388,759	36,577	776,718	206,164	11,216,086	50,000	2,718,216	913	2,717,303	NA
Secondary prevention of myocardial infraction	208,682	44,151,527	87,281	3,270,504	121,401	24,479,014	50,000	10,131,377	767	10,130,610	5,856,361
Depression	1,207,523	32,744,994	424,446	4,438,319	783,077	15,712,941	50,000	1,010,463	3,234	1,007,229	12,401

ndd – numeric daily dose; NA – not applicable; qty – quantity; Rx – prescriptions; T2DM – type II diabetes mellitus

[a]The product codes that most frequently had qty or ndd values equal to zero were: metformin 500 mg tablets (38%) and gliclazide 80 mg tablets (26%) for the T2DM cohort; ramipril 10 mg capsules (9%), bendroflumethiazide 2.5 mg tablets, Ramipril 5 mg capsules, Ramipril 2.5 mg capsules and amlodipine 5 mg tablets for the hypertension in T2DM cohort; simvastatin 40 mg tablets (38%), simvastatin 20 mg tablets (16%), atorvastatin 40 mg tablets (10%) and atorvastatin 20 mg tablets (10%) for the lipid management in T2DM cohort; aspirin 75 mg dispersible tablets (21%) and simvastatin 40 mg tablets (12%) for the secondary prevention of myocardial infarction cohort; and citalopram 20 mg tablets (13%) and fluoxetine 20 mg capsules (11%) for the depression cohort.

[b]The limited sample consists of all patients that have received at least one of the relevant prescriptions for a respective case study condition and do not have any missing or observations equal to zero for both the ndd and qty variables.

[c]Prescriptions issued on the same day for medications in the same class and with different product codes (e.g., two different selective serotonin reuptake inhibitors or two different statins) were considered prescriber error and dropped from the analysis.

[d]Prescriptions for medications with similar clinical indications from different classes issued on the same day were assumed not to incur wastage due to a treatment switch, but rather were assumed to be an add-on to therapy or concomitant therapy.

For peer review only

Appendix III – Description of methods used to estimate medication wastage

Wastage was defined as either a repeat prescription or new prescription based on the three types of substitutions/switches (substitutions between different dosages or formulations of the same drug substance; substitutions between drugs that are in the same class; and substitutions between drugs that have similar clinical indications from different classes) respectively, being issued prior to the expiry of the previously prescribed quantity. The volume of medication wastage from early repeat prescriptions and treatment switches was estimated for prescriptions within the 11-year study period (i.e., any prescription not falling in this time period were excluded from the analyses). This was done by dividing the total quantity entered by the GP for the prescribed product (qty) by the numeric daily dose prescribed for the event (ndd) and comparing this to the difference in the two dates associated with the event and the next prescription in the sequence. Prescriptions issued on the same day for drugs in the same class with different product codes (e.g., two different statins) were considered prescriber error and dropped from the analysis. The one exception to this was for antiplatelet drugs in the secondary prevention of myocardial infarction cohort, as it was assumed that two different antiplatelet drugs could be prescribed at the same time. In addition, prescriptions for medications with similar clinical indications from different classes issued on the same day were not counted as a switch, but rather as an add-on to existing therapy or concomitant therapy.

As treatment patterns were assessed in sequence there was the potential to overstate the amount of wastage that occurred. For example, counting every overlap of prescription days associated with repeat prescriptions of the same product as wastage (i.e., even prescriptions issued one day before the expiry of the previous prescription would be counted as one day's worth of medication wastage or two prescriptions issued on the same day would count one prescription as entirely wasted) may overstate actual wastage. A threshold of one

For peer review only

year after the initial prescription in a particular series was, therefore, used to estimate wastage for early repeat prescriptions, in that any product prescribed over and above the expected quantity to be consumed within the one-year time period was considered waste. This is to account for the fact that patients may fill their prescriptions before their existing supply is exhausted, but still consume all of the previous prescription before using the new supply.

In addition, while excessive switching of drugs could appear as wastage, consistent patterns could suggest a valid, prescribed treatment regimen. To avoid the overestimation of wastage, additional effort was made to differentiate between add-ons/concomitant therapy and switches for medications with similar clinical indications from different classes (i.e., product, drug substance and drug class codes are different for two prescriptions in sequence). Generally, if the difference between the number of changes between medications with similar clinical indications from different classes and the number of unique drug classes within an annual period was ≥1, then any overlap in prescription dates was not considered wastage due to a switch, but rather as an add-on or concomitant therapy. For example, within an annual period a patient may have six changes between clinically related drugs from different classes, but these changes are only between two different drugs from different classes (i.e., two unique drug classes). Since six minus two is ≥1 these changes were not considered switches, but rather add-on/concomitant prescriptions. The rationale being that if the number of changes was large, but the number of unique drugs involved in the changes was low an add-on or concomitant therapy was being prescribed, whereas if the number of changes was large and the number of unique drugs involved in the changes was also large, switches in therapies were occurring and therefore there was the potential for wastage to occur. A similar approach was applied by Taitel et al.[1] and is the only previous study that has attempted to differentiate between add-on/concomitant prescriptions and actual switches in therapy. Additional constraints in counting overlaps in dates for two prescriptions in sequence for medications

For peer review only

with similar clinical indications from different classes were also applied in three of the case study conditions due to the potential for a number of the included therapies to be given concomitantly. For the glucose control in T2DM cohort overlaps in prescription dates involving metformin with drug from other classes were not counted as switches (and therefore wastage) as metformin is usually administered in combination with other classes of oral anti-diabetics.[2] For the treatment of hypertension in T2DM cohort, overlaps in prescription dates involving angiotensin-converting enzyme inhibitors and angiotensin-II receptor antagonists with either calcium-channel blockers or thiazide-like diuretics were not counted as switches as these therapies are commonly administered together as second-line therapy.[3] Finally, for the secondary prevention of myocardial infraction cohort, only overlapping prescriptions dates involving beta-blockers and angiotensin-II receptor antagonists were counted as switches as patients are likely to receive the other classes of drugs included in the analysis (angiotensin-converting enzyme inhibitors, antiplatelet drugs and statins) continuously over the course of treatment.[4]

For peer review only

Appendix IV – Unit prescription drug cost calculations

Drug substance	DDD (mg)	NIC from PCA (pence)	Quantity from PCA	Strength from PCA (mg)	NIC/ Quantity (£)	Cost per day[a] (£)
**Initial glucose control in type II diabetes**						
glibenclamide	7	1,072,539	279,251	5	0.04	0.05
gliclazide	60	3,322,771	195,041	60	0.17	0.17
glimepiride	2	2,650,357	685,422	2	0.04	0.04
glipizide	10	8,679,376	762,384	5	0.11	0.23
tolbutamide	1500	17,494,096	266,373	500	0.66	1.97
metformin	2000	531,088,447	111,025,385	500	0.05	0.19
acarbose	300	108,607	1,234	100	0.88	2.64
alogliptin	25	6,918,604	10,147	25	6.82	6.82
canagliflozin	200	58,466,442	447,445	100	1.31	2.61
dapagliflozin	10	191,937,124	1,468,764	10	1.31	1.31
empagliflozin	17.5	16,291,281	124,666	10	1.31	2.29
exenatide	1	10,031,280	1,470	60	68.24	1.14
linagliptin	5	271,703,001	2,287,246	5	1.19	1.19
liraglutide	1.2	337,718,696	86,033	6	39.25	7.85
lixisenatide	0.02	45,851,757	15,830	0.28	28.97	2.07
nateglinide	360	631,585	17,827	180	0.35	0.71
pioglitazone	30	144,198,158	1,125,016	30	1.28	1.28
repaglinide	4	1,620,508	246,350	2	0.07	0.13
saxagliptin	5	80,113,342	709,874	5	1.13	1.13
sitagliptin	100	670,533,026	5,644,766	100	1.19	1.19
vildagliptin	100	18,582,165	312,012	50	0.60	1.19
rosiglitazone[b]	6	-	-	-	-	2.34
guar gum[b,d]	68.5	-	-	-	-	2.34
dulaglutide	0.16	1,717,714	938	0.75	18.31	3.91
**Hypertension in type II diabetes**						
bendroflumethiazide	2.5	115,395,532	38,916,033	2.5	0.03	0.03
chlortalidone	25	410	70	50	0.06	0.03
cyclopenthiazide[b]	0.5	-	-	-	-	1.07
indapamide	2.5	40,603,410	7,849,048	2.5	0.05	0.05
xipamide	20	274,581	19,761	20	0.14	0.14
chlorothiazide	500	92,160	200	500	4.61	4.61
hydrochlorothiazide	25	743,206	4,701	25	1.58	1.58
Hydroflumethiazide[b]	25	-	-	-	-	1.07
candesartan cilexetil	8	27,228,854	6,187,293	8	0.04	0.04
eprosartan	600	7,848,330	156,629	600	0.50	0.50
irbesartan	150	15,321,402	2,527,161	150	0.06	0.06
losartan potassium	50	43,142,921	10,854,773	50	0.04	0.04
olmesartan medoxomil	20	23,454,180	507,120	20	0.46	0.46
telmisartan	40	2,105,968	429,294	40	0.05	0.05
valsartan	80	7,680,126	790,946	80	0.10	0.10

For peer review only

Drug substance	DDD (mg)	NIC from PCA (pence)	Quantity from PCA	Strength from PCA (mg)	NIC/ Quantity (£)	Cost per day[a] (£)
captopril	50	1,209,326	274,077	50	0.04	0.04
enalapril maleate	10	8,273,970	2,164,274	10	0.04	0.04
fosinopril sodium	15	1,717,978	32,658	10	0.53	0.79
imidapril hydrochloride	10	523,685	20,308	10	0.26	0.26
lisinopril	10	30,787,577	8,978,102	10	0.03	0.03
moexipril hydrochloride	15	22,272	896	15	0.25	0.25
perindopril erbumine	4	25,785,508	604,0253	4	0.04	0.04
perindopril arginine	4	1,125,376	53,760	5	0.21	0.17
quinapril	15	2,130,784	69,778	10	0.31	0.46
ramipril	2.5	75,859,752	18,621,897	2.5	0.04	0.04
ramipril with felodipine	2.5	456,727	5,209	2.5	0.88	0.88
trandolapril	2	4,668,257	191,955	2	0.24	0.24
cilazapril	2.5	1,662,225	8,985	5	1.85	0.93
perindopril tosilate	4	11,821	594	5	0.20	0.16
amlodipine	5	153,321,726	47,137,956	5	0.03	0.03
diltiazem hydrochloride	240	3,855,905	93,720	240	0.41	0.41
felodipine	5	63,115,662	4,197,918	5	0.15	0.15
isradipine	5	4,429,879	13,442	2.5	3.30	6.59
lacidipine	4	14,646,423	910,750	4	0.16	0.16
lercanidipine hydrochloride	10	77,973,540	3,825,663	10	0.20	0.20
nicardipine hydrochloride	90	397,902	35,119	30	0.11	0.34
nifedipine	30	3,656,530	149,471	30	0.24	0.24
nisoldipine[b]	20	-	-	-	-	0.93
verapamil hydrochloride	240	7,104,750	358,394	240	0.20	0.20
doxazosin	4	52,769,814	14,187,844	4	0.04	0.04
indoramin[d]	4.7	5,674,414	34,4947	20	0.16	0.04
prazosin	5	74,527	205	5	3.64	3.64
terazosin	5	1,372,116	136,216	5	0.10	0.10
amiloride hydrochloride	10	18,835,995	565,354	5	0.33	0.67
amiloride hydrochloride with thiazide	10	868,138	75,026	5	0.12	0.23
triamterene	100	211,025	1,511	50	1.40	2.79
triamterene with thiazide	100	160,534	4,540	50	0.35	0.71
spironolactone	75	24,174,720	4,339,112	25	0.06	0.17
atenolol	75	25,567,900	8,522,336	25	0.03	0.09
**Hyperlipidaemia in type II diabetes**						
atorvastatin	20	158,989,904	31,324,266	20	0.05	0.05
fluvastatin	60	1,475,946	163,264	20	0.09	0.27

Drug substance	DDD (mg)	NIC from PCA (pence)	Quantity from PCA	Strength from PCA (mg)	NIC/ Quantity (£)	Cost per day[a] (£)
pravastatin	30	6,780,883	1,648,896	10	0.04	0.12
rosuvastatin	10	134,267,696	2,085,108	10	0.64	0.64
simvastatin	30	15,635,680	5,206,048	10	0.03	0.09
**Secondary prevention of myocardial infraction**						
captopril	50	1,209,326	274,077	50	0.04	0.04
enalapril maleate	10	8,273,970	2,164,274	10	0.04	0.04
fosinopril sodium	15	1,717,978	32,658	10	0.53	0.79
lisinopril	10	30,787,577	8,978,102	10	0.03	0.03
perindopril erbumine	4	25,785,508	6,040,253	4	0.04	0.04
perindopril arginine	4	1,125,376	53,760	5	0.21	0.17
quinapril	15	728,515	23,878	5	0.31	0.92
ramipril	2.5	75,859,752	18,621,897	2.5	0.04	0.04
ramipril with felodipine	2.5	456,727	5,209	2.5	0.88	0.88
trandolapril	2	4,668,257	191,955	2	0.24	0.24
cilazapril	2.5	1,662,225	8,985	5	1.85	0.93
perindopril tosilate	4	11,821	594	5	0.20	0.16
acebutolol	400	1,202,284	18,079	400	0.67	0.67
atenolol	75	25,567,900	8,522,336	25	0.03	0.09
bisoprolol	10	20,420,456	5,828,018	10	0.04	0.04
carvedilol	37.5	2,684,299	577,569	6.25	0.05	0.28
metoprolol tartrate	150	15,398,869	2,318,257	50	0.07	0.20
aspirin[d]	127.5	154,533,218	52,719,924	75	0.03	0.05
clopidogrel	75	129,484,315	19,793,087	75	0.07	0.07
ticagrelor	180	190,143,810	1,950,196	90	0.97	1.95
atorvastatin	20	158,989,904	31,324,266	20	0.05	0.05
fluvastatin	60	1,475,946	163,264	20	0.09	0.27
pravastatin	30	6,780,883	1,648,896	10	0.04	0.12
rosuvastatin	10	134,267,696	2,085,108	10	0.64	0.64
simvastatin	30	15,635,680	5,206,048	10	0.03	0.09
candesartan cilexetil	8	27,228,854	6,187,293	8	0.04	0.04
eprosartan	600	7,848,330	156,629	600	0.50	0.50
irbesartan	150	15,321,402	2,527,161	150	0.06	0.06
losartan potassium	50	43,142,921	10,854,773	50	0.04	0.04
olmesartan medoxomil	20	23,454,180	507,120	20	0.46	0.46
telmisartan	40	2,105,968	429,294	40	0.05	0.05
valsartan	80	7,680,126	790,946	80	0.10	0.10
**Depression**						
amitriptyline hydrochloride	75	42,327,326	12,089,330	25	0.04	0.11
amoxapine	150	65,772	168	100	3.92	5.87
clomipramine hydrochloride	100	5,343,700	663,607	50	0.08	0.16
dosulepin hydrochloride	150	9,835,231	1,582,426	75	0.06	0.12

For peer review only

Drug substance	DDD (mg)	NIC from PCA (pence)	Quantity from PCA	Strength from PCA (mg)	NIC/ Quantity (£)	Cost per day[a] (£)
doxepin[c]	100	-	-	50	0.20	0.41
imipramine hydrochloride	100	4,075,545	858,036	25	0.05	0.19
lofepramine	105	39,309,425	1,177,389	70	0.33	0.50
nortriptyline	75	115,762,972	1,077,134	25	1.07	3.22
trazodone hydrochloride	300	61,816,624	718,214	150	0.86	1.72
trimipramine	150	1,672	56	50	0.30	0.90
citalopram	20	86,323,713	23,901,861	20	0.04	0.04
escitalopram	10	5,540,518	1,121,092	10	0.05	0.05
fluoxetine	20	87,922,802	23,735,904	20	0.04	0.04
fluvoxamine maleate	100	4,073,827	55,899	100	0.73	0.73
paroxetine	20	25,034,806	3,116,825	20	0.08	0.08
sertraline	50	100,699,567	15,289,272	50	0.07	0.07
isocarboxazid	15	3,453,630	12,118	10	2.85	4.28
moclobemide	300	1,057,512	22,678	300	0.47	0.47
phenelzine	60	2,647,884	117,682	15	0.23	0.90
tranylcypromine	10	45,505,295	51,406	10	8.85	8.85
agomelatine	25	8,035,533	74,999	25	1.07	1.07
duloxetine	60	276,427,017	2,904,742	60	0.95	0.95
mirtazapine	30	26,199,391	4,793,099	30	0.05	0.05
reboxetine	8	4,467,383	141,747	4	0.32	0.63
tryptophan[d]	44.6	18,495	566	50	0.33	0.29
venlafaxine	100	17,440,533	3,256,789	75	0.05	0.07

DDD – defined daily dose; NIC – net ingredient cost; PCA – Prescription Cost Analysis

[a]Calculated as (NIC/Quantity) x (DDD/Strength from PCA).

[b]Data not available within the PCA for December 2015; cost per day based on an average of the values for other drugs within the same class.

[c]Data from PCA for December 2015 deemed unreliable; NIC/Quantity derived by dividing cost of 50 mg 28-cap pack by 28.

[d]Data for DDD not available; DDD based on an average of the values for other drugs within the same class.

For peer review only

Appendix V – Search strategy for prescriber time data

Date of Search: *July 8, 2016*

Databases: Ovid MEDLINE (R) Epub Ahead of Print, In-process & Other Non-Indexed Citations, Ovid MEDLINE (R) Daily and Ovid MEDLINE (R) 1946 to present; Embase 1974 to 2016 July 07

1	general practitioner.ab,hw,kf,kw,ot,ti,xs.
2	GP.ab,hw,kf,kw,ot,ti,xs.
3	physician.ab,hw,kf,kw,ot,ti,xs.
4	clinician.ab,hw,kf,kw,ot,ti,xs.
5	doctor.ab,hw,kf,kw,ot,ti,xs.
6	medic.ab,hw,kf,kw,ot,ti,xs.
7	consultant.ab,hw,kf,kw,ot,ti,xs.
8	medical specialist.ab,hw,kf,kw,ot,ti,xs.
9	physician assistant.ab,hw,kf,kw,ot,ti,xs.
10	physician associate.ab,hw,kf,kw,ot,ti,xs.
11	nurse.ab,hw,kf,kw,ot,ti,xs.
12	pharmacist.ab,hw,kf,kw,ot,ti,xs.
13	healthcare professional.ab,hw,kf,kw,ot,ti,xs.
14	medical professional.ab,hw,kf,kw,ot,ti,xs.
15	medical staff.ab,hw,kf,kw,ot,ti,xs.
16	1 or 2 or 3 or 4 or 5 or 6 or 7 or 8 or 9 or 10 or 11 or 12 or 13 or 14 or 15
17	prescriber time.ab,hw,kf,kw,ot,ti,xs.
18	staff time.ab,hw,kf,kw,ot,ti,xs.
19	time utilization.ab,hw,kf,kw,ot,ti,xs.
20	time utilisation.ab,hw,kf,kw,ot,ti,xs.
21	workload.ab,hw,kf,kw,ot,ti,xs.
22	workflow.ab,hw,kf,kw,ot,ti,xs.
23	work processes.ab,hw,kf,kw,ot,ti,xs.
24	medication management.ab,hw,kf,kw,ot,ti,xs.
25	17 or 18 or 19 or 20 or 21 or 22 or 23 or 24
26	time study.ab,hw,kf,kw,ot,ti,xs.
27	time motion study.ab,hw,kf,kw,ot,ti,xs.
28	time-motion study.ab,hw,kf,kw,ot,ti,xs.
29	(time and motion method).ab,hw,kf,kw,ot,ti,xs.
30	time-and-motion method.ab,hw,kf,kw,ot,ti,xs.
31	(time and motion study).ab,hw,kf,kw,ot,ti,xs.
32	time-and-motion study.ab,hw,kf,kw,ot,ti,xs.
33	time motion analysis.ab,hw,kf,kw,ot,ti,xs.
34	time-motion analysis.ab,hw,kf,kw,ot,ti,xs.
35	(before and after study).ab,hw,kf,kw,ot,ti,xs.
36	before-and-after study.ab,hw,kf,kw,ot,ti,xs.
37	26 or 27 or 28 or 29 or 30 or 31 or 32 or 33 or 34 or 35 or 36
**38**	16 and 25 and 37 – **Total Hits = 227**

For peer review only

Study selection details

The targeted literature search identified a total of 227 citations. After titles and abstracts were screened 216 citations were excluded and 11 citations were reviewed in full-text. Four studies contained relevant information and the most appropriate evidence was selected from the four studies by prioritising evidence from larger sample sizes and studies that reported prescriber time for different types of prescriptions (e.g., new versus renewals) and/or different types of prescribers (e.g., general practitioner versus nurse).[5-8] It should be noted that one of the four studies identified was a systematic review and led to the identification of two additional studies with relevant information based on their reporting of mean consultation times based on large sample sizes and in different types of prescribers.[9 10]

For peer review only

Appendix VI - Mean values used in the comparison of total unnecessary costs

	Glucose control with oral drug therapy in T2DM		Hypertension in T2DM		Lipid management in T2DM		Secondary prevention of myocardial infraction		Depression	
**Parameters**	**<60 days**	**≥60 days**	**<60 days**	**≥60 days**	**<60 days**	**≥60 days**	**<60 days**	**≥60 days**	**<60 days**	**≥60 days**
Mean days used ($Q_{daysused}$)	31.61	75.72	32.51	86.91	32.74	122.71	31.41	102.28	27.43	65.03
Mean days wasted ($Q_{dayswasted}$)	0.86	4.96	1.23	6.98	0.63	16.21	0.96	6.56	1.87	2.69
Mean drug cost per day (£) ($C_{drug/day}$)	0.36	0.28	0.084	0.082	0.10	0.10	0.074	0.068	0.14	0.11
Mean dispensing fee cost (£) ($C_{dispensing}$)	0.92	1.05	0.90	0.93	0.90	1.00	0.90	0.97	0.91	0.96
Mean prescriber (GP) time cost (£) ($C_{prescribertime}$)	3.39	3.44	3.54	3.55	3.12	3.15	3.76	3.77	3.23	3.18
Mean prescriber (Nurse) time cost (£) ($C_{prescribertime}$)	2.28	2.29	2.31	2.32	2.21	2.22	2.37	2.37	2.24	2.23

GP – general practitioner; T2DM – type II diabetes mellitus

For peer review only

Appendix VII – Study protocol (16_117R) approved on June 21, 2016 by the Independent Scientific Advisory Committee (ISAC)

ISAC APPLICATION FORM

PROTOCOLS FOR RESEARCH USING THE CLINICAL PRACTICE RESEARCH DATALINK (CPRD)

ISAC use only:		**IMPORTANT**
Protocol Number	....	**If you have any queries, please contact ISAC Secretariat:**
Date submitted	....	ISAC@cprd.com

Section A: The study

1. Study Title

Three months versus 28 day prescriptions: a retrospective analysis of CPRD data to determine differences in the cost of drug wastage, dispensing fees and prescriber time

2. Has any part of this research proposal or a related proposal been previously submitted to ISAC?

Yes ☐ No ☒

If Yes, please provide previous protocol numbers:

3. Has this protocol been peer reviewed by another Committee? (e.g. grant award or ethics committee)

Yes ☒ No ☐

If Yes, please state the name of the reviewing Committee(s) and provide an outline of the review process and outcome: Proposal peer reviewed as part of a successful application to NIHR Health Technology Assessment Programme.

4. Type of Study (please tick all the relevant boxes which apply)

Adverse Drug Reaction/Drug Safety ☐ Drug Utilisation ☒ Disease Epidemiology ☐
Drug Effectiveness ☐ Pharmacoeconomics ☒ Methodological ☐
Health/Public Health Services Research ☒ Post-authorisation Safety ☐
Other* ☐
*Please specify the type of study in the lay summary

5. This study is intended for (please tick all the relevant boxes which apply)**:**

Publication in peer reviewed journals ☒ Presentation at scientific conference ☒
Presentation at company/institutional meetings ☐ Regulatory purposes ☐
Other: Publication in NIHR HTA Monograph

Section B: The Investigators

6. Chief Investigator (full name, job title, organisation name & e-mail address for correspondence- see guidance notes for eligibility)

Name: Ed Wilson
Job title: Senior Research Associate in Health Economics
Organisation: Cambridge Centre for Health Services Research, University of Cambridge
Email: ew442@medschl.cam.ac.uk

CV has been previously submitted to ISAC ☐ **CV number:**
A new CV is being submitted with this protocol ☒
An updated CV is being submitted with this protocol ☐

7. Affiliation (full address)

Institute of Public Health, Cambridge CB2 0SR, UK

8. Corresponding Applicant

Name: Brett Doble
Job title: Research Associate in Health Economics
Organisation: Cambridge Centre for Health Services Research, University of Cambridge
Email: brett.doble@medschl.cam.ac.uk

Same as chief investigator ☐
CV has been previously submitted to ISAC ☐ **CV number:**
A new CV is being submitted with this protocol ☒
An updated CV is being submitted with this protocol ☐

9. List of all investigators/collaborators (*please list the full names, affiliations and e-mail addresses* of all collaborators, other than the Chief Investigator*)

Other investigator: Rupert Payne,

For peer review only - http://bmjopen.bmj.com/site/about/guidelines.xhtml

Job title: Consultant Senior Lecturer in Primary Health Care
Organisation: University of Bristol
Email: rupert.payne@bristol.ac.uk

CV has been previously submitted to ISAC ☒ **CV number:** 177_15CEP
A new CV is being submitted with this protocol ☐
An updated CV is being submitted with this protocol ☐

Other investigator: Sarah King
Job title: Visiting Fellow
Organisation: University of Cambridge
Email: sek23@cam.ac.uk
CV has been previously submitted to ISAC ☐ **CV number:**
A new CV is being submitted with this protocol ☒
An updated CV is being submitted with this protocol ☐

[Please add more investigators as necessary] *Please note that your ISAC application form and protocol **must** be copied to all e-mail addresses listed above at the time of submission of your application to the ISAC mailbox. Failure to do so will result in delays in the processing of your application.*

10. Conflict of interest statement* (please provide a draft of the conflict (or competing) of interest (COI) statement that you intend to include in any publication which might result from this work)
The authors do not have any conflict of interest.
**Please refer to the International Committee of Medical Journal Editors (ICMJE) for guidance on what constitutes a COI*

11. Experience/expertise available (please complete the following questions to indicate the experience/expertise available within the team of investigators/collaborators actively involved in the proposed research, including the analysis of data and interpretation of results

	Previous GPRD/CPRD Studies	Publications using GPRD/CPRD data
None	☐	☒
1-3	☐	☐
> 3	☒	☐

	Yes	No
**Is statistical expertise available within the research team?**<br>*If yes, please indicate the name(s) of the relevant investigator(s)*<br>Rupert Payne and Ed Wilson with additional support provided by the Cambridge CPRD User Group, which includes Senior Statistician Dr. Katie Saunders.	☒	☐
**Is experience of handling large data sets (>1 million records) available within the research team?**<br>*If yes, please indicate the name(s) of the relevant investigator(s)*<br>Rupert Payne, Brett Doble<br>• Rupert Payne previously managed Cambridge's institutional license for CPRD, and is familiar with the CPRD GOLD dataset, analysis of HES records, and has relevant experience from analysis of other large, linked primary-secondary care datasets. He also has extensive programming experience as well as database management skills.<br>• Brett Doble has had experience managing, cleaning, and analysing large datasets in Australia (e.g., PBS, MBS, VAED, VEMD) during his PhD studies at Monash University. He has also recently had experience analysing UK GP practice prescribing data from the Health & Social Care Information Centre.	☒	☐
**Is experience of practising in UK primary care available within the research team?**<br>*If yes, please indicate the name(s) of the relevant investigator(s)*<br>Rupert Payne<br>• Dr Rupert Payne is a practising GP and Consultant Senior Lecturer in Primary Care at the School of Social and Community Medicine, University of Bristol	☒	☐

12. References relating to your study

Please list up to 3 references (most relevant) relating to your proposed study:

- Domino ME, Olinick J, Sleath B, Leinwand S, Byrns PJ, Carey T. Restricting patients' medication supply to one month: Saving or wasting money. Am J Health-Syst Pharm 2004;61:1375-1379.

For peer review only - http://bmjopen.bmj.com/site/about/guidelines.xhtml

Section C: Access to the data			
**13. Financial Sponsor of study**			
Pharmaceutical Industry	☐ *Please specify:*	Academia	☒ *Please specify:* NIHR Health Technology Assessment Programme
Government / NHS	☐ *Please specify:*	Charity	☐ *Please specify:*
Other	☐ *Please specify:*	None	☐
**14. Type of Institution carrying out the analyses**			
Pharmaceutical Industry	☐ *Please specify:*	Academia	☒ *Please specify:* Cambridge Centre for Health Services Research, University of Cambridge
Government Department	☐ *Please specify:*	Research Service Provider	☐ *Please specify:*
NHS	☐ *Please specify:*	Other	☐ *Please specify:*

For peer review only

For peer review only - http://bmjopen.bmj.com/site/about/guidelines.xhtml

15. Data source

The sponsor has direct access to CPRD GOLD and will extract the relevant data* ☒

A data set will be supplied by CPRD** ☐

CPRD has been commissioned to extract the relevant data and to perform the analyses ☐
Other ☐ *Please specify:*

*If data sources other than CPRD GOLD are required, these will be supplied by CPRD
** Please note that datasets provided by CPRD are limited in size. Applicants should contact CPRD (KC@CPRD.com) if a dataset of >300,000 patients is required.

16. Primary care data (please specify which primary care data set(s) are required)
Vision only (Default for CPRD studies) ☒
EMIS® only* ☐
Both Vision and EMIS®* ☐

Note: Vision and EMIS are different clinical systems, Vision data has traditionally been used for CPRD, EMIS is currently undergoing beta-testing.
**Investigators requiring the use of EMIS data must discuss the study with a member of CPRD staff before submitting an ISAC application*
Please list below the name of the person/s at the CPRD with whom you have discussed your request for EMIS data:

Section D: Data linkage

17. Does this protocol also seek access to data held under the CPRD Data Linkage Scheme?

Yes* ☐ No ☒

If No, please move to section E.

**Investigators requiring linked data must discuss the study with a member of CPRD staff. It is important to be aware that linked data are not available for all patients in CPRD GOLD, the coverage periods for each data source may differ and charges may be applied. Please contact the CPRD Research Team on +44 (20) 3080 6383 or email kc@cprd.com to discuss your requirements before submitting your application.*
Please list below the name of the person/s at the CPRD with whom you have discussed your request:

Please note that as part of the ISAC review of linkages, the protocol may be shared - in confidence - with a representative of the requested linked data set(s) and summary details may be shared - in confidence - with the Confidentiality Advisory Group of the Health Research Authority.

For peer review only - http://bmjopen.bmj.com/site/about/guidelines.xhtml

18. Please select the source(s) of linked data being requested:

☐ ONS Mortality Data
☐ Inpatient Hospital Episode Statistics
☐ Outpatient Hospital Episode Statistics
☐ NCDR Cancer Registry Data*
☐ MINAP
☐ Mother Baby Link

☐ Index of Multiple Deprivation
☐ Townsend Score
☐ Other** *Please specify:*

Please note that applicants seeking access to cancer registry data must provide consent for publication of their study title and study institution on the UK Cancer Registry website. They must also complete a **Cancer Dataset Agreement Form (available from CPRD) and provide a **System level Security Policy** for each organisation involved in the study.*

*** If "Other" is specified, please name an individual in CPRD that this linage has been discussed with.*

19. Total number of linked datasets requested including CPRD GOLD:

20. Is linkage to a local dataset with <1 million patients being requested?

Yes* ☐ No ☐

** If yes, please provide further details:*

21. If you have requested linked data sets, please indicate whether the Chief Investigator or any of the collaborators listed in response to question 5 above, have access to any of the linked datasets in a patient identifiable form, or associated with a patient index.

Yes* ☐ No ☐

** If yes, please provide further details:*

22. Does this study involve linking to patient *identifiable* data from other sources?

Yes ☐ No ☐

Section E: Validation/verification

23. Does this protocol describe a purely observational study using CPRD data (this may include the review of anonymised free text)?

Yes* ☒ No** ☐

** Yes: If you will be using data obtained from the CPRD Group, this study does not require separate ethics approval from an NHS Research Ethics Committee.*
*** No: You may need to seek separate ethics approval from an NHS Research Ethics Committee for this study. The ISAC will provide advice on whether this may be needed.*

24. Does this study require anonymised free text?

Yes* ☐ No ☒

**Please note that work involving free text can only be performed on the July 2013 CPRD GOLD database build or earlier versions. CPRD can provide further advice on the use of anonymised free text.*

25. Does this protocol involve requesting any additional information from GPs?

Yes* ☐ No ☒

** Please indicate what will be required:*
Completion of questionnaires by the GP^ψ^ Yes ☐ No ☐
Provision of anonymised records (e.g. hospital discharge summaries) Yes ☐ No ☐
Other (please describe)

ψ Any questionnaire for completion by GPs or other health care professional must be approved by ISAC before circulation for completion.

For peer review only - http://bmjopen.bmj.com/site/about/guidelines.xhtml

**26. Does this study require contact with patients in order for them to complete a questionnaire?**			
Yes*	☐	No	☒
**Please note that any questionnaire for completion by patients must be approved by ISAC before circulation for completion.*			
**27. Does this study require contact with patients in order to collect a sample?**			
Yes*	☐	No	☒
** Please state what will be collected:*			
**Section F: Signatures**			
**28. Signature from the Chief Investigator**			
I confirm that the above information is to the best of my knowledge accurate, and I have read and understood the guidance to applicants.			
Name: Ed Wilson	Date: 25/05/16	E. signature (type name):	ED WILSON

For peer review only

PROTOCOL INFORMATION

In order to help ensure that protocols submitted for review contain adequate information for protocol evaluation, ISAC have produced guidance on the content of protocols for research using CPRD data. This guidance is available on the CPRD website (www.cprd.com/ISAC). All protocols using CPRD data which are submitted for review by ISAC must contain information on all the areas detailed below. If a specific area required by ISAC is not applicable to your protocol, please provide the justification underneath the relevant heading.

The protocol section (next page) has pre-defined headings and the protocol must be written using these headings. Additional headings are not acceptable; however, supplementary information may be placed in one or more of the appendices providing this information is essential and an appropriate reference to it is made within the protocol. Unless very short, codes lists should be placed in an Appendix. Applications will be regarded as invalid and returned to the applicant if any of the headings below are missing or if additional sections are included.

Please note that ISAC will not consider any application where the protocol exceeds 12 pages (excluding sections A-F of the application form and annexes). Annexes should be kept to a minimum and contain only vital information that could not be provided in the main protocol section. A font-size of at least 12 should be used. Protocols not exceeding 15 pages would be acceptable if ISAC has required a resubmission where additional information is requested.

Please note, your protocol will not be reviewed by ISAC if it falls short of the above requirements. You are advised to speak to the Secretariat if you have any queries.

Voluntary registration of ISAC approved studies:

Epidemiological studies are increasingly being included in registries of research around the world, including those primarily set up for clinical trials. To increase awareness amongst researchers of ongoing research, ISAC encourages voluntary registration of epidemiological research conducted using MHRA databases. This will not replace information on ISAC approved protocols that may be published on the CPRD website. It is for the applicant to determine the most appropriate registry for their study. Please inform the ISAC secretariat that you have registered a protocol and provide the location.

For peer review only - http://bmjopen.bmj.com/site/about/guidelines.xhtml

Protocol Section

The following headings **must** be used to form the basis of the protocol. Pages should be numbered. All abbreviations must be defined on first use.

A. Lay Summary (Max. 200 words)

Please provide a succinct overview of your proposed research in plain English i.e. non-technical language. This should cover the background, purpose of the study and the potential importance of the findings. References and abbreviations should be avoided. If you have ticked the "other" box in response to question 4 on the application form, up to an additional 100 words should be used to describe the benefit to public health expected from the study.

In the NHS, general practitioners (GPs) have been encouraged to issue prescriptions of shorter duration (e.g., 28 days), to reduce drug expenditure and wastage. There is, however, the potential for shorter prescriptions to increase costs through increased GP workload and dispensing fees. Currently, the consequences of longer and shorter prescriptions for patients with chronic diseases are unknown and need to be assessed. The purpose of this study is to determine the if there are differences in the costs related to drug wastage, dispensing fees and prescriber time as a result of early refills and treatment switches for prescriptions issued as either a 28-day or 3-month supply in five selected case study scenarios representing common chronic conditions. This study will provide important information to the Department of Health in understanding the impact that encouraging GPs to issue shorter supplies of drugs has had on drug expenditure and drug wastage and additionally, help inform future prescribing policies.

B. Technical Summary (Max. 200 words)

Please provide a succinct overview of the objectives, methods and data analysis for the proposed research. Avoid the use of references in this section.

The aim of this study is to estimate the differences in the costs of drug wastage, dispensing fees and prescriber time as a result of early refills and treatment switches in patients receiving medications as either 28-day or 3-month supplies for a number of common chronic diseases. A retrospective cohort analysis will be conducted using data from a random sample of 50,000 patients for five case study conditions derived from all adult patients receiving at least one prescription relevant to the respective condition during the 10-year period between 2004 and 2014. The volume of wastage from early refills and treatment switches (defined as a repeat prescription or new prescription for a drug commonly prescribed for the same condition being issued prior to the expiry of the previously prescribed quantity) will be estimated. Unit costs from standard sources will be applied to estimate the cost of wastage and dispensing for a common price year. The cost of health professional time to issue the prescription will also be added. Changes in drug wastage and dispensing fees will then be estimated had all prescriptions been for 28 days rather than the observed length.

C. Objectives, Specific Aims and Rationale

Please include:
(i) The broad research objectives
(ii) The specific aims; any hypotheses to be tested should be stated here.
(iii) An explanation of how achievement of the specific aims will further the research objectives

For peer review only - http://bmjopen.bmj.com/site/about/guidelines.xhtml

The broad research objectives of the entire research project (note that the proposed study within this application is only one component of a larger NIHR-funded HTA project) are to assess whether shorter (28 day) or longer (3 month) prescription lengths have an impact on medication wastage, dispensing costs and health professional prescriber time.

The aims of the component of the study for which we require CPRD data are to investigate the patterns of treatment switching and early refills over a 10-year period in order to estimate differences in the cost of drug wastage, dispensing fees and health professional prescriber time for 28-day and 3-month prescription lengths.

The results of the study will provide evidence to guide policy on the optimal choice of prescription length based on the potential economic implications of different policy scenarios.

D. Background

Please provide a succinct review of the relevant background literature with references so as to explain the purpose of the study. Please ensure that you refer to any previous research in CPRD that is related, providing published references and, when known, the ISAC Protocol Number

In an effort to reduce expenditure on, and wastage of, drugs some commissioners have encouraged GPs to issue shorter prescriptions, typically 28 days in length.[NHS Cambridgeshire, 2009; NHS Dorset Clinical Commissioning Group, 2013] The rationale being, to strike a balance between patient convenience, good medical practice and drug wastage. It has been estimated that between £100 million and £300 million worth of prescriptions dispensed in the community was wasted in 2007 and 2009.[Trueman P et al., 2010] Some evidence suggests that this wastage could be reduced if prescriptions were limited to a 28 day supply.[Hawksworth GM et al., 1996]

Shorter prescriptions, however, may increase the costs to the healthcare system through increased GP workload and dispensing costs to pharmacists. Recent evidence suggested that dispensing fees, as a result of increased numbers of shorter prescriptions, cost the NHS approximately £150 million in 2009.[Wilson PM et al., 2013] If all 842.5 million prescription items dispensed in the community in England in 2008 had been 28-day repeats, dispensing fees would have been 50% higher (£700 million increase on £1.5 billion current expenditure).[White KG et al., 2010] This same conclusion followed from a simulation model published in 2004 comparing 100-day with 34-day supplies in a US Medicaid setting:[Domino ME et al., 2004] shorter prescription lengths were associated with a reduction in drug wastage of 5-14%. However, increases in dispensing fees more than exceeded this decrease in drug wastage.

Given the disparity and lack of evidence from the perspective of the NHS in the UK, it is clear that an analysis is required to assess the impact of prescription length on costs to health services in terms of wastage, dispensing fees and health professional prescriber time.

E. Study Type

Specify whether the study will be primarily descriptive, exploratory, hypothesis testing or a methodological piece of research.

For peer review only - http://bmjopen.bmj.com/site/about/guidelines.xhtml

Descriptive analysis.

F. Study Design

Describe the overall research design (for example, case-control, cohort) and reasons for choosing the proposed study design.

This study will be a retrospective cohort study of a random sample of 50,000 patients for each of the five case study conditions (see section K) derived from all adult (≥18 years old) patients receiving at least one prescription relevant to the respective condition (see Appendix 1) during the 10-year period between January 1, 2004 and December 31, 2014. The 10-year study period has been chosen to ensure a sufficient number of treatment switches (specifically switches between drugs that are in the same class or different classes, but therapeutically related) are observed as these may happen relatively infrequently over the course of treating some chronic diseases. Prescribing data over the 10-year period will be studied. Descriptive analyses of trends in treatment switching and early refills will be carried out for each annual period and 10-years overall.

G. Sample Size

Please provide an estimate of sample size, and, where possible, a formal power calculation. An estimate of the expected number of patients available in the CPRD database should normally be included.

A random sample of 50,000 patients for each of the five case study conditions (see section K) derived from all adult (≥18 years old) patients receiving at least one prescription relevant to the respective condition (see Appendix 1) during the 10-year period between 2004 and 2014 will be included. If we assume on average five years of follow up are available for a patient in CPRD [Herrett E et al., 2015] and that patients may be issued the prescriptions of interest for half that time (note this may be different depending on the condition of interest, but has been used as a lower limit) and that patients are likely to receive between 4 and 12 prescriptions per year (based on dispensing of either a one month or three month supply) than overall, patients in CPRD are likely to have between 10 and 30 prescriptions related to the conditions of interest. A random sample of 50,000 patients would result in roughly 500,000 to 1.5 million prescriptions in total. Given previously reported annual proportions of treatment switches for angiotensin-converting enzyme inhibitors (2.6%), sulfonylureas (0.8%) and selective serotonin reuptake inhibitors (1.0%) [Domino ME et al. 2004] and if we look to assess the number of switches each year over our 10-year study period, assuming the number of prescriptions is equally spread over the 10 years (~42,000/year) for a sample of 50,000 (lower limit 500,000 prescriptions in total) we would expect to detect these proportions of switches with acceptable precision [0.01 95% CI (0.0090705, 0.0109981) and 0.03 95% CI (0.0283892, 0.0316762)].

H. Data Linkage Required (if applicable)

Please provide a synopsis of the purpose(s) for which the each of the linkages requested in section 18 of the application form is required.

Not applicable.

I. Study Population

For peer review only - http://bmjopen.bmj.com/site/about/guidelines.xhtml

Define the source and study population, in terms of persons, place, time period, and listing the criteria which will be used to select the study population from the CPRD, i.e any inclusion or exclusion criteria. Please make clear any restrictions imposed by the use of linked datasets.

A random sample of 50,000 patients for each of the five case study conditions (see section K) derived from all adult (≥18 years old) patients receiving at least one prescription relevant to the respective condition (see Appendix 1) during the 10-year period between January 1, 2004 and December 31, 2014 will be included.

J. Selection of comparison group(s) or controls

Describe the criteria for eligibility and the procedure for control selection.

Not applicable.

K. Exposures, Outcomes and Covariates

For exposures and outcomes operational definitions (or procedures for developing them) must be provided, supported by preliminary code lists placed in an Annex. A comprehensive list of covariates should also be provided for any study which is not purely descriptive.

Five case study conditions were selected based on their frequency of occurrence within the population and the potential for a variety of expected frequencies in prescription changes over the course of treatment for each condition.

A list of medications routinely prescribed for the selected case study conditions was identified by review of appropriate clinical guidelines and consultation with clinical colleagues.

The five case study conditions are:

1) glucose control in type II diabetes (patients receiving at least one prescription for an anti-diabetic drug listed under ‘BNF 6.1.2 Antidiabetic drugs’);

2) primary prevention of hypertension in type II diabetes (in addition to receiving an anti-diabetic drug as defined in (1), patients receiving at least one prescription for a medication used for the primary prevention of hypertension in type II diabetes patients, including angiotensin-converting enzyme inhibitors, angiotensin II receptor antagonists, calcium-channel blockers and thiazide-like diuretics);

3) primary prevention of hyperlipidaemia in type II diabetes (in addition to receiving an anti-diabetic drug as defined in (1), patients receiving at least one prescription for a statin used for the primary prevention of hyperlipidaemia in type II diabetes patients;

4) secondary prevention of myocardial infraction (in addition to receiving concurrent prescriptions for a angiotensin-converting enzyme inhibitor, antiplatelet and statin for at least one year in duration, patients may also receive prescriptions for beta-adrenoceptor blockers, calcium-channel blockers, oral anticoagulants and aldosterone antagonists);

For peer review only - http://bmjopen.bmj.com/site/about/guidelines.xhtml

5) and depression (patients receiving at least one prescription for an anti-depressant drug listed under 'BNF 4.3 Antidepressant drugs').

Preliminary product code lists of potentially prescribed medications for each of the five case study conditions are provided in Appendix 1.

L. Data/ Statistical analysis

This section should cover both the analytic methods and also the analyses which are to be performed to meet all the specific aims listed earlier. It is important to ensure that this section is clear and specific about any comparisons which will be made.

For each of the five case study conditions the associated product codes listed in Appendix I will first be reviewed to create groups of similar products, where possible. Next, all possible substitutions between the available products will be mapped and will include:

1)substitutions between different dosages or formulations of the same drug substance (active ingredient);
2) substitutions between drugs within the same class (e.g., switch between two different statins);
3) and substitutions between drugs that are therapeutically related (e.g., switch from angiotensin-converting enzyme inhibitor to calcium-channel blocker)

We will then estimate the volume of medication wastage from early refills and treatment switches (defined as a repeat prescription or new prescription based on the mapped substitutions outlined above respectively, being issued prior to the expiry of the previously prescribed quantity). This can be estimated by dividing the total quantity entered by the GP for the prescribed product (qty) by the numeric daily dose prescribed for the event (ndd) and comparing this to the difference in the two dates associated with the events, as entered by the GP (eventdate). A threshold of one year after the initial prescription in a particular series will be used to estimate wastage for early refills (i.e., repeat prescriptions), in that any product prescribed over and above the expected quantity to be consumed within the one year time period will be considered waste. This is to account for the fact that some patients may fill their prescriptions before their existing supply is exhausted, but still consume all of the previous prescription before using the new supply. Therefore, if we assumed wastage for all early refills we may be overestimating the impact.

In contrast, for treatment switches we will assume any additional product not consumed before the switch date will be considered waste. There are, however, two exceptions: 1) prescriptions issued on the same day for drugs in the same class (e.g., two different statins) will be considered prescriber error and drop from the analysis as it is unlikely that prescriptions for drugs in the same class would be issued on the same day; and 2) to differentiate between add-ons and switches, particularly for therapeutically related drugs we will only define an overlap of prescriptions dates as wastage due to a treatment switch if there is not another prescription issued for the original product within a three month time period. The three month threshold has been chosen to ensure prescriptions issued for both one and three month periods are captured. The three month threshold will also be altered in sensitivity analysis to test the robustness of this assumption.

For peer review only - http://bmjopen.bmj.com/site/about/guidelines.xhtml

Alternatively, we may chose not to differentiate between actual medication wastage due to switches and the augmentation of medication through add-ons as well as count any overlapping dates as wastage for early refills. Under this scenario we may overstate the amount of wastage that occurred, but this can be considered a conservative assumption, as it puts an upper bound on the savings that would occur if premature medication switches could be eliminated entirely.

The cost of wastage can then be estimated by applying net ingredient costs (NICs) obtained from national general practice prescribing data provided by the Health & Social Care Information Centre for the respective prescription to the estimated quantity of waste. BNF codes will be used to link the NICs from the general prescribing data to CPRD. Since BNF codes from CPRD are limited to only a 7 digit numeric code representing chapter, section, paragraph and subparagraph (i.e., does not provide 8 digit alpha/numeric code representing the drug substance and specific formulation) it is necessary to make some additional assumptions to link the datasets. First, total NIC will be divided by total number of items prescribed and dispensed for each product listed to obtain a NIC per item. A weighted average using total quantities of all the NICs per item for a particular BNF paragraph will be calculated. For example, BNF code 0403010 in CPRD represents Chapter 4 “Central nervous system”, Section 3 “Antidepressant drugs”, Paragraph 1 “Tricyclic and related antidepressant drugs” and Subparagraph 0, which means the BNF does not extend to the subparagraph level in this case. Therefore a weighted average of all the NICs in Paragraph 1 “Tricyclic and related antidepressant drugs” will be applied to any drug falling in this category (e.g., amitriptyline, clomipramine, dosulepin, doxepin, imipramine, etc.).

Based on the number of prescriptions, dispensing fees related to each prescription from the Drug Tariff and the estimated cost of health professional prescriber time based on the literature can be added to the cost of wastage to determine the total cost from a NHS perspective. A targeted literature review will be designed to determine the time involved for a health professional to issue a prescription. Note this may be different depending on the type of health professional (e.g., general practitioner versus nurse), but this will be tested in sensitivity analysis. Hourly costs related to the health professionals’ time, derived from the PSSRU’s Unit Costs of Health & Social Care will then be applied.[Curtis L and Burns A, 2015]

Scenario analysis will then be conducted, estimating changes in drug wastage and dispensing fees if all prescriptions had been for 28 days rather than the observed length. Appropriate sensitivity analyses will also be conducted, for example, around the cost of health professional prescriber time required to issue a repeat prescription.

M. Plan for addressing confounding

Purely descriptive studies are exempt from this requirement. All other studies should here provide some discussion of what they are doing in the design and/or analysis to control for confounding.

Not applicable.

N. Plan for addressing missing data

The potential for missing data should be identified and how it will be addressed discussed here.

For peer review only - http://bmjopen.bmj.com/site/about/guidelines.xhtml

Missing data in the CPRD therapy file should not be a major issue. Our analysis does, however, rely on the use of the numeric daily dose (ndd) variable. As this variable is derived using a CPRD algorithm on common dosage strings there is the potential for it to be equal to zero in cases of a non-numeric textid (e.g., if the textid refers to say “apply as needed”). This type of textid is unlikely for the medications chosen to be included in our analysis and therefore our analysis will be limited to only those observations with a complete case (i.e., ndd is not missing or equal to zero and quantity is not missing). This seems to be acceptable as this approach has been employed in other similar CPRD studies using these two variables.[Brodie MJ et al., 2016 and Francis NA et al., 2016]

O. Limitations of the study design, data sources and analytical methods

The general limitations of the databases and observational research are well-known. Specific consideration of the potential impact of such limitations should be provided in the context of the proposed study.

The key limitations specific to this protocol are as follows:

1. To define three of the five case study conditions (see section K; conditions 2, 3 and 4) based only on the available prescription data from CPRD it was necessary to make assumptions regarding the population’s composition. For example, to define a population receiving medication for the secondary prevention myocardial infraction it was necessary to assume (based on clinical guidelines) that patients receiving a angiotensin-converting enzyme inhibitor, antiplatelet and statin for at least one year in duration had previously had a myocardial infraction.
2. From the data we will not be able to differentiate between repeat prescriptions and a number of acute prescriptions. Therefore to avoid overestimating wastage we have proposed to use a threshold of one year after the initial prescription in a particular series to estimate wastage for early refills (i.e., repeat prescriptions), in that any product prescribed over and above the expected quantity to be consumed within the one year time period will be considered waste.
3. Five case study conditions were purposively rather than randomly selected to represent the impact of medication refill and switching behaviour, but they may not be representative of prescribing behaviour in other chronic conditions. The conditions do, however, represent some of the most common chronic conditions treated with prescribed medications.
4. Our estimates of drug wastage will not account for imperfect adherence and therefore might represent an underestimate of the true quantity and cost of medication wastage. However, an additional aspect of this project (being conducted by other colleagues under the same NIHR HTA proposal) will attempt to quantify the impact of imperfect adherence for both long and short prescription lengths.
5. NICs used to estimate the cost of wastage are the prices listed on the Drug Tariff or if not on the tariff, the list prices published by the manufacturer. NICs do not include any discounts that may be applied. NICs also do not include any adjustment for income obtained where a prescription charge is paid at the time the prescription is dispensed or where the patient has purchased a pre-payment certificate. However, NICs are the only linkable source of prescription drug unit for large datasets like CPRD.
6. BNF codes will be used to link the NICs from the general prescribing data to CPRD. Since BNF codes from CPRD are limited to only a 7 digit numeric code representing chapter, section, paragraph and subparagraph (i.e., does not provide 8 digit alpha/numeric code representing the drug substance and specific formulation) it is

For peer review only - http://bmjopen.bmj.com/site/about/guidelines.xhtml

necessary to make some additional assumptions to link the datasets. First, total NIC will be divided by total number of items prescribed and dispensed for each product listed to obtain a NIC per item. A weighted average using total quantities of all the NICs per item for a particular BNF subparagraph will be calculated.

7. A main limitation of CPRD prescription data is that it does not indicate whether or not a medication has been dispensed or whether patients took their prescribed medications as recommended (i.e., it only indicate when a prescription has been issued). Therefore, our estimates may have overstated the amount of wastage that actually occurred. This, however, is a conservative assumption and can be seen as an upper bound on the savings that would occur if drug wastage from premature medication switches could be eliminated entirely.

P. Patient or user group involvement (if applicable)

Please indicate whether you have or intend to involve patient groups in your study. Such involvement is encouraged by ISAC and required for studies which directly involve patients.

In preparation for this proposal, we sent an outline of our proposed research to members of INsPIRE, a patient and public involvement panel for Bedfordshire and Cambridgeshire. Email comments were sent back from seven panel members. Five of the members stressed the importance of this research and six members maintained that three month prescriptions were preferable to 28 day prescriptions for chronic conditions. However, one member cautioned that 28 day prescriptions may be suitable for 'concern medications', such as sleeping pills. Six of the respondents mentioned the additional cost of shorter duration prescriptions, which they described in terms of drug wastage, patient time, GP time and prescription fees. Two members also mentioned the importance of synchronisation of prescriptions for patients with multiple co-morbidities. Finally, two members stressed the importance of focusing on individual patient needs when prescribing medications. During the writing of the proposal, we have taken these views into account.

Patients and the public were involved in the design of the research and will be involved in the dissemination of research findings.

Q. Plans for disseminating and communicating study results, including the presence or absence of any restrictions on the extent and timing of publication

ISAC expects most studies that it approves to be published in the scientific literature and considers it an ethical obligation for any study with potential public health implications. In cases where multiple publications are likely to arise, a publication plan should be provided in this section.

The primary audience for the proposed research will be policy makers, those who manage and provide care for patients with long-term stable chronic conditions (i.e., general practitioners and pharmacists), as well as patient groups with stable, chronic conditions who require regular repeat prescriptions. In addition to a HTA monograph, we plan to publish the findings in an academic peer-reviewed journal and present the findings at relevant academic conferences. Our patients and public involvement members will be asked to assist in the production of a short summary for non-technical audiences.

R. References

For peer review only - http://bmjopen.bmj.com/site/about/guidelines.xhtml

Please provide a numbered list of references at the end of the protocol.

Brodie MJ, Chung S, Wade A, Quelen C, Guiraud-Diawara A, François C, Verpillat P, Shen V, Isojarvi Clobazam and clonazepam use in epilepsy: Results from a UK database incident user cohort study. Epilepsy Research 2016;123:68-74.

Curtis L, Burns A. Personal Social Services Research Unit – Unit Costs of Health & Social Care 2015. The University of Kent. Canterbury.

Domino ME, Olinick J, Sleath B, Leinwand S, Byrns PJ, Carey T. Restricting patients' medication supply to one month: Saving or wasting money? Am J Health-Syst Pharm 2004;61:1375-1379.

Francis NA, Hood K, Lyons R, Butler CC. Understanding flucloxacillin prescribing trends and treatment non-response in UK primary care: a Clinical Practice Research Datalink (CPRD) study. J Antimicrob Chemother 2016 Apr 18.

Hawksworth GM, Wright D, Chrystyn H. A detailed analysis of the day to day unwanted medicinal products returned to community pharmacies for disposal. Journal of social and administrative pharmacy 1996;13:215-222.

Herrett E, Gallagher AM, Bhaskaran K, Forbes H, mathur R, van Staa T, Smeeth L. Data Resource Profile: Clinical Practice Research Datalink (CPRD). International Journal of Epidemiology 2015;44(3):827-836.

NHS Cambridgeshire. Repeat Medication for 28 Days. 2009

NHS Dorset Clinical Commissioning Group. Medicines Code Chapter 15: Policy for Repeat Prescribing and Medication Review. 2013

Trueman P, Lowson K, Blighe A et al. Evaluation of the Scale, Causes and Costs of Waste Medicines. York & London: York Health Economics Consortium & The School of Pharmacy, University of London, 2010.

White KG. UK interventions to control medicines wastage: a critical review. Int J Pharm Pract 2010;18(3):131-40.

Wilson PM, Kataria N, McNeilly E. patient and carer experience of obtaining regular prescribed medication for chronic disease in the English National Health Service: a qualitative study. BMC health services research 2013;13(1):192.

Appendices

Appendices should be used for essential supporting information only (e.g. code-lists) and they must be cited within the body of the protocol.

Please see accompanying document:

- Appendix 1: Preliminary product codes lists for each of the five case study conditions of interest (Excel file)

For peer review only - http://bmjopen.bmj.com/site/about/guidelines.xhtml

Appendix VIII - Comparison of medication wastage over 11-year period 2004-2014

	Proportion of days' supply wasted % (95% CI)		Mean number of days' supply wasted days (95% CI)		Mean cost of wastage per prescription 2015 £ (95% CI)	
	**<60 days**	**≥60 days**	**<60 days**	**≥60 days**	**<60 days**	**≥60 days**
Glucose control with oral drug therapy in T2DM	2.658 (2.641-2.675	4.920 (4.822-5.018)	0.859 (0.853-0.865)	4.962 (4.044-5.880)	0.329 (0.325-0.333)	1.370 (1.104-1.636)
Hypertension in T2DM	3.762 (3.747-3.777)	5.011 (4.935-5.087)	1.232 (1.227-1.237)	6.979 (5.956-8.002)	0.095 (0.094-0.096)	0.437 (0.389-0.486)
Lipid management in T2DM	1.652 (2.640-1.665)	4.071 (3.966-4.177)	0.628 (0.623-0.633)	16.211 (12.979-19.443)	0.048 (0.048-0.049)	1.426 (1.132-1.720)
Secondary prevention of myocardial infraction	3.325 (3.315-3.335)	3.663 (3.612-3.714)	0.956 (0.953-0.959)	6.557 (5.761-7.353)	0.066 (0.066-0.066)	0.510 (0.370-0.649)
Depression	6.340 (6.157-6.385)	3.663 (3.535-3.792)	1.866 (1.852-1.881)	2.695 (2.592-2.797)	0.207 (0.203-0.212)	0.429 (0.977-0.480)

CI – confidence interval; T2DM – type II diabetes mellitus

For peer review only

For peer review only - http://bmjopen.bmj.com/site/about/guidelines.xhtml

Appendix IX - Comparison of the mean cost of medication wastage per prescription each year from 2004 to 2014 (2015 £)

	Glucose control with oral drug therapy in T2DM 2015 £ (95% CI)		Hypertension in T2DM 2015 £ (95% CI)		Lipid management in T2DM 2015 £ (95% CI)		Secondary prevention of myocardial infraction 2015 £ (95% CI)		Depression 2015 £ (95% CI)	
**Year**	**<60 days**	**≥60 days**	**<60 days**	**≥60 days**	**<60 days**	**≥60 days**	**<60 days**	**≥60 days**	**<60 days**	**≥60 days**
2004	0.381 (0.364-0.397)	1.813 (0.574-3.052)	0.127 (0.122-0.131)	0.462 (0.351-0.573)	0.068 (0.065-0.070)	1.511 (0.599-2.422)	0.085 (0.084-0.087)	0.376 (0.175-0.578)	0.247 (0.227-0.266)	0.428 (0.308-0.548)
2005	0.423 (0.405-0.441)	1.331 (1.080-1.582)	0.123 (0.118-0.127)	0.525 (0.386-0.663)	0.055 (0.053-0.058)	2.363 (1.118-3.607)	0.082 (0.081-0.084)	0.521 (0.321-0.721)	0.221 (0.204-0.238)	0.452 (0.296-0.608)
2006	0.418 (0.400-0.435)	1.045 (0.906-1.185)	0.111 (0.107-0.114)	0.428 (0.328-0.529)	0.057 (0.055-0.060)	2.274 (1.059-3.488)	0.079 (0.077-0.080)	1.277 (0.132-2.421)	0.222 (0.205-0.239)	0.579 (0.359-0.800)
2007	0.387 (0.370-0.403)	1.110 (0.956-1.264)	0.101 (0.098-0.104)	0.467 (0.325-0.608)	0.050 (0.048-0.052)	1.533 (0.557-2.509)	0.072 (0.070-0.073)	0.400 (0.0240-0.559)	0.222 (0.205-0.239)	0.376 (0.247-0.505)
2008	0.343 (0.329-0.357)	1.086 (0.922-1.249)	0.096 (0.093-0.099)	0.530 (0.314-0.746)	0.045 (0.043-0.048)	1.663 (0.600-2.726)	0.065 (0.065-0.067)	0.457 (0.259-0.654)	0.207 (0.192-0.221)	0.359 (0.227-0.491)
2009	0.299 (0.287-0.311)	1.074 (0.849-1.298)	0.091 (0.088-0.093)	0.472 (0.282-0.662)	0.048 (0.046-0.051)	1.717 (0.638-2.796)	0.063 (0.062-0.064)	0.528 (0.302-0.754)	0.206 (0.191-0.220)	0.421 (0.243-0.599)
2010	0.330 (0.317-0.342)	1.396 (0.744-2.048)	0.085 (0.082-0.087)	0.379 (0.213-0.545)	0.046 (0.043-0.048)	0.991 (0.245-1.738)	0.061 (0.060-0.062)	0.477 (0.253-0.701)	0.183 (0.171-0.195)	0.366 (0.216-0.516)
2011	0.263 (0.254-0.272)	3.139 (0.659-5.620)	0.082 (0.080-0.085)	0.477 (0.259-0.695)	0.042 (0.040-0.044)	0.879 (0.154-1.605)	0.057 (0.056-0.058)	0.409 (0.208-0.611)	0.173 (0.162-0.183)	0.383 (0.239-0.528)
2012	0.251 (0.243-0.260)	0.932 (0.820-1.043)	0.078 (0.075-0.080)	0.402 (0.208-0.596)	0.045 (0.043-0.047)	0.740 (0.135-1.346)	0.056 (0.055-0.057)	0.354 (0.192-0.516)	0.182 (0.169-0.195)	0.350 (0.171-0.528)
2013	0.268 (0.258-0.277)	0.871 (0.758-0.984)	0.074 (0.072-0.076)	0.232 (0.189-0.275)	0.038 (0.036-0.039)	0.448 (0.063-0.834)	0.053 (0.052-0.054)	0.200 (0.131-0.268)	0.199 (0.186-0.213)	0.436 (0.189-0.683)
2014	0.301 (0.290-0.311)	1.168 (1.018-1.318)	0.079 (0.076-0.082)	0.271 (0.202-0.340)	0.047 (0.045-0.050)	0.796 (0.102-1.489)	0.054 (0.053-0.056)	0.188 (0.103-0.273)	0.233 (0.217-0.249)	0.593 (0.374-0.812)

NS – not significant at p<0.05 level; T2DM – type II diabetes mellitus

For peer review only

For peer review only - http://bmjopen.bmj.com/site/about/guidelines.xhtml

Appendix X – Differences in standardised (120 day) total unnecessary costs for short and long prescription length under various scenarios (2015 £)

	Values tested		Total unnecessary cost standardised to 120 days		Difference (Cost savings with ≥60 day)
Scenarios	**<60 days**	**≥60 days**	**<60 days**	**≥60 days**	
**Initial glucose control in type II diabetes**					
Base case	-	-	13.24	4.86	(8.38)
Nurse prescriber time cost, not GP	2.28	2.29	10.13	4.19	(5.94)
No prescriber time cost	0	0	3.76	2.85	(0.91)
Addition of prescription charge (loss in NHS revenue)	0.84	0.84	10.89	4.37	(6.52)
50% decrease quantity wasted, days	0.43	2.48	12.65	3.74	(8.91)
50% increase quantity wasted, days	1.29	7.44	13.84	5.98	(7.86)
50% decreased cost of drug per day	0.18	0.14	12.65	3.74	(8.91)
50% increase cost of drug per day	0.55	0.43	13.84	5.98	(7.86)
50% decrease dispensing fee	0.46	0.52	11.96	4.55	(7.41)
50% increase dispensing fee	1.38	1.57	14.53	5.16	(9.37)
50% decrease prescriber time cost	1.70	1.72	8.50	3.85	(4.65)
50% increase prescriber time cost	5.09	5.16	17.99	5.86	(12.13)
**Hypertension in type II diabetes**					
Base case	-	-	12.32	2.50	(9.82)
Nurse prescriber time cost, not GP	2.31	2.32	9.04	2.03	(7.01)
No prescriber time cost	0	0	2.81	1.15	(1.66)
Addition of prescription charge (loss in NHS revenue)	0.84	0.84	10.06	2.18	(7.88)
50% decrease quantity wasted, days	0.62	3.49	12.13	2.10	(10.03)
50% increase quantity wasted, days	1.85	10.47	12.52	2.89	(9.63)
50% decreased cost of drug per day	0.042	0.041	12.13	2.10	(10.03)

For peer review only - http://bmjopen.bmj.com/site/about/guidelines.xhtml

	Values tested		Total unnecessary cost standardised to 120 days		Difference (Cost savings with ≥60 day)
Scenarios	<60 days	≥60 days	<60 days	≥60 days	
50% increase cost of drug per day	0.13	0.12	12.52	2.89	(9.63)
50% decrease dispensing fee	0.45	0.46	11.11	2.32	(8.79)
50% increase dispensing fee	1.35	1.39	13.54	2.67	(10.87)
50% decrease prescriber time cost	1.77	1.77	7.57	1.82	(5.75)
50% increase prescriber time cost	5.30	5.32	17.08	3.17	(13.91)
**Hyperlipidaemia in type II diabetes**					
Base case	-	-	10.95	1.54	(9.41)
Nurse prescriber time cost, not GP	2.21	2.22	8.54	1.56	(6.98)
No prescriber time cost	0	0	2.64	1.61	(1.03)
Addition of prescription charge (loss in NHS revenue)	0.84	0.84	8.71	1.56	(7.15)
50% decrease quantity wasted, days	0.31	8.11	10.83	0.73	(10.10)
50% increase quantity wasted, days	0.94	24.32	11.07	2.36	(8.71)
50% decreased cost of drug per day	0.052	0.052	10.83	0.73	(10.10)
50% increase cost of drug per day	0.16	0.15	11.07	2.36	(8.71)
50% decrease dispensing fee	0.45	0.50	9.75	1.55	(8.20)
50% increase dispensing fee	1.35	1.50	12.15	1.53	(10.62)
50% decrease prescriber time cost	1.56	1.57	6.79	1.58	(5.21)
50% increase prescriber time cost	4.68	4.72	15.11	1.51	(13.60)
**Secondary prevention of myocardial infraction**					
Base case	-	-	13.40	1.34	(12.06)
Nurse prescriber time cost, not GP	2.37	2.37	9.49	1.10	(8.39)
No prescriber time cost	0	0	2.81	0.69	(2.12)
Addition of prescription charge (loss in NHS revenue)	0.84	0.84	11.03	1.20	(9.83)

For peer review only - http://bmjopen.bmj.com/site/about/guidelines.xhtml

	Values tested		Total unnecessary cost standardised to 120 days		Difference (Cost savings with ≥60 day)
Scenarios	<60 days	≥60 days	<60 days	≥60 days	
50% decrease quantity wasted, days	0.48	3.28	13.27	1.08	(12.19)
50% increase quantity wasted, days	1.43	9.84	13.54	1.60	(11.94)
50% decreased cost of drug per day	0.037	0.034	13.27	1.08	(12.19)
50% increase cost of drug per day	0.11	0.10	13.54	1.60	(11.94)
50% decrease dispensing fee	0.45	0.48	12.13	1.26	(10.87)
50% increase dispensing fee	1.35	1.45	14.67	1.43	(13.24)
50% decrease prescriber time cost	1.88	1.89	8.11	1.02	(7.09)
50% increase prescriber time cost	5.63	5.66	18.70	1.67	(17.03)
**Depression**					
Base case	-	-	15.06	4.04	(11.02)
Nurse prescriber time cost, not GP	2.24	2.23	11.72	3.24	(8.48)
No prescriber time cost	0	0	4.16	1.35	(2.81)
Addition of prescription charge (loss in NHS revenue)	0.84	0.84	12.22	3.33	(8.89)
50% decrease quantity wasted, days	0.93	1.35	14.51	3.77	(10.74)
50% increase quantity wasted, days	2.80	4.04	15.61	4.31	(11.30)
50% decreased cost of drug per day	0.068	0.054	14.51	3.77	(10.74)
50% increase cost of drug per day	0.20	0.16	15.61	4.31	(11.30)
50% decrease dispensing fee	0.45	0.48	13.53	3.64	(9.89)
50% increase dispensing fee	1.36	1.44	16.59	4.45	(12.14)
50% decrease prescriber time cost	1.61	1.59	9.61	2.70	(6.91)
50% increase prescriber time cost	4.84	4.78	20.51	5.39	(15.12)

For peer review only

REFERENCES FOR APPENDICIES

1. Taitel M, Fensterheim L, Kirkham H, et al. Medication Days' Supply, Adherence, Wastage, and Cost Among Chronic Patients in Medicaid. Medicare & Medicaid Research Review 2012;**2**(3):E1-E13.

2. National Institute for Health and Care Excellence (NICE). *Type 2 diabetes in adults: management (NG28)*. London, UK: NICE, December 2015.

3. National Institute for Health and Care Excellence (NICE). British National Formulary (BNF). London, UK: NICE, 2016.

4. National Institute for Health and Care Excellence (NICE). *Myocardial infraction: cardiac rehabilitation and prevention of further MI (CG172)*. London, UK: NICE, November 2013.

5. Hollingworth W, Devine EB, Hansen RN, et al. The impact of e-prescribing on prescriber and staff time in ambulatory care clinics: a time motion study. J Am Med Inform Assoc 2007;**14**(6):722-30.

6. Westbrook JI, Li L, Georgiou A, et al. Impact of an electronic medication management system on hospital doctors' and nurses' work: a controlled pre-post, time and motion study. J Am Med Inform Assoc 2013;**20**(6):1150-8.

7. Franklin BD, O'Grady K, Donyai P, et al. The impact of a closed-loop electronic prescribing and administration system on prescribing errors, administration errors and staff time: a before-and-after study. Qual Saf Health Care 2007;**16**(4):279-84.

8. Laurant M, Reeves D, Hermens R, et al. Substitution of doctors by nurses in primary care. Cochrane Database Syst Rev 2005(2):CD001271.

9. Shum C, Humphreys A, Wheeler D, et al. Nurse management of patients with minor illnesses in general practice: multicentre, randomised controlled trial. BMJ 2000;**320**(7241):1038-43.

10. Venning P, Durie A, Roland M, et al. Randomised controlled trial comparing cost effectiveness of general practitioners and nurse practitioners in primary care. BMJ 2000;**320**(7241):1048-53.

For peer review only

For peer review only - http://bmjopen.bmj.com/site/about/guidelines.xhtml

STROBE Statement—checklist of items that should be included in reports of observational studies

	Item No	Recommendation
**Title and abstract**	1	(*a*) Indicate the study's design with a commonly used term in the title or the abstract
		(*b*) Provide in the abstract an informative and balanced summary of what was done and what was found
**Introduction**		
Background/rationale	2	Explain the scientific background and rationale for the investigation being reported
Objectives	3	State specific objectives, including any prespecified hypotheses
**Methods**		
Study design	4	Present key elements of study design early in the paper
Setting	5	Describe the setting, locations, and relevant dates, including periods of recruitment, exposure, follow-up, and data collection
Participants	6	(*a*) *Cohort study*—Give the eligibility criteria, and the sources and methods of selection of participants. Describe methods of follow-up<br>*Case-control study*—Give the eligibility criteria, and the sources and methods of case ascertainment and control selection. Give the rationale for the choice of cases and controls<br>*Cross-sectional study*—Give the eligibility criteria, and the sources and methods of selection of participants
		(*b*) *Cohort study*—For matched studies, give matching criteria and number of exposed and unexposed<br>*Case-control study*—For matched studies, give matching criteria and the number of controls per case
Variables	7	Clearly define all outcomes, exposures, predictors, potential confounders, and effect modifiers. Give diagnostic criteria, if applicable
Data sources/ measurement	8*	For each variable of interest, give sources of data and details of methods of assessment (measurement). Describe comparability of assessment methods if there is more than one group
Bias	9	Describe any efforts to address potential sources of bias
Study size	10	Explain how the study size was arrived at
Quantitative variables	11	Explain how quantitative variables were handled in the analyses. If applicable, describe which groupings were chosen and why
Statistical methods	12	(*a*) Describe all statistical methods, including those used to control for confounding
		(*b*) Describe any methods used to examine subgroups and interactions
		(*c*) Explain how missing data were addressed
		(*d*) *Cohort study*—If applicable, explain how loss to follow-up was addressed<br>*Case-control study*—If applicable, explain how matching of cases and controls was addressed<br>*Cross-sectional study*—If applicable, describe analytical methods taking account of sampling strategy
		(*e*) Describe any sensitivity analyses

Continued on next page

For peer review only

**Results**		
Participants	13*	(a) Report numbers of individuals at each stage of study—eg numbers potentially eligible, examined for eligibility, confirmed eligible, included in the study, completing follow-up, and analysed
		(b) Give reasons for non-participation at each stage
		(c) Consider use of a flow diagram
Descriptive data	14*	(a) Give characteristics of study participants (eg demographic, clinical, social) and information on exposures and potential confounders
		(b) Indicate number of participants with missing data for each variable of interest
		(c) *Cohort study*—Summarise follow-up time (eg, average and total amount)
Outcome data	15*	*Cohort study*—Report numbers of outcome events or summary measures over time
		Case-control study—Report numbers in each exposure category, or summary measures of exposure
		Cross-sectional study—Report numbers of outcome events or summary measures
Main results	16	(*a*) Give unadjusted estimates and, if applicable, confounder-adjusted estimates and their precision (eg, 95% confidence interval). Make clear which confounders were adjusted for and why they were included
		(*b*) Report category boundaries when continuous variables were categorized
		(*c*) If relevant, consider translating estimates of relative risk into absolute risk for a meaningful time period
Other analyses	17	Report other analyses done—eg analyses of subgroups and interactions, and sensitivity analyses
**Discussion**		
Key results	18	Summarise key results with reference to study objectives
Limitations	19	Discuss limitations of the study, taking into account sources of potential bias or imprecision. Discuss both direction and magnitude of any potential bias
Interpretation	20	Give a cautious overall interpretation of results considering objectives, limitations, multiplicity of analyses, results from similar studies, and other relevant evidence
Generalisability	21	Discuss the generalisability (external validity) of the study results
**Other information**		
Funding	22	Give the source of funding and the role of the funders for the present study and, if applicable, for the original study on which the present article is based

*Give information separately for cases and controls in case-control studies and, if applicable, for exposed and unexposed groups in cohort and cross-sectional studies.

Note: An Explanation and Elaboration article discusses each checklist item and gives methodological background and published examples of transparent reporting. The STROBE checklist is best used in conjunction with this article (freely available on the Web sites of PLoS Medicine at http://www.plosmedicine.org/, Annals of Internal Medicine at http://www.annals.org/, and Epidemiology at http://www.epidem.com/). Information on the STROBE Initiative is available at www.strobe-statement.org.

For peer review only

For peer review only - http://bmjopen.bmj.com/site/about/guidelines.xhtml
